# Small RNA sequencing of field *Culex* mosquitoes identifies patterns of viral infection and the mosquito immune response

**DOI:** 10.1038/s41598-023-37571-6

**Published:** 2023-06-30

**Authors:** Steven M. Abel, Zhenchen Hong, Desiree Williams, Sally Ireri, Michelle Q. Brown, Tianyun Su, Kim Y. Hung, Jennifer A. Henke, John P. Barton, Karine G. Le Roch

**Affiliations:** 1 Department of Molecular, Cell and Systems Biology, Center for Infection Disease and Vector Research, University of California, Riverside, CA 92521 USA; 2 Department of Physics and Astronomy, University of California, Riverside, CA 92521 USA; 3 West Valley Mosquito & Vector Control District, Ontario, CA 91761 USA; 4 Coachella Valley Mosquito & Vector Control District, Indio, CA 92201 USA

**Keywords:** Microbial genetics, RNAi, Viral epidemiology, Viral host response

## Abstract

Mosquito-borne disease remains a significant burden on global health. In the United States, the major threat posed by mosquitoes is transmission of arboviruses, including West Nile virus by mosquitoes of the *Culex* genus. Virus metagenomic analysis of mosquito small RNA using deep sequencing and advanced bioinformatic tools enables the rapid detection of viruses and other infecting organisms, both pathogenic and non-pathogenic to humans, without any precedent knowledge. In this study, we sequenced small RNA samples from over 60 pools of *Culex* mosquitoes from two major areas of Southern California from 2017 to 2019 to elucidate the virome and immune responses of *Culex*. Our results demonstrated that small RNAs not only allowed the detection of viruses but also revealed distinct patterns of viral infection based on location, *Culex* species, and time. We also identified miRNAs that are most likely involved in *Culex* immune responses to viruses and *Wolbachia* bacteria, and show the utility of using small RNA to detect antiviral immune pathways including piRNAs against some pathogens. Collectively, these findings show that deep sequencing of small RNA can be used for virus discovery and surveillance. One could also conceive that such work could be accomplished in various locations across the world and over time to better understand patterns of mosquito infection and immune response to many vector-borne diseases in field samples.

## Introduction

Transmission of arboviruses to humans by mosquitoes is a persistent public health threat around the world. In the United States, *Culex* mosquitoes transmit arboviruses that are endemic in several states. These notably include West Nile virus (WNV), which caused ~ 2500 human cases of disease annually in the U.S. between 1999 and 2019 in addition to many times more asymptomatic infections, and St. Louis encephalitis virus, which also causes a small number of cases annually, including periodic outbreaks^1–3^. WNV, considered the most prevalent cause of viral encephalitis worldwide, reached New York City in 1999 and spread to the rest of North America within four years, including California in 2003. Human infections can sometimes result in severe neuroinvasive disease, especially in older patients and those with chronic medical conditions^3^.

Many viruses that have been detected in mosquitoes do not infect humans but do establish persistent infections in the mosquito, and evoke small RNA immune responses^4,5^. Viruses in this diverse group include insect-specific viruses (ISVs)^6^ and those that can be transmitted to non-human organisms. Little is known about many of these viruses, or their effect on transmission of arboviruses by mosquitoes. Recent studies have presented evidence that some ISVs may decrease arbovirus loads and transmission^7–9^ similarly to what is observed with infection by the *Wolbachia* bacterium. *Wolbachia* is a genus of intracellular bacteria that has been shown, when introduced into non-native host *Ae. aegypti*, to significantly reduce the mosquito’s ability to transmit dengue, Zika, and other RNA viruses to humans^10–12^. The potential mechanisms of *Wolbachia*-mediated antiviral effects are not completely clear, but data suggest some evidence of competition for resources between the virus, host, and *Wolbachia*^13,14^ as well as use of host microRNAs by the bacterium to contribute to virus inhibition^15^. If ISVs have some effects on arbovirus infection and transmission^7–9,16,17^, they could be used as biological control mechanisms or novel vaccine platforms by exploiting the limited host range of ISVs to protect against dangerous viruses infecting humans^18^. Furthermore, constant monitoring of viruses may allow us to detect the re-emergence of arboviruses transmissible to humans.

In insects including mosquitoes, the small RNA interference (RNAi) system has been shown to play a central role in defense against viruses, most prominently the exogenous small interfering RNA (siRNA) pathway^19,20^. Replicative intermediates in the form of dsRNA are often generated during viral infection, and these intermediates can be processed into ~ 21-nt long siRNAs by Dicer-2. The siRNAs are loaded onto the RNA-induced silencing complex (RISC) and guide it to complementary, invading viral sequences, which will then be degraded^21,22^. By contrast, the PIWI-interacting RNA (piRNA) pathway has a well-established role in silencing transposons to maintain germline integrity^23^. However, piRNAs, which are generally ~ 24–29 nt in length, have also been implicated in antiviral activity in mosquitoes, although this activity is not yet well-understood^24–28^. Interestingly, this expanded piRNA activity does not seem to be present in *Drosophila*, despite mosquitoes and fruit flies being in the same order, Diptera. Some mosquitoes, particularly *Culex* and *Aedes* species, possess an expanded repertoire of Piwi-clade proteins as compared to *Drosophila*. Some of the proteins are expressed in somatic cells as well as follicular cells, and when purified were found to be associated with virus-derived sequences^26,29^. piRNAs can be produced through the primary Zucchini (Zuc)—mediated biogenesis pathway, which generates antisense piRNAs with a 1U bias^30,31^ or through the “ping-pong cycle”, where primary piRNAs are used to generate sense piRNAs with a 10A bias and further 1U antisense piRNAs^32^. Virus-derived piRNAs have been shown to have the antisense 1U and/or sense 10A nucleotide biases^24–26,33^, although the mechanisms of viral piRNA generation remain unknown. Finally, a separate class of small RNAs, miRNAs, are ~ 22-nt long, are transcribed from the host genome, and have been demonstrated as critical components of gene regulation by binding to cellular mRNAs to control their translation, stability, or decay^34^.

RNA sequencing has been used to detect viruses in many species, including mosquitoes^35–38^. In our study, we aimed to use total small RNA extracted from whole mosquitoes to not only sample the virome of mosquitoes but also to analyze patterns of viral infection and immune signatures in these mosquitoes. We therefore deep sequenced 63 pools of *Culex* mosquitoes, 58 of them field-caught, and showed snapshots of the *Culex* virome over a three-year period in southern California. We also examined the patterns and correlation of viral infection based on location, year, and mosquito species. Furthermore, as the goal of this study was not only to discover viruses, but rather to analyze the abundance of and host response to viruses, we also mapped reads to the *Culex* genome to elucidate miRNA responses to both viruses and *Wolbachia*. Finally, we generated size profiles and virus genome coverage plots from the small RNAs mapping to individual viruses to analyze induction of small RNA pathways such as siRNA and piRNA in response to viral infection in field mosquitoes. Taken together, the results demonstrate the power of our approach, which could be used not only for virus discovery, surveillance, and epidemiology, but also to improve our understanding of mosquito immune response to many vector-borne diseases around the world.

## Results

### Detection of viruses in *Culex* samples based on de novo assembly of small RNA reads

**Figure 1:**
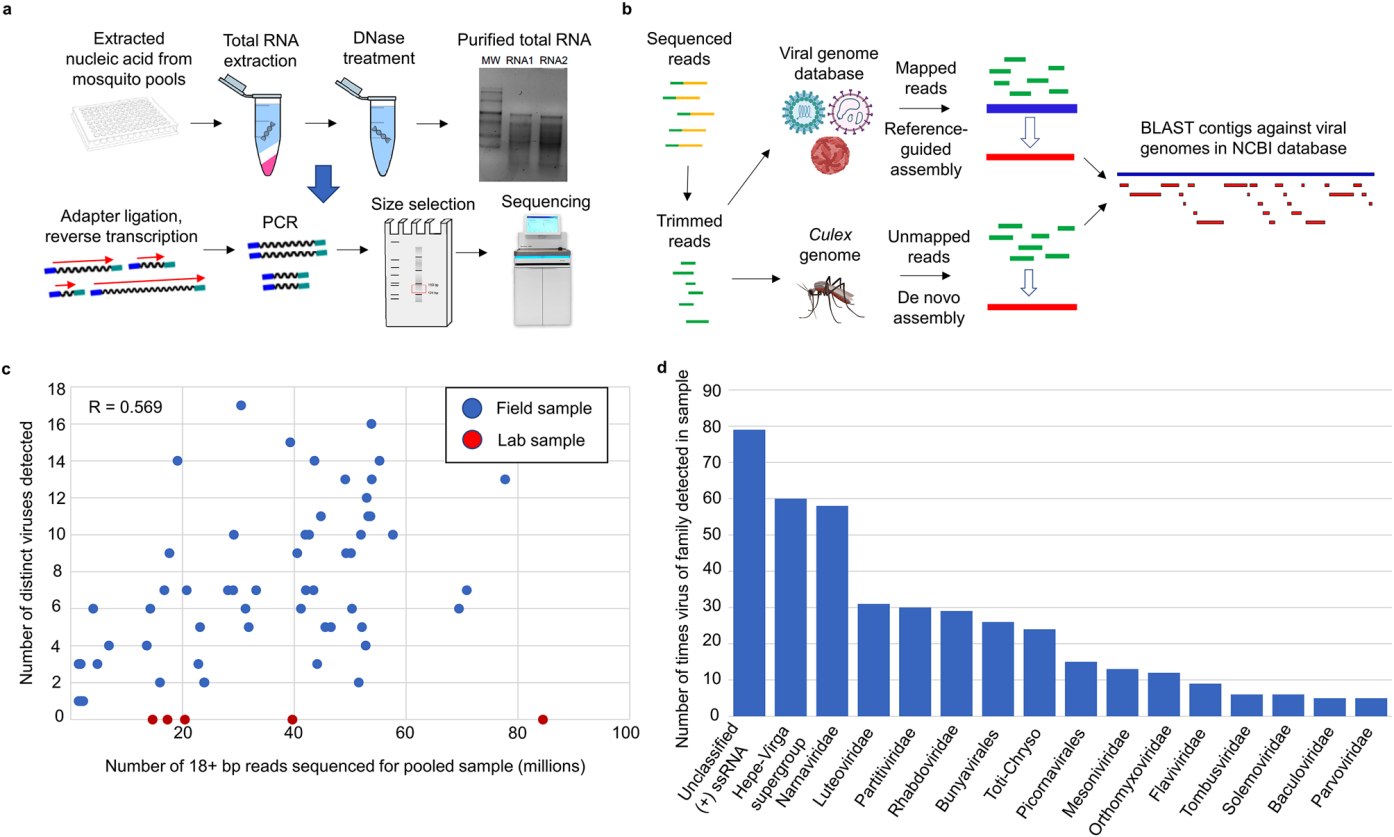
Virus detection using small RNA libraries. (**a**) Schematic representation of the experimental protocol. RNA was extracted from mosquito pools, reverse transcribed, PCR amplified, size selected for small RNA, and sequenced. Pool sizes are listed in Supplementary Table S1. (**b**) Schematic representation of virus detection including VirusDetect. Reads were assembled into contigs in two ways and compared to a viral genome database by BLAST. (**c**) Number of known mosquito viruses detected in a sample vs. number of sequenced for that sample (Spearman’s R = 0.569 for all samples). (**d**) High nucleotide identity (> 90%) virus detections in *Cx. quinquefasciatus* samples by taxonomic group. Hepe-Virga supergroup and Toti-chryso are loose classifications of related virus families.

We sequenced and analyzed small RNA from 58 pools of either *Cx. quinquefasciatus* or *Cx. tarsalis* mosquitoes from the Inland Empire region of southern California, as well as five *Cx. quinquefasciatus* pools originating from laboratory strains. The most common pool size was 50 or near to this, but pool sizes varied (see Supplementary Table S1). Our experimental pipeline is summarized in Fig. 1a, and the computational pipeline for virus detection using VirusDetect^39^ is displayed in Fig. 1b. Agarose gel pictures showing extracted total RNA from mosquito pools are shown in Supplementary Fig. S1.

The pools received an average of 57.9 million reads per sample. The number of sequenced reads directly correlated to the number of distinct viruses that were detected in field samples (Spearman’s R coefficient = 0.569). We detected an average of 7 distinct viruses in each sample. Despite high quality sequencing reads for five laboratory samples (average of 72.7 million reads), we did not detect any known mosquito-associated viruses in these samples (Fig. 1c), suggesting that lab-grown mosquitoes are not exposed to pathogens as field mosquitoes are. This should be considered when using lab mosquitoes to study viruses. Due to the varying number of sequenced reads between samples, normalization accounting for read number was done whenever samples were compared in downstream analysis.

For most samples, there were several high-identity matches by nucleotide alignment (blastn), which we considered to be high-confidence virus detections as these sequences were closely related to known reference genomes. Many samples also had more distant matches that were detected only through virtual translation of the sequences in the six reading frames (blastx). The list of viruses detected by blastn with 90% nucleotide identity or higher can be found in Supplementary Table S2, while viruses detected only by blastx with 50% amino acid identity or higher are in Supplementary Table S3. The numbers of 90%+ blastn virus detections by virus family/classification in field samples are shown in Fig. 1d. Virus families detected, separated by mosquito species (*Cx. quinquefasciatus* vs *Cx. tarsalis)* are shown in Supplementary Fig. S2. By far the most abundant single virus detected was *Culex* narnavirus 1, which was present in every field sample of both species. Also common were viruses from the Hepe-Virga supergroup^35^, a group of (+)ssRNA viruses that has been loosely defined and only recently characterized, reflecting the lack of clear understanding around invertebrate viruses. Supporting this as well is the high prevalence of unclassified (+)ssRNA viruses which could not be placed into any defined families, such as *Bunyavirales*, *Rhabdoviridae*, and *Flaviviridae*, which are well-known to contain ISVs or arboviruses. Interestingly, we were also able to detect viruses thought to only infect plants (e.g. *Tombusviridae*, *Tymoviridae*, and *Luteoviridae*). For these viruses, as discussed later, we have strong evidence of specific siRNA responses (Supplementary Fig. S3, https://github.com/Sabel14/MosquitoSmallRNA_Supplemental_AndCustomScripts), suggesting that they may indeed infect mosquitoes. Many of the viruses detected have widespread geographical range, as some of them were found in other parts of the world including China^35^, Mexico^40^, and Colombia^41^, suggesting many of the same or very similar *Culex* viruses are found throughout the world.

### Clustering and patterns of virus-mapped small RNA quantity in mosquito samples

**Figure 2:**
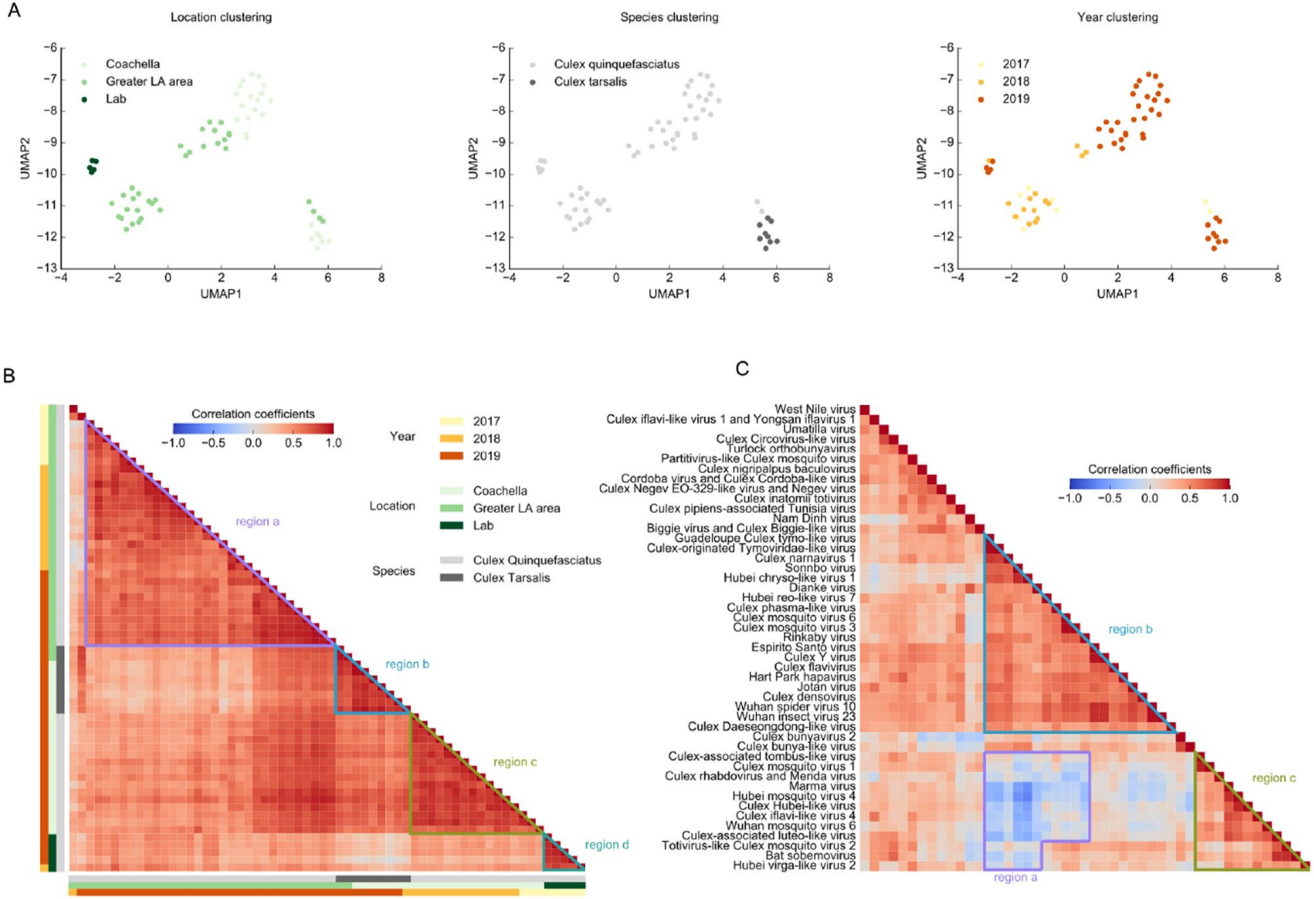
Clustering and correlation of mosquito pools by virus small RNA quantities. (**a**) Clustering of mosquito pool samples by virus small RNA quantities using the dimension reduction method UMAP. The three plots differ only by the sample property used to color the data points. (**b**) Pearson correlation matrix of mosquito pool samples by virus small RNA quantities. Sample properties are labeled to the left and below the matrix, and regions of high correlation are denoted. (**c**) Pearson correlation matrix of detected viruses by reads mapped from all samples. Regions of low and high correlation are denoted.

To identify factors affecting viral infection, we used direct mapping of reads to viral genomes (read counts in Supplementary Table S1) and clustered our samples using UMAP^42^, a manifold learning technique for dimension reduction (see Methods for details). The resulting numbers of mapped reads represent a combination of viral abundance and intensity of the mosquito immune response, and will be referred to as small RNA quantity. Results show that the most obvious factors determining small RNA quantity in a sample were location and mosquito species (data points for *Cx. tarsalis* cluster apart from those for *Cx. quinquefasciatus*). This was true even for samples collected over multiple years (Fig. 2a). Year itself as a factor also appears to drive sample clustering but is closely tied to location. As another way to visualize relationships based on small RNA quantity, we generated Pearson correlation matrices^43^ between samples and between viruses. The sample correlation matrix (Fig. 2b) displays which samples tend to contain the same viruses. Results are similar to those obtained by UMAP. Broadly, blocks of high correlation represented, respectively, Greater LA *Cx. quinquefasciatus* (region a), both locations’ *Cx. tarsalis* (region b), Coachella Valley *Cx. quinquefasciatus* (region c), and lab (region d) samples.

To detect possible virus co-occurrence or suppression in our samples, we generated a virus correlation matrix (Fig. 2c) using Pearson coefficients for pairs of detected viruses based on their read frequencies across all mosquito pool samples. A positive correlation would mean two viruses tended to infect and generate small RNA in the same samples, while a negative one would mean they are found together in the same sample less often than expected by chance. Negative correlation coefficients were observed between two groups of viruses, as detected in region a. The coefficients in this region range between -0.67 and 0.28, with a median of − 0.15. Considering only viruses involved in the most negative correlations, the first group includes Guadeloupe *Culex* tymo-like virus, *Culex*-originated *Tymoviridae*-like virus, Sonnbo virus, Hubei chryso-like virus 1, and Dianke virus, while the second includes Marma virus, Hubei mosquito virus 4, *Culex* Hubei-like virus, *Culex* iflavi-like virus 4, Wuhan mosquito virus 6, and *Culex*-associated luteo-like virus. This suggests that these groups of viruses could exclude each other within the same mosquitoes, potentially providing research direction about virus exclusion, to narrow down the range of possible exclusion candidates. Scatterplots showing frequencies of two viruses for all samples show that the negative correlations are not the result of outlier samples but rather general trends (Supplementary Fig. S4). There are also blocks of notably high correlation within two groups of viruses (regions b and c).

Virus correlation matrices including samples collected from the Coachella Valley or *Cx. tarsalis* exhibit patterns that differ from the overall matrix (Supplementary Fig. S5). In *Cx. tarsalis*, WNV is only strongly positively correlated with a select group of other viruses and has a very weak or negative correlation with most. This contrasts with what was observed in *Cx. quinquefasciatus*, where WNV was notably positively correlated with almost all viruses. Thus, it is possible that WNV interacts differently with other viruses depending on the mosquito species, although other possibilities exist such as *Cx. quinquefasciatus* being a more competent vector than *Cx. tarsalis*. These data will need to be further validated as additional samples may provide further insight into viral co-infection patterns.

### Small RNA derived from the mosquito genome reveals miRNAs likely to be related to pathogen infection

**Figure 3:**
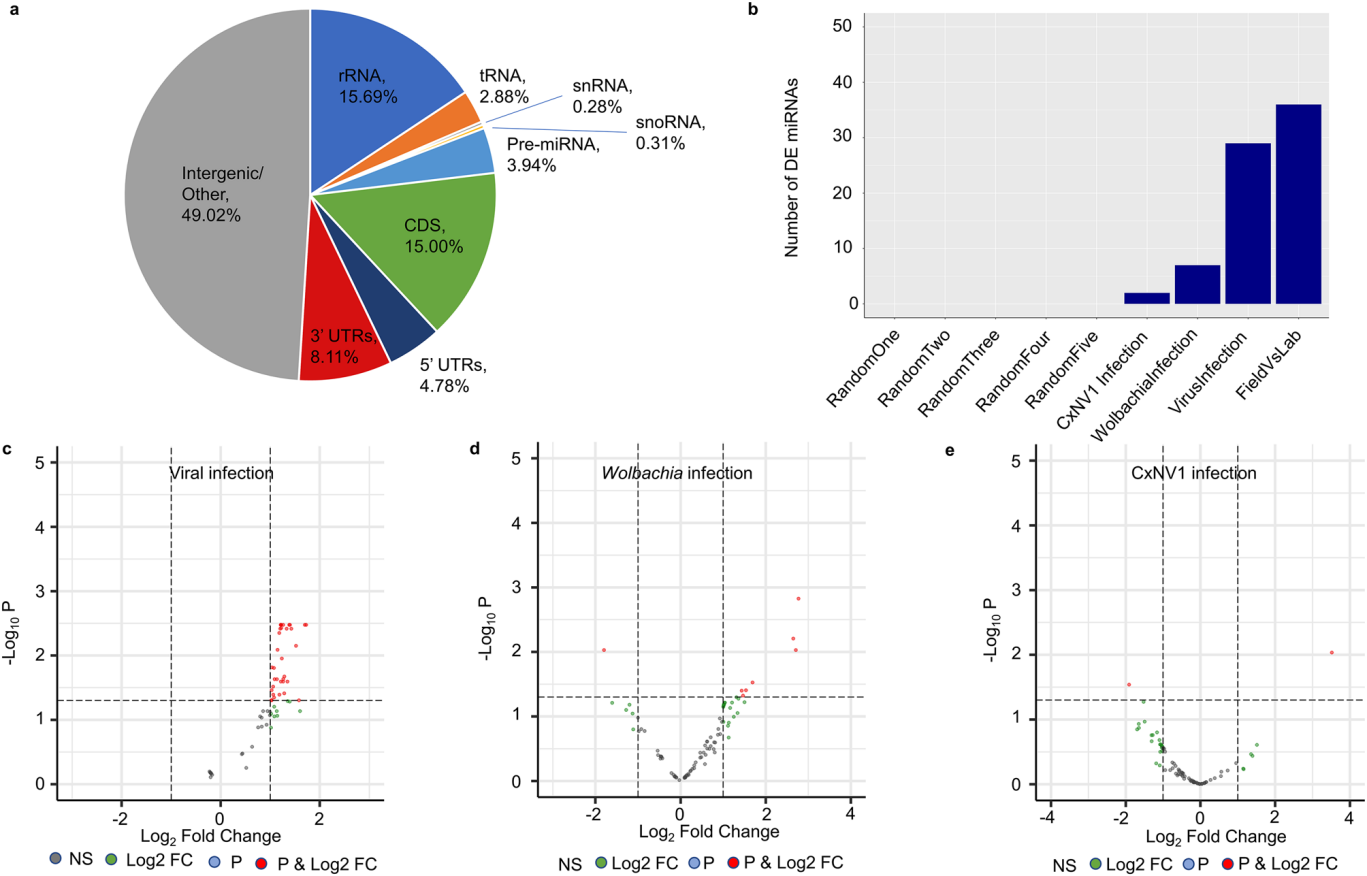
Analysis of small RNA derived from the *Culex* genome. (**a**) Percentages of small RNA reads from all *Cx. quinquefasciatus* field samples mapping to each type of genomic feature in the mosquito genome. (**b**) Numbers of miRNA genes determined as differentially expressed for each comparison of field *Cx. quinquefasciatus* samples, with field vs. lab as a point of reference for these comparisons. (**c**–**e**) Comparison of miRNA expression in samples with higher abundance of (**c**) viruses, (**d**) *Wolbachia*, or (**e**) CxNV1 against those with lower abundance. Volcano significance plots have adjusted P-value cutoff of 0.05 and log2fold change cutoff of 1. NS: not significant. Log2 FC: significant by log2fold change only (threshold ± 1). P: significant by adjusted P-value only (threshold 0.05). P & Log2 FC: significant by both adjusted P-value and log2fold change.

To investigate the *Culex* response to infection, we explored small RNA reads that mapped to the *Cx. quinquefasciatus* genome (CpipJ2 assembly)—*Cx. tarsalis* samples were not included in this analysis due to the lack of an extensively annotated genome assembly. Approximately 19% of *Culex*-aligned reads mapped to rRNA, tRNA, snRNA, or snoRNA genes, while 32% mapped to pre-miRNA or protein-coding genes, either in coding regions or putative untranslated regions (UTRs) (Fig. 3a). The remaining 49% mapped to intergenic regions, perhaps representing unannotated transcripts such as novel pre-miRNA genes or lncRNAs. A higher percentage of reads mapped to the antisense of the 3′ UTRs as compared to CDSs and 5′ UTRs, and to 3′ UTRs in general when normalized by total feature length (Supplementary Fig. S6), indicating that our reads are most likely enriched for miRNAs and further validating our methodology as small RNAs, particularly miRNAs, are known to bind to the antisense of the 3′ UTRs of targeted genes to regulate transcription at the post-transcriptional level^44^.

Next, we performed multiple comparisons in which we segregated all *Cx. quinquefasciatus* samples into two groups based on chosen sample attributes and compared the groups against each other. This was done using DESeq2^45^, a software often used for RNA-seq differential expression analysis, using only sense-mapped reads and restricting the analysis to miRNA genes. DESeq2 normalizes for library size (number of reads) and has been successfully used in various fields for differential expression of small RNAs including miRNAs^46–48^. Our number of samples allowed for a higher number of replicates than typical RNA-seq experiments (48 samples in each field vs. field comparison, 53 samples in a field vs. lab comparison). As a negative control, all 48 *Cx. quinquefasciatus* field samples were randomly assigned into two groups five separate times. Each time, 0 miRNAs were differentially expressed between the two groups (Fig. 3b). From this, we were confident that any miRNAs that would be called as differentially expressed between selected groups would be due to the chosen factors and not statistical noise.

We first compared samples that were highly infected by viruses against those that were lowly infected, using a threshold of 0.049% of sequenced reads aligning to virus genomes, while controlling for the effects of location and year of collection by including these factors in the DESeq2 generalized linear model (Fig. 3c). For this analysis, we however excluded *Culex* narnavirus 1, due to its extremely high abundance in all of our samples, and instead analyzed its effect separately (see below). We identified thirty-five pre-miRNA genes that were significantly upregulated in highly infected samples, including twenty-nine unique miRNAs (Fig. 3c). The full list of upregulated miRNAs is available in Supplementary Table S4. Interestingly, fourteen of the upregulated miRNAs have already been tied to pathogen infection in previous experiments (see Discussion for details). To assess the putative targets of the top 20 highly expressed of the differentially expressed miRNAs, we used sRNAtoolbox miRNAconsTarget^67^, a software that combines four different miRNA target prediction algorithms. The list of putative targets is available in Supplementary Table S5. Gene ontology (GO) enrichment of the targeted genes (Supplementary Table S5), focusing on those agreed upon by at least 2 of the 4 prediction algorithms used, identifies function in translation and cellular respiration as being enriched among the potential identified targets. When we restricted the enrichment to those agreed upon by at least 3 of 4 algorithms, GO enrichment identifies several genes involved in innate immunity, validating further our initial results.

We next examined the effect of *Wolbachia* infection on miRNAs in *Culex* mosquitoes, while controlling for the effects of location and year of collection (Fig. 3d). High/low infection by *Wolbachia*, as determined by a threshold of 6.34% (the median percentage) of *Culex*-unmapped reads aligned to the *Wolbachia* genome, was associated with a lower number of differentially expressed miRNA genes (8, with 7 of these being unique miRNAs) than infection by viruses (Supplementary Table S6). Two of the seven differentially expressed miRNAs, miR-1889 and miR-12, have been previously associated with *Wolbachia* infection in mosquitoes^49,50^ (see Discussion). These results suggest that *Wolbachia* infection induces a more limited but significant miRNA response in the mosquito as compared to viral infection.

As *Culex* narnavirus 1 accounted by itself for 38.6% of virus-mapped reads, we generated a separate analysis between samples with high and low abundance of this virus (Fig. 3e), determined by a threshold of 0.171% (the median percentage) of sequenced reads aligned to the CxNV1 genome. However, only two miRNAs were detected as differentially expressed, with miR-1889 upregulated and miR-277 downregulated (Supplementary Table S7). Interestingly, miR-1889 was upregulated in both high-*Wolbachia* and high-CxNV1 groups, suggesting a possible general immune function. The effect of CxNV1 infection on miRNAs, while seemingly present to some degree, will need to be explored in future experiments.

### Small RNA responses to specific viruses by size profile and genome coverage analysis

Next, we investigated the specific mosquito immune response to individual viruses by examining the size and other properties of reads mapped to each particular virus. The size profiles of the mapped reads, their nucleotide biases, and patterns of sense and antisense genome coverage can be combined to gauge the extent to which siRNA and piRNA response pathways are used in *Culex* against each virus. Because only reads which did not map to the *Culex* genome were used, we can reasonably assume that most observed siRNAs and piRNAs are virus-derived rather than encoded by a viral integration segment in the mosquito genome.

**Figure 4:**
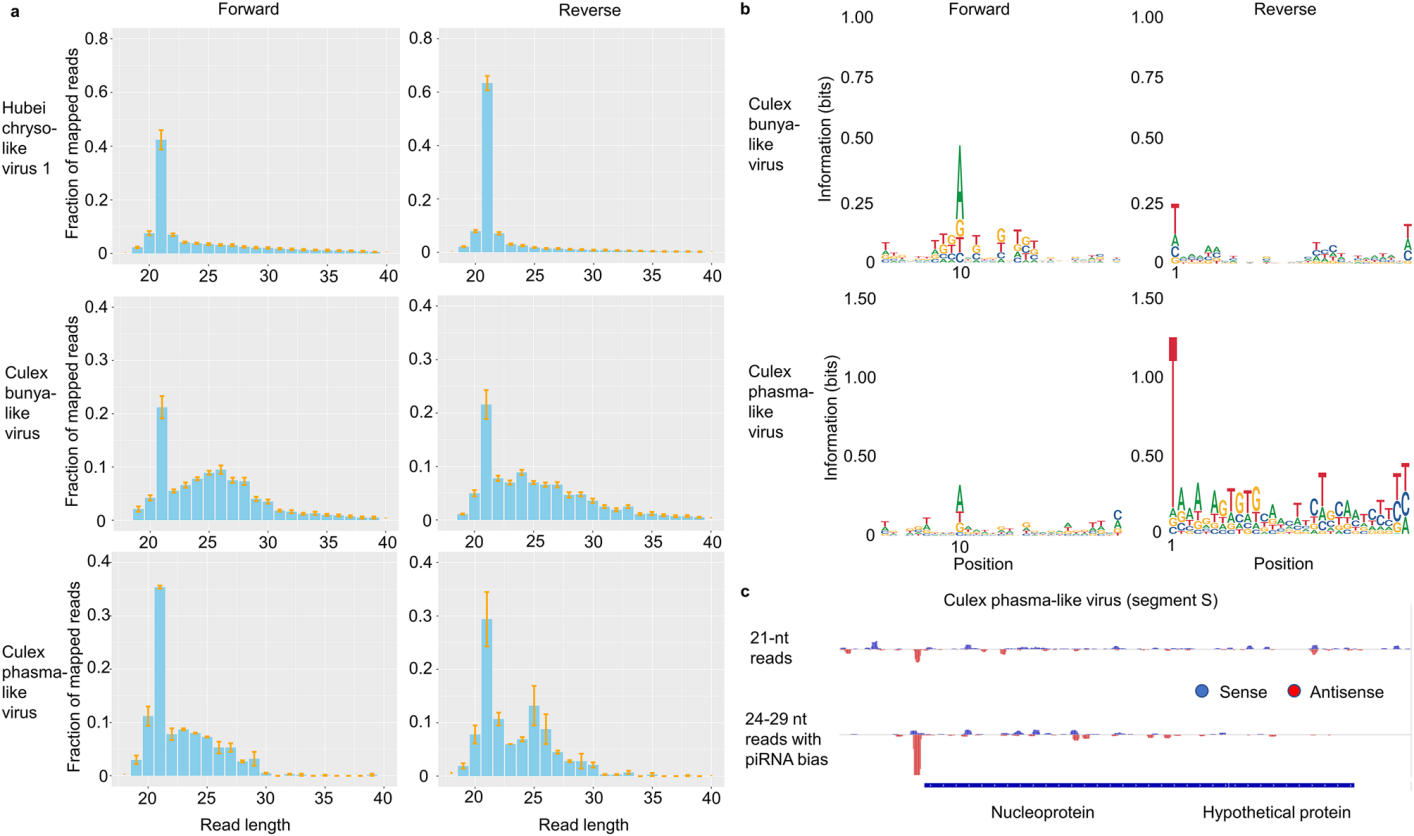
Small RNA responses of field *Culex* mosquitoes against specific viruses. (**a**) Examples of small RNA size profiles showing percentages of mapped reads of each size. HCLV1 displays a strong siRNA response (21-nt peaks), while CbunLV and CphasLV display both siRNA and piRNA responses (24–29 nt enrichment). Percent values are averages across all samples in which the virus was detected. Error bars show the average ± standard error for that small RNA size across all samples. (**b**) Sequence logo plots showing nucleotide bias for 24–29 nt reads mapping to individual viruses. Bias is indicative of piRNA generation by the ping-pong cycle. (**c**) Likely siRNAs and piRNAs mapped to *Culex* phasma-like virus segment S. Only 24–29 nt reads with 1U (for antisense reads) or 10A (for sense reads) are included in the piRNA track.

The small RNA size profiles that we detected for each virus are displayed in Fig. 4a and Supplementary Fig. S3. A specific siRNA response was observed for many viruses, with Hubei chryso-like virus 1 being a very clear example in which ~ 50% of the total mapped reads were 21 nt in length. For *Culex* bunya-like virus (CbunLV) and *Culex* phasma-like virus (CphasLV), in addition to the 21-nt peak, we detected a clear enrichment for read lengths of 24–29 nt. To validate that the detected read length of 24–29 reads represent a specific piRNA pathway response, we confirmed a sequence bias for an A in the 10th position of the forward reads and for a T in the 1st position of the reverse reads of this size range, indicative of piRNA generation by the ping-pong cycle (Fig. 4b). We also confirmed that, for CbunLV and CphasLV, there are far more 10-nt overlaps between reads with the ping-pong signature nucleotides than those without, demonstrating additional evidence for this mode of synthesis in the piRNA response pathway (Supplementary Fig. S7). Similar profiles were also detected against Turlock orthobunyavirus and Hart Park hapavirus, suggesting the activation of the piRNA ping-pong pathway against these viruses as well (Supplementary Figs. S3 and S8). Altogether, clear evidence for the activation of the ping-pong piRNA response pathway was limited to viruses with negative-polarity single-stranded RNA genomes. For *Culex* phasma-like virus segment S, we found that likely piRNAs (24–29 nt, with 1U for antisense reads or 10A for sense reads) target one region directly upstream of the nucleoprotein gene (Fig. 4c). By contrast, the 21-nt reads that characterize the siRNA response pathway were scattered throughout the virus genome, suggesting that these pathways can specifically target distinct regions in the genome. For this particular segment, a significant number of siRNAs targeted the same site as piRNAs, but this was not the case for all viruses, as mentioned below.

**Figure 5:**
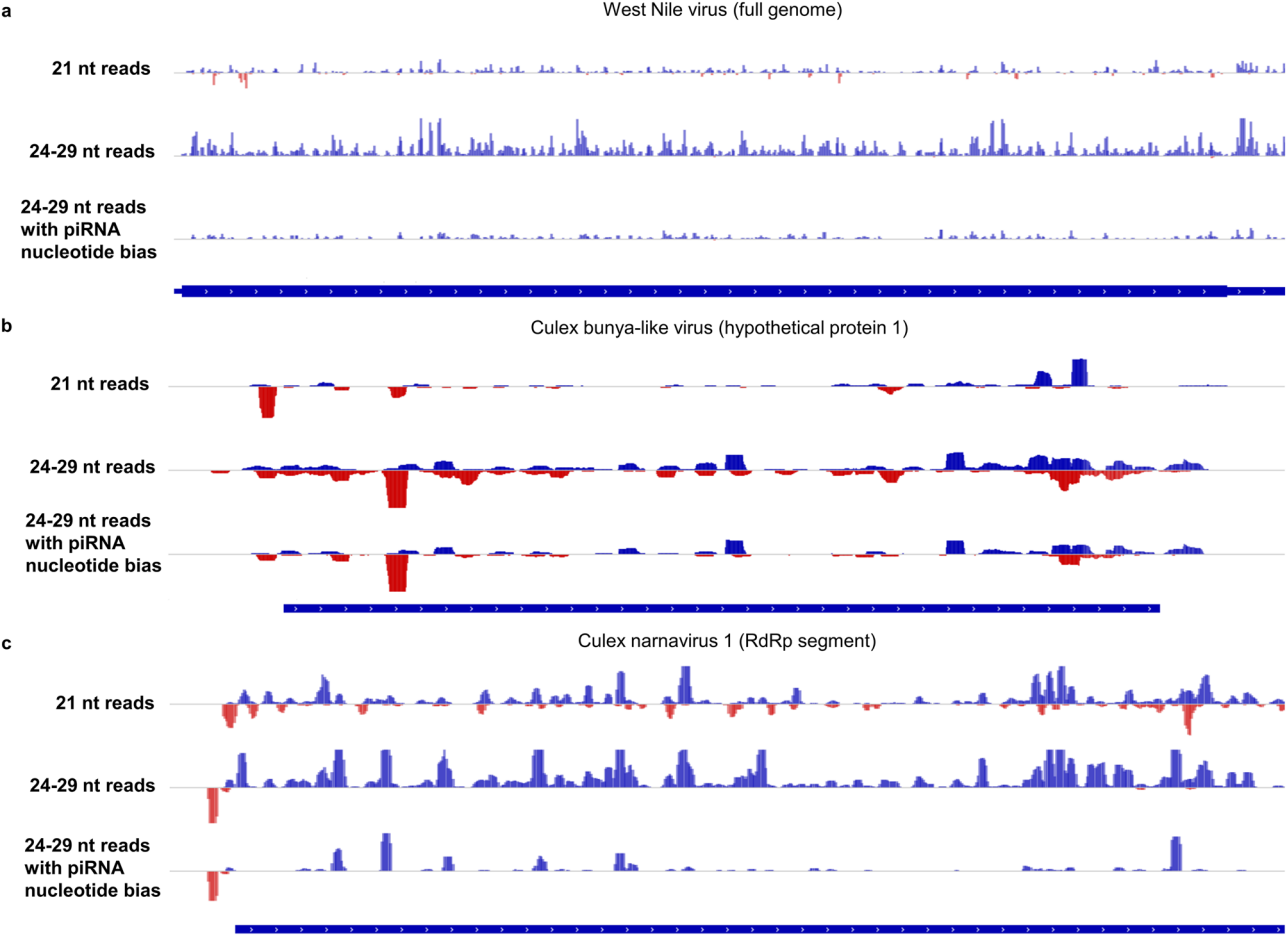
Virus coverage plots showing evidence of siRNAs and/or piRNAs in three viruses. (**a**) Sense-mapped reads are shown in the upper half of each track in blue, while antisense-mapped reads are shown in the bottom half in red. Reads shown are from all pools in which the virus was detected by VirusDetect. (**a**) The presence of antisense 21-nt reads but not antisense 24–29 nt reads, as well as lack of nucleotide bias in 24–29 nt reads, suggest siRNA but not piRNA generation against WNV. (**b**) Both sense and antisense reads for both size ranges are generated in abundance against the *Culex* bunya-like virus genomic region shown, and the 24–29 nt reads include a high percentage with the piRNA nucleotide bias. (**c**) Antisense 21-nt reads are abundant against the entire *Culex* narnavirus 1 genome, while antisense 24–29 nt reads are confined to a strong peak upstream of the coding region and many possess the piRNA nucleotide bias. This suggests generation of viral piRNAs without a detectable ping-pong signature.

These results are expanded upon by examining patterns of sense and antisense small RNA coverage for each virus, which support the findings of siRNAs and piRNAs discussed above and also suggest production of piRNAs without ping-pong generation against some viruses. For WNV, a positive-sense RNA virus, antisense 21-nt reads can be found in multiple genomic regions, but there are virtually no antisense 24–29 nt reads (Fig. 5a), agreeing with the idea that siRNAs but not piRNAs are generated against WNV as suggested by its size profile and lack of 1U or 10A nucleotide bias (Supplementary Fig. S3). This agrees with observations previously made for WNV-infected mosquito cell lines^4^. The coverage plot for a genomic region of *Culex* bunya-like virus confirms the extensive production of siRNAs and piRNAs against it, due to the abundant sense and antisense reads of both size ranges, including many 24–29 nt reads with ping-pong nucleotide bias (Fig. 5b). Finally, the coverage plot for *Culex* narnavirus 1 reveals new information about this virus (Fig. 5c), whose small RNA size profile and lack of ping-pong nucleotide bias suggested only siRNA production against it (Supplementary Fig. S3). There is a distinct peak of antisense reads upstream of the coding region, a similar pattern observed in *Culex* phasma-like virus (Fig. 4c), most of which have the piRNA 1U bias. In this case, siRNAs do not target the same site as piRNAs.

Coverage plots also suggest possible piRNA production for other viruses without a detectable ping-pong signature or obvious size profile. This can be suggested either by distinct peaks of antisense 24–29 nt reads as for CxNV1 and Hubei mosquito virus 4, or widespread antisense coverage with 1U bias, as for Wuhan insect virus 23 and Wuhan spider virus 10 (Supplementary Fig. S3). Antisense 24–29 nt reads mapped to Wuhan insect virus 23 show a clear preference for 1U (1 T in the cDNA sequencing), as do those mapped to Marma virus (Supplementary Fig. S8). Although the coverage plot for Marma virus shows that there are few of these antisense 24–29 nt reads against it compared to sense reads (Supplementary Fig. S3), the antisense reads may represent a small number of antiviral piRNAs overshadowed by the many reads deriving from the virus genome. Interestingly, several viruses related to families known to only infect plants showed clear 21-nt peaks and antisense 21-nt reads across the genome, suggesting they are generating siRNA responses in the mosquitoes. These viruses include *Culex*-associated luteo-like virus, *Culex*-associated tombus-like virus, *Culex*-originated *Tymoviridae*-like virus, Guadeloupe *Culex* tymo-like virus, and Marma virus (family *Luteoviridae*). Overall, despite clear evidence of piRNA ping-pong generation against four viruses and strong evidence for piRNAs without ping-pong generation for at least four others, the most common feature among the viruses was the detection of the siRNA response pathway. For 43 out of 54 examined viruses (79.6%), 21-nt was the most common mapped small RNA length for sense-mapped reads, antisense-mapped reads, or both, even when accounting for the standard error range for viruses found in multiple samples (Supplementary Fig. S3).

## Discussion

Mosquitoes are exposed to many pathogens in the field, including many that can be transmitted and are pathogenic to humans, animals, and plants, which they combat in large part by small RNA responses. By using total small RNA sequencing, we detected and characterized patterns of viral infection and improved our understanding of immune response to pathogen infection in the field.

Although our mosquitoes were caught in one general geographic area, the Inland Empire region of southern California, we detected a wide array of viruses in the samples analyzed. While these mosquitoes transmit several human pathogens, many of the detected viruses in this study have yet to be assigned to a family and demonstrate that much of the virosphere in these mosquitoes remains to be fully characterized. Using deep sequencing of viral nucleic acids, Sadeghi et al., explored the virome of over 12 thousand *Culex* mosquitoes in California^36^ and detected 56 *Culex*-associated viral strains. While the number of detected viruses between Sadeghi et al., and the present study is different, most likely due to the methodologies and the restricted geographical area use in the studies, both datasets reflect the diversity of viruses present in mosquitoes and a particular abundance of viruses with single-stranded RNA genomes. A previous study done in western Australia demonstrated that *Culex* mosquitoes possess a more diverse range of virus infection than *Aedes* mosquitoes, with 2 to 6 high-abundance viruses found in *Culex* samples and only 0 to 1 in *Aedes* samples^37^. Although we did not test *Aedes* mosquitoes, the high diversity of viruses found in our samples agrees with this claim and highlights the extended geographic range of our detected viruses, which were also found in Australia, China, and California.

Because gravid females were included in the study, it is also possible that we detected viruses associated with the blood meal rather than the mosquito. However, this is more likely for the viruses detected by blastx or with a weak small RNA signal rather than those with detected strong siRNA signature and other reads mapping to the genome, as discussed below (see Supplementary Table S2 for additional information regarding viruses detected with strong siRNA/piRNA signals). Finally, the lower-identity matches, especially those detected by blastx (Supplementary Table S3), could represent novel viruses or strains that are related to reference genomes present in the databases. The contigs that did not match any sequence could represent novel viruses and will deserve to be further investigated.

Our deep RNA-sequencing strategy not only allowed us to detect virus infection, but also mosquito small RNAs including miRNAs that map in antisense orientation to the 3′ UTRs of coding genes and could provide candidate genes that are differentially regulated between highly and lowly infected samples. Several miRNAs that have previously been associated with viral infection in mosquitoes are present in our list of upregulated miRNAs associated with high viral infection. For example, studies on WNV infection in *Culex* mosquitoes have demonstrated that miR-989 and miR-92 both can significantly alter gene expression in WNV-infected mosquitoes, and that miR-989 is downregulated upon infection with WNV while miR-92 is upregulated^51^. In *Aedes*, miR-375 was described as key to dengue virus replication^52^ and miR-252 was shown to target the dengue envelope protein gene to regulate its expression in *Ae. Albopictus* C6/36 cells^53^. Additional experiments demonstrated that introduction of miR-184 and/or miR-275 inhibits dengue virus replication^54^, while miR-281 seems to enhance replication^55^. Finally, miR-87 may contribute to the *Aedes* immune response against dengue^56^. Others of these upregulated miRNAs have been associated with non-viral pathogens such as *Wolbachia* in *Aedes* mosquitoes or *Plasmodium* malaria parasites in *Anopheles* mosquitoes. These include bantam, miR-306, miR-305, miR-317, miR-1891, miR-210, and miR-1175^49,57,58^. The remaining 15 of 29 identified miRNAs will need to be further validated but represent novel candidates for miRNAs with a significant role in controlling viral infection.

To determine specific targets of the differentially expressed miRNAs involved in virus infection or response, we used a combination of four algorithms. While experimental validation will be required to validate some of the potential targets, GO enrichment identified genes involved in translation and innate immunity. These genes are most likely targeted to control infection and stresses induced by the detected virus. As an example, we detected the putative toll protein (CPIJ018343) as a target of miR-989. This gene was predicted by all 4 algorithms and is most likely of particular interest as toll-like receptors are key to innate immunity including antiviral immunity. For mosquito samples that were highly infected with *Wolbachia,* we detected changes in gene expression of seven unique miRNAs. Of these, miR-1889 has been shown to be downregulated in *Wolbachia*-infected *Ae. aegypti*^49^, and miR-12 was demonstrated to affect *Wolbachia* density in host cells by targeting the *MCM6* and *MCT1* genes^50^. miR-309 has not been linked to *Wolbachia* but was shown to be downregulated in *Anopheles stephensi* mosquitoes infected by *Plasmodium* parasites^59^. No miRNAs were differentially expressed due to both viral and *Wolbachia* infection. As *Wolbachia* infection in mosquitoes is currently being used as a biological agent to control the spread of some mosquito-borne disease^60,61^, understanding the exact molecular mechanism controlling virus infection in *Wolbachia* infected mosquitoes could help the design of more effective strategies to combat mosquito-borne diseases across the world.

Our designed strategy allowed us to examine small RNA patterns to investigate specific immune responses against viruses using size profiles, nucleotide bias, and/or coverage plots. While this approach has been used previously to investigate the immune response against specific viruses^38,56,62,63^, to our knowledge ours is the first study to use a such wide array of viruses in field samples. Our results confirm that the siRNA pathway is the predominant small RNA response used by *Culex* mosquitoes in the field. For some viruses, the 21-nt size profile peak was much more pronounced for antisense reads, while in others, such as Hubei chryso-like virus 1, a strong signal was detected in both sense and antisense (Fig. 4a). When the 21-nt peak is more pronounced for antisense reads, it is likely that many of the 21-nt sense reads derive from the virus genome rather than siRNA pathways. The fact that we observed clear siRNA responses against viruses that have sequence similarity with plant viruses suggests that these viruses may also replicate in the mosquito. This discovery follows what has recently been shown for narnaviruses in *Culex*. This viral family was previously thought to only infect yeast and oomycetes^64^, but the high coverage of reads and strong siRNA response that we and others^4^ detected against *Culex* narnavirus 1 suggest that this virus is replicating in the mosquito.

Interestingly, all four of the viruses with clear evidence of ping-pong piRNA response generation have negative-sense single-stranded RNA genomes, indicating that the (−)ssRNA genome itself may encourage extensive activation of this piRNA pathway in *Culex* mosquitoes. However, virus genome coverage plots suggest piRNAs may be produced against other viruses as well, without use of the ping-pong cycle (Fig. 5 and Supplementary Fig. S3, https://github.com/Sabel14/MosquitoSmallRNA_Supplemental_AndCustomScripts). *Culex* narnavirus 1 represents one example, with an antisense 1U-biased 24–29 nt peak similar to the one for *Culex* phasma-like virus. A recent study done in infected *Aedes albopictus* demonstrated that piRNAs are produced against a specific region of Chikungunya virus while siRNAs target the entire genome^65^. Our data show that Hubei mosquito 4 has a relatively high amount of antisense 24–29 nt reads with 1U bias and 10-nt overlaps with sense reads, in a peak directly outside of the coding region. By contrast, Wuhan insect virus 23 and Wuhan spider virus 10 display multiple regions of antisense 24–29 nt reads which have 1U bias, suggesting a more diffuse pattern of piRNA targeting. This pattern is more similar to those for *Culex* bunya-like virus, Turlock orthobunyavirus, and Hart park hapavirus, which generate widely targeting high-confidence piRNAs with the ping-pong signature. CxNV1, Hubei mosquito virus 4, Wuhan insect virus 23, and Wuhan spider virus 10 all have positive-sense ssRNA genomes, which seem to generate few antisense-mapped reads in general. All together, these data allow us to identify antisense piRNAs targeting some of these viruses in the absence of a clear ping-pong signature and suggest that different small RNA pathways covering different regions of the genome may be a common pattern across mosquito species against different type of viruses. Although our data suggest that piRNAs may be more common in *Culex* than previously thought, the overall scarcity of evidence for piRNAs in our data does agree with previous observations that piRNA responses occur to a wider array of viruses in *Aedes* compared to *Culex* mosquitoes^4,66^. Overall, the detection of intriguing patterns of viral infection and distinct small RNA immune response demonstrate the need to expand this type of study across different parts of the world, in a wide range of mosquitoes. Such data will allow us to generate an atlas of pathogens and the mosquito immune responses they generate to not only better understand host–pathogen interaction in field samples but to also design novel strategies against many vector-borne diseases.

## Methods

See supplementary methods for further information on all sections.

### Mosquito collection, pooling, and nucleic acid extraction

For samples collected in both the Ontario and Coachella Valley areas, mosquitoes were amassed using CO_2_ traps and gravid traps by the West Valley Mosquito and Vector Control District and Coachella Valley MVCD, respectively. Nucleic acid extraction was performed using the MagMAX Viral RNA Isolation Kit (AMB18365) and samples were deep frozen at − 75 °C or lower.

### RNA extraction and validation

TRIzol was added to nucleic acid extracts for long-term storage, and RNA was extracted from this using chloroform and isopropanol precipitation. Samples were DNase-treated, checked for quality on an agarose gel, purified using Agencourt RNAclean XP beads (Beckman Coulter #A63987), and quantified using a Nanodrop spectrophotometer.

### Library preparation and sequencing

Library preparation was performed using the NEBNext Multiplex Small RNA Library Prep Set for Illumina (NEB #E7300S/L), following the provided protocol. Size selection was performed by excising the region corresponding to small RNA on a 6% TBE PAGE gel.

### Initial read processing and viral detection

Illumina sequencing results were downloaded in FASTQ form, trimmed of adapter sequence, and, for analysis beyond viral detection, filtered to retain reads of length 18 bp or higher. Trimmed reads were run through VirusDetect, an automated pipeline designed for virus discovery using deep sequencing of small RNAs^39^. We used the default settings for maximum E-value for a hit (1e−5) and minimum percentage identity (25%) for blastn, although our analysis was mostly restricted to matches with at least 90% identity. For blastx hits, we used a cutoff of 50% percentage identity to reduce potentially inaccurate results.

### Clustering and prediction based on small RNA quantity

After depleting reads that mapped to the *Cx. quinquefasciatus genome* (CpipJ2), we mapped reads to a combined file containing all virus genomes that had been detected with high confidence and filtered for uniquely mapped reads. We converted read counts to log-transformed frequencies, used UMAP to generate a lower dimensional visualization for the virus frequency matrix, and generated and inspected Pearson correlation matrices for correlations between samples and between viruses.

### Analysis of mosquito-mapped small RNA reads and miRNA analysis

Reads from *Cx. quinquefasciatus* samples were aligned to the *Cx. quinquefasciatus* genome (CpipJ2). DESeq2^45^ was used to find differentially expressed miRNA genes between different groups of samples based on cutoffs of percentages of reads mapping to viruses (0.049% of sequenced reads) or *Wolbachia* (6.34% of Culex-unmapped reads, strain endosymbiont of *Culex quinquefasciatus* Pel strain wPip, NC_010981.1). DESeq2 corrects for differences in number of reads between samples by generation of a size factor for each sample. Putative targets of differentially expressed miRNAs were predicted using sRNAtoolbox miRNAconsTarget^67^ with 4 algorithms: Simple seed analysis, TargetSpy^68^, Miranda^69^, and PITA^70^. GO enrichment was done using Fisher’s exact test through VectorBase (https://vectorbase.org).

### Analysis of small RNA response to specific viruses

Similarly to clustering analysis, *Culex*-depleted reads were mapped to combined detected virus genomes. Small RNA size profiles and nucleotide bias plots were generated using custom Python and R scripts, and genome-wide coverage plots were made using the Integrative Genomics Viewer (IGV)^71^.

## Supplementary Information

Supplementary Information 1.

Supplementary Table S1.

Supplementary Table S2.

Supplementary Table S3.

Supplementary Table S4.

Supplementary Table S5.

Supplementary Table S6.

Supplementary Table S7.

Supplementary Table S8.

## Data Availability

The datasets generated and analyzed during the current study are available in the NCBI BioProject database under accession number PRJNA705985 (https://www.ncbi.nlm.nih.gov/bioproject/).

## Abbreviations

WNV: West Nile virus
ISV: Insect-specific virus
RNAi: RNA interference
siRNA: Small interfering RNA
RISC: RNA-induced silencing complex
piRNA: PIWI-interacting RNA
(+)ssRNA: Positive-sense single-stranded RNA
(−)ssRNA: Negative-sense single-stranded RNA
HCLV1: Hubei chryso-like virus 1
CbunLV: *Culex* Bunya-like virus
CphasLV: *Culex* Phasma-like virus
CxNV1: *Culex* Narnavirus 1
UMAP: Uniform manifold approximation and projection

## Code availability

Custom scripts used for data analysis are available at https://github.com/Sabel14/MosquitoSmallRNA_Supplemental_AndCustomScripts.
