# Supplementary material for: Small RNA sequencing of field Culex mosquitoes identifies patterns of viral infection and the mosquito immune response
Source: Sci Rep. 2023 Jun 30;13:10598. doi: 10.1038/s41598-023-37571-6 (PMC10313667; doi:10.1038/s41598-023-37571-6)
Supplement: Supplementary file 1 — Supplementary Information 1. [file 41598_2023_37571_MOESM1_ESM.pdf]

Supplementary Figures and Methods

Small RNA sequencing of field *Culex* mosquitoes identifies patterns of viral infection and the mosquito immune response.

Steven M. Abel†, Zhenchen Hong†, Desiree Williams, Sally Ireri, Michelle Q. Brown, Tianyun Su, Kim Y. Hung, Jennifer A. Henke, John P. Barton††, and Karine G. Le Roch††*

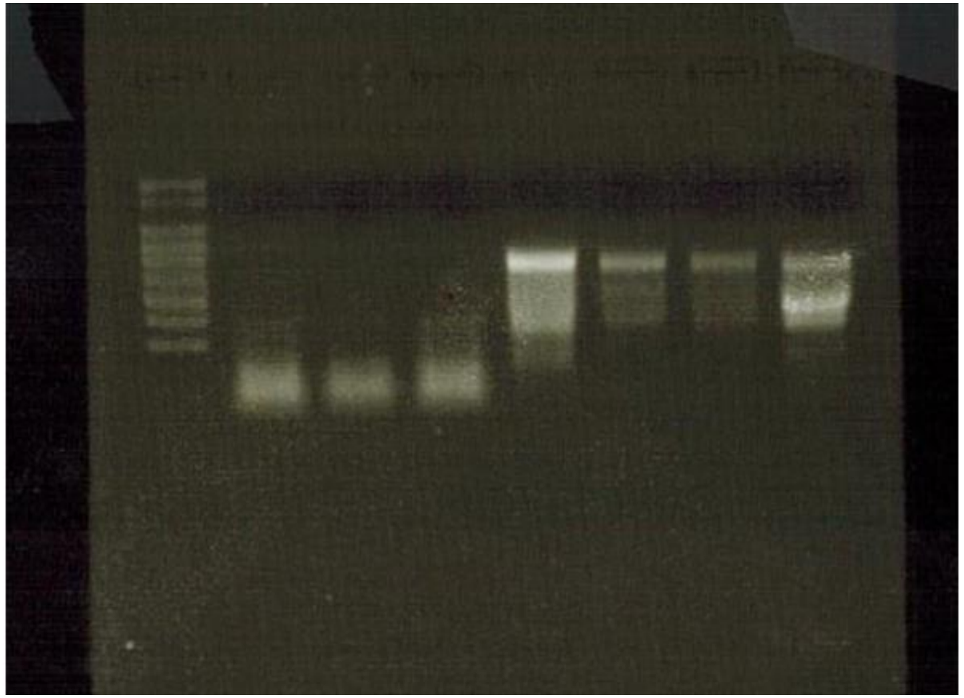

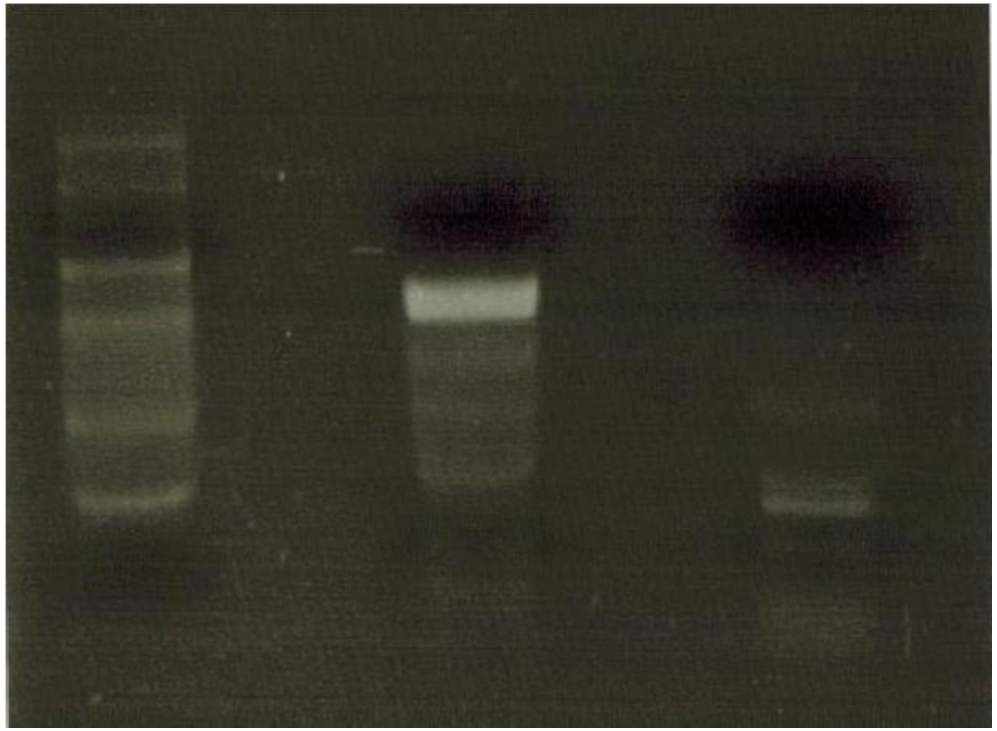

● Processed into final sample

Fig. S1: Pictures of gels showing total RNA extracted from mosquito pools.

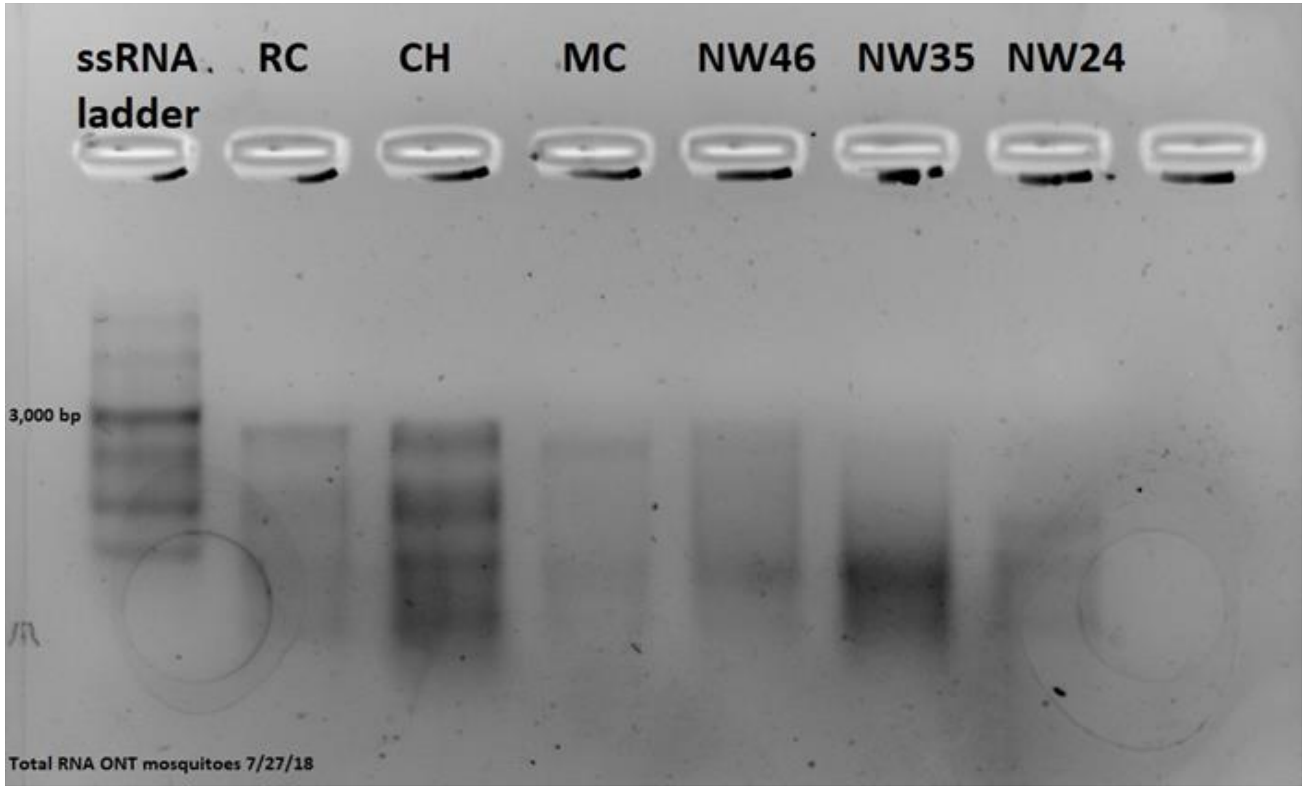

● RC ● CH ● MC ● NW46 ● NW35 ● NW24

● Processed into final sample

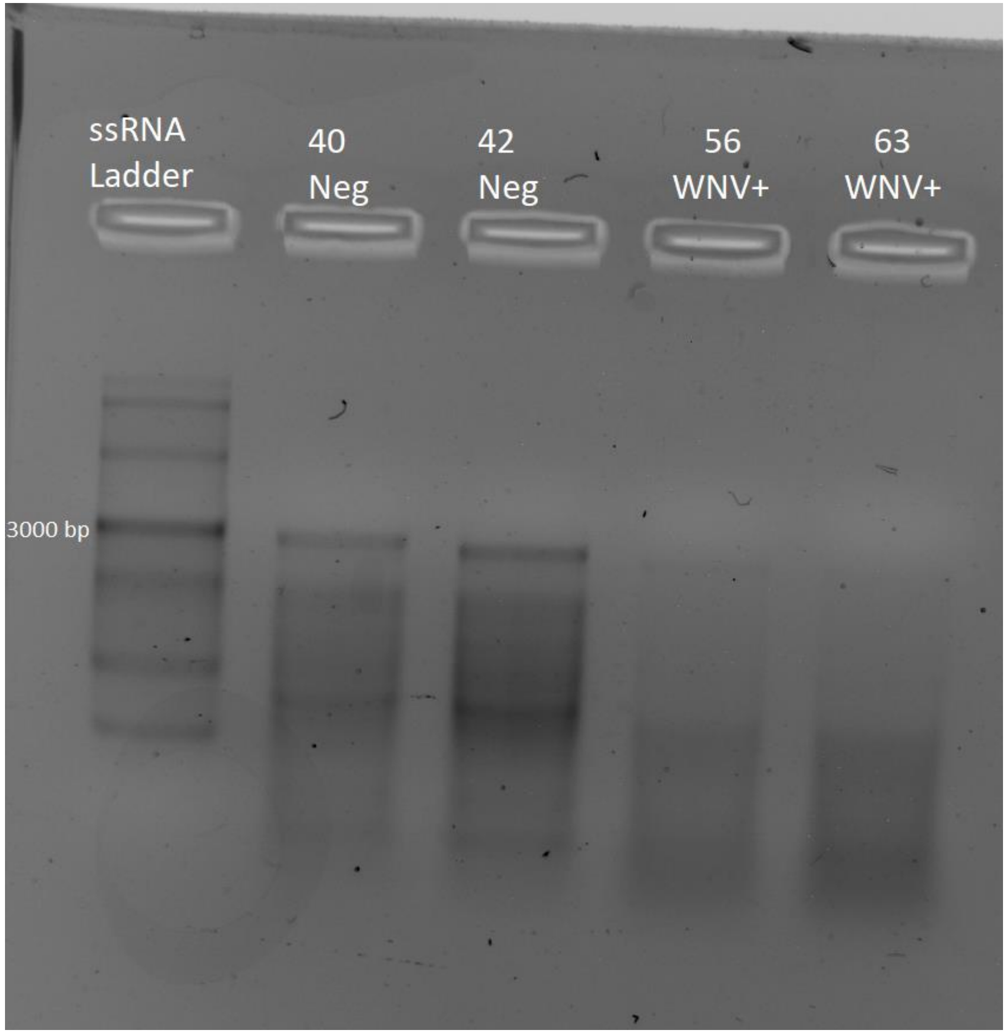

● GRLA40 ● GRLA42 ● GRLA5556 ● GRLA5563

● Processed into final sample

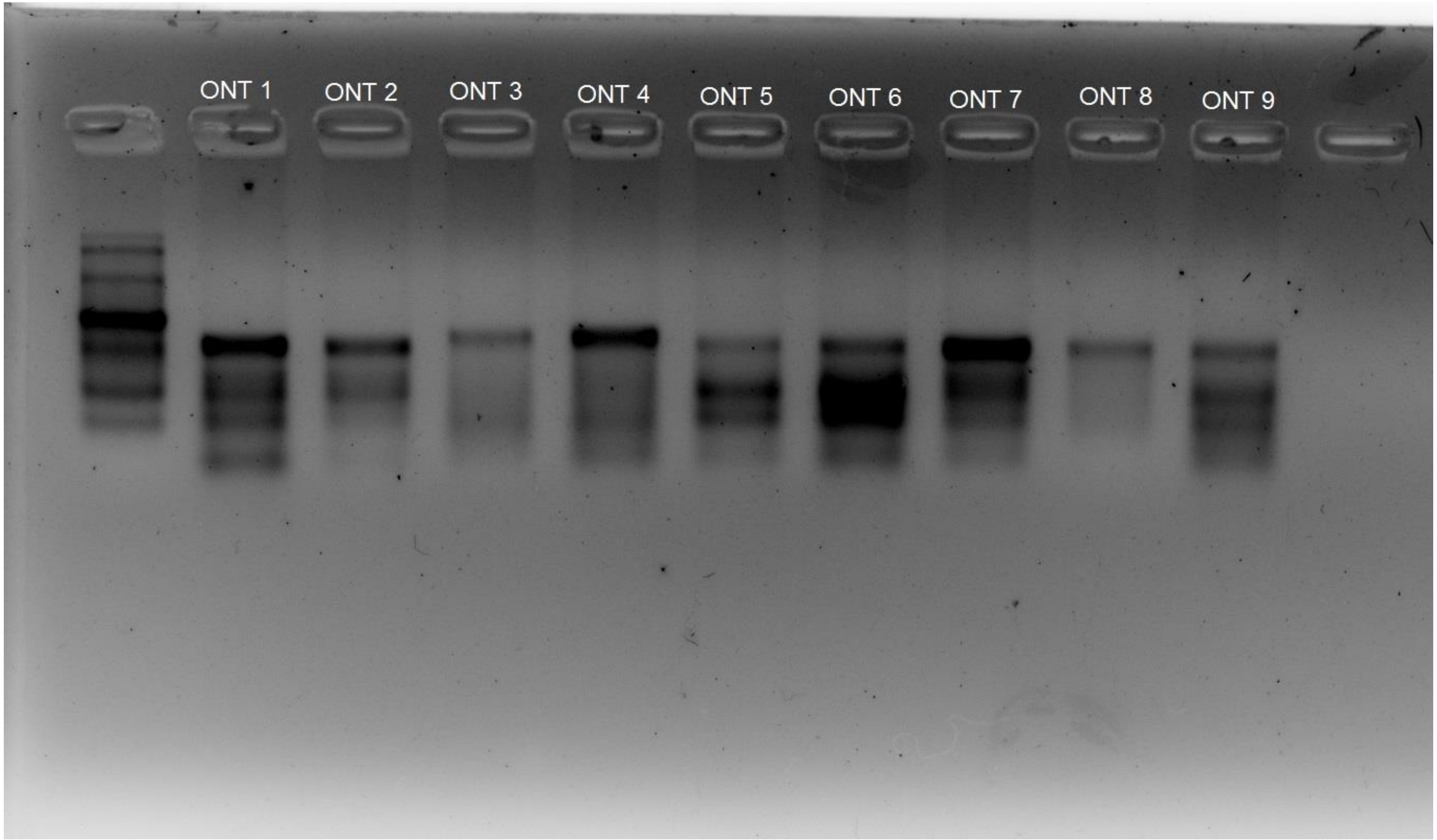

● ONT1 ● ONT2 ● ONT3 ● ONT4 ● ONT5 ● ONT6 ● ONT7 ● ONT8

● Processed into final sample

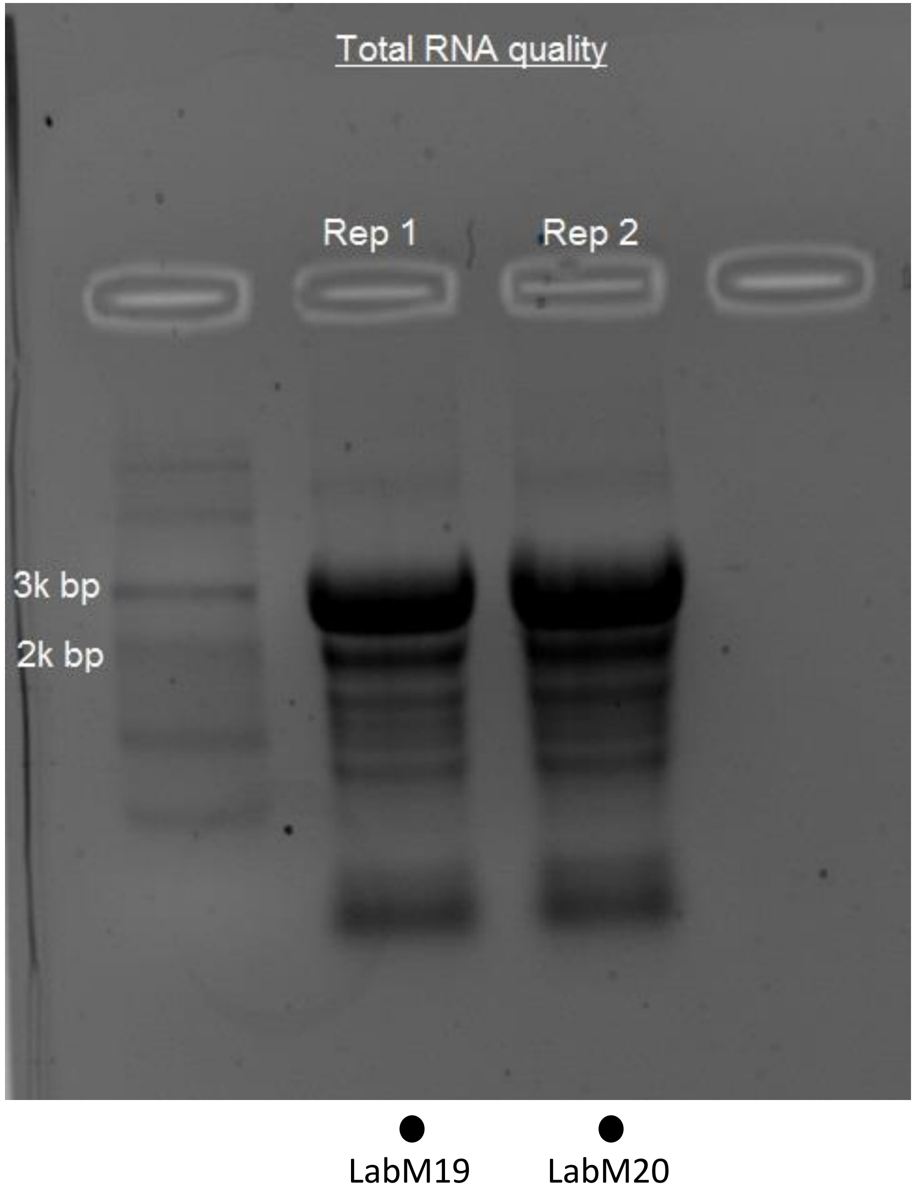

● Processed into final sample

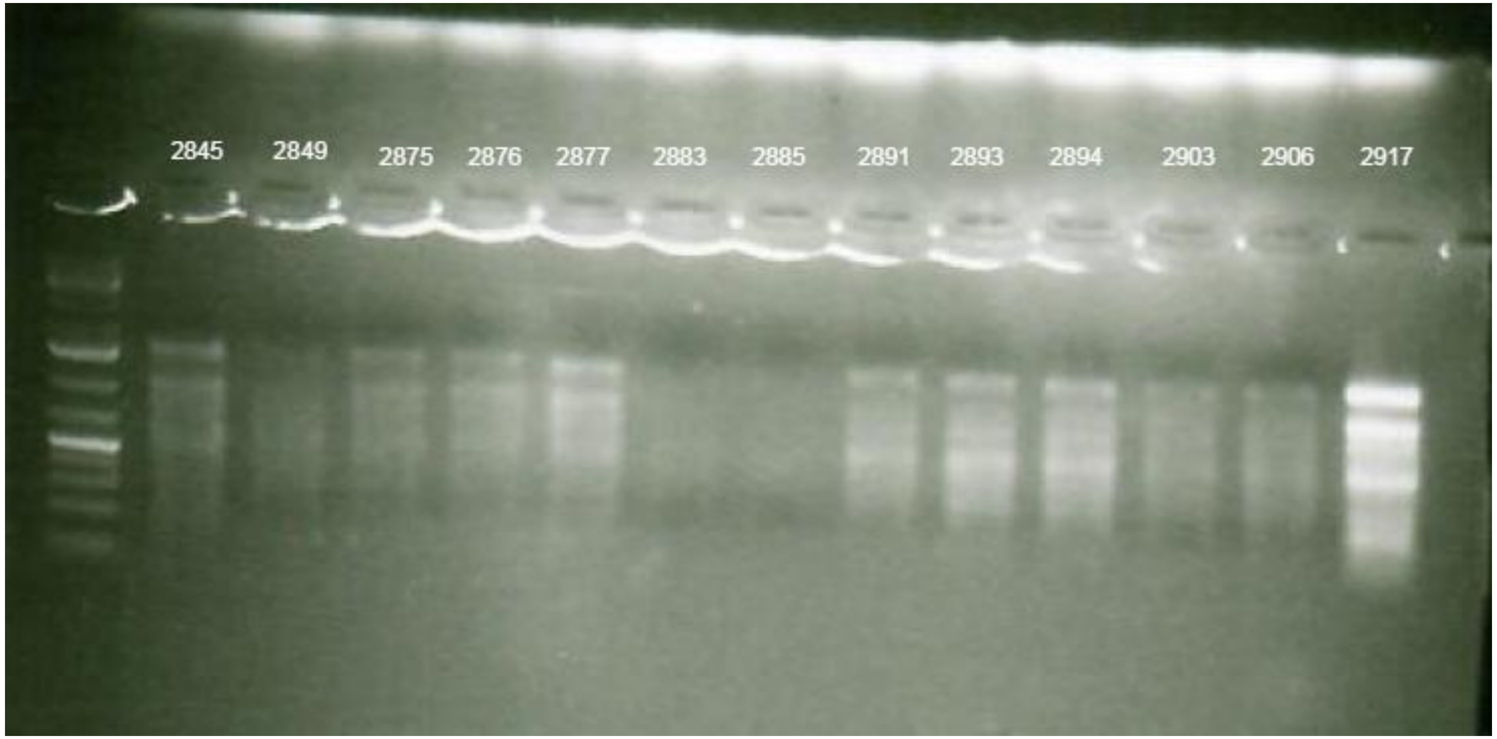

● 2845 ● 2849 ● 2875 ● 2877 ● 2885 ● 2891 ● 2893 ● 2894 ● 2906 ● 2917

● Processed into final sample

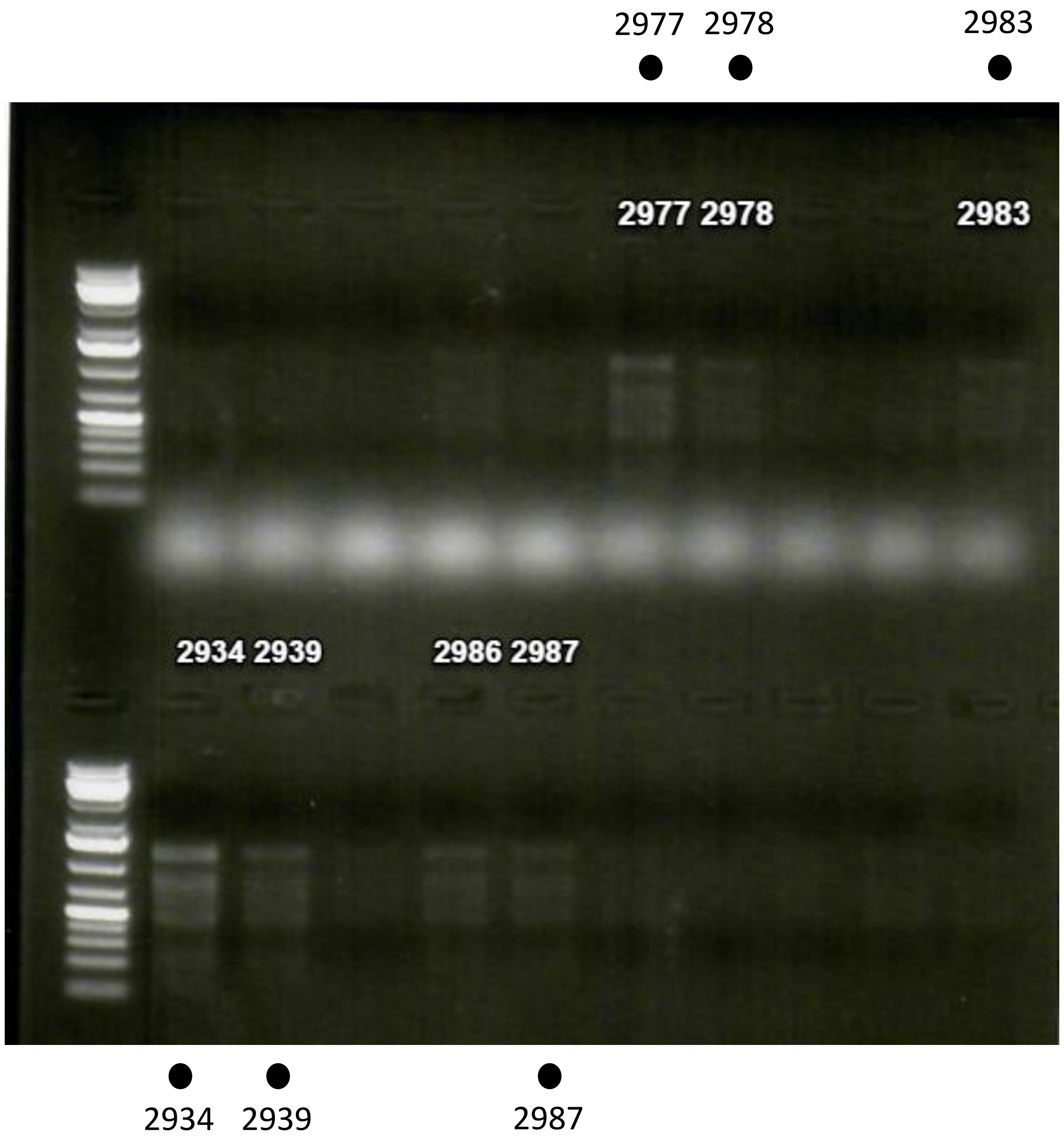

● Processed into final sample

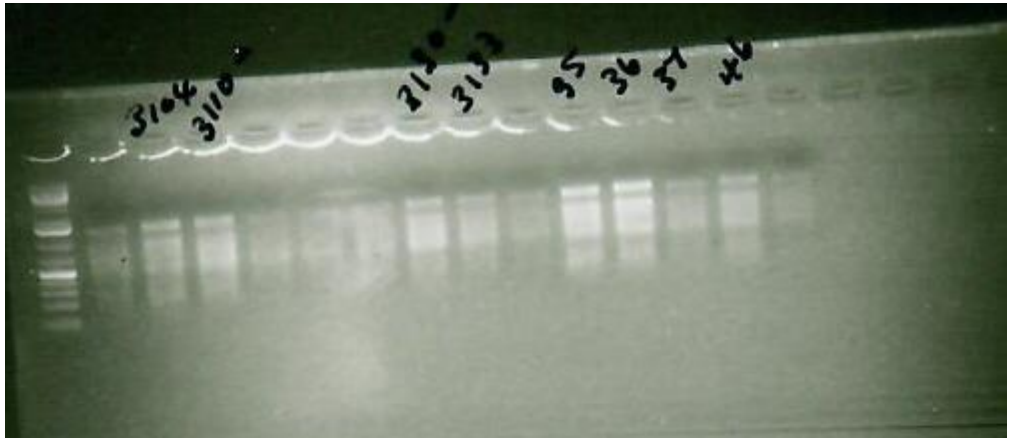

3104 3110 3130 3133 3135 3137

3136 3146

● Processed into final sample

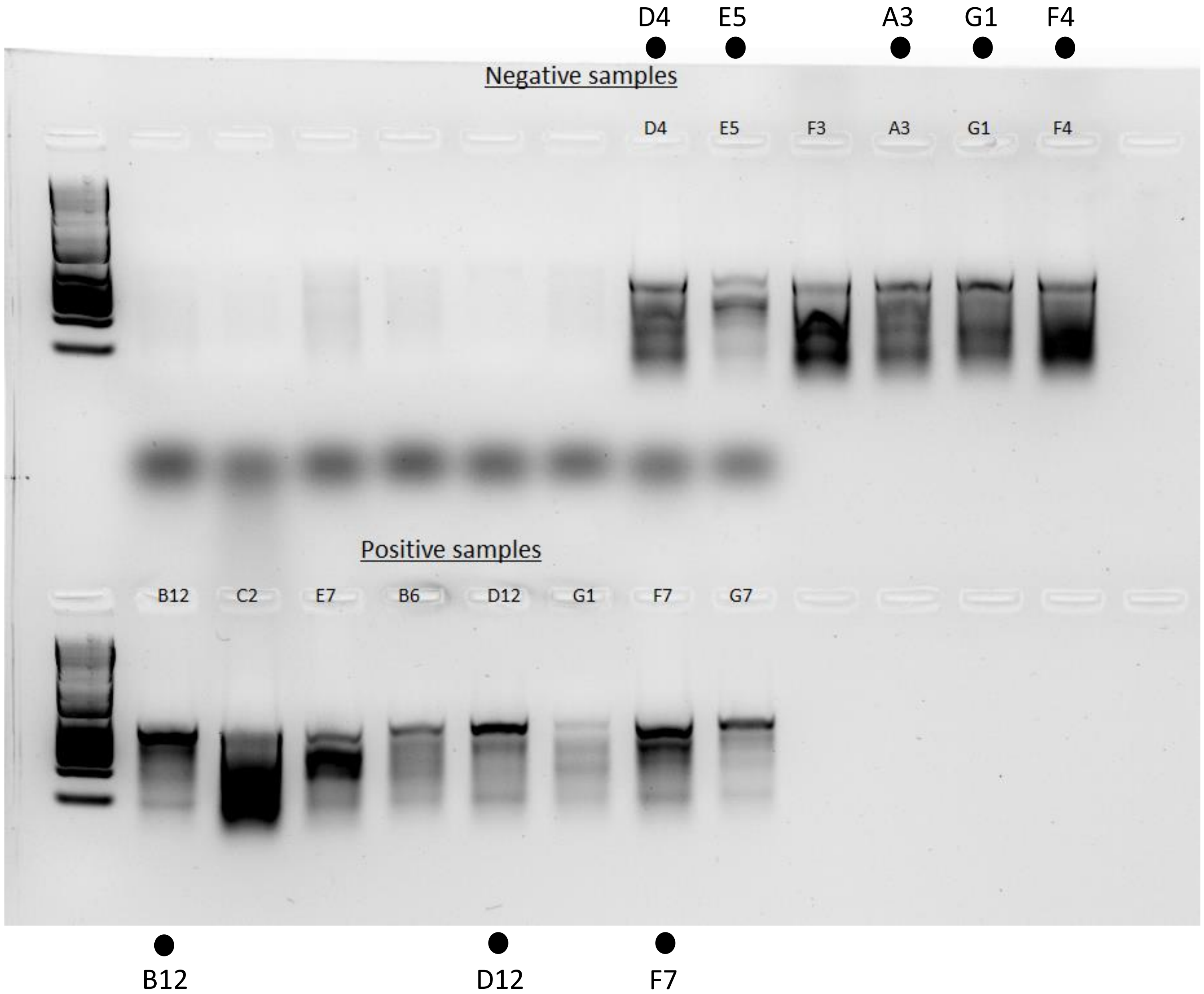

● Processed into final sample

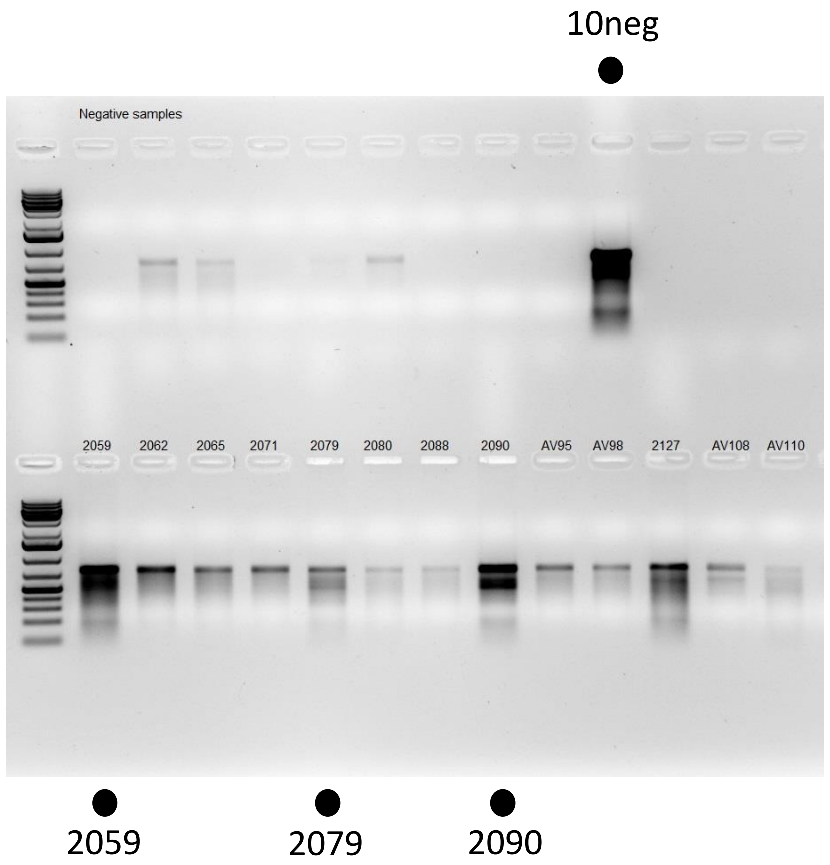

● Processed into final sample

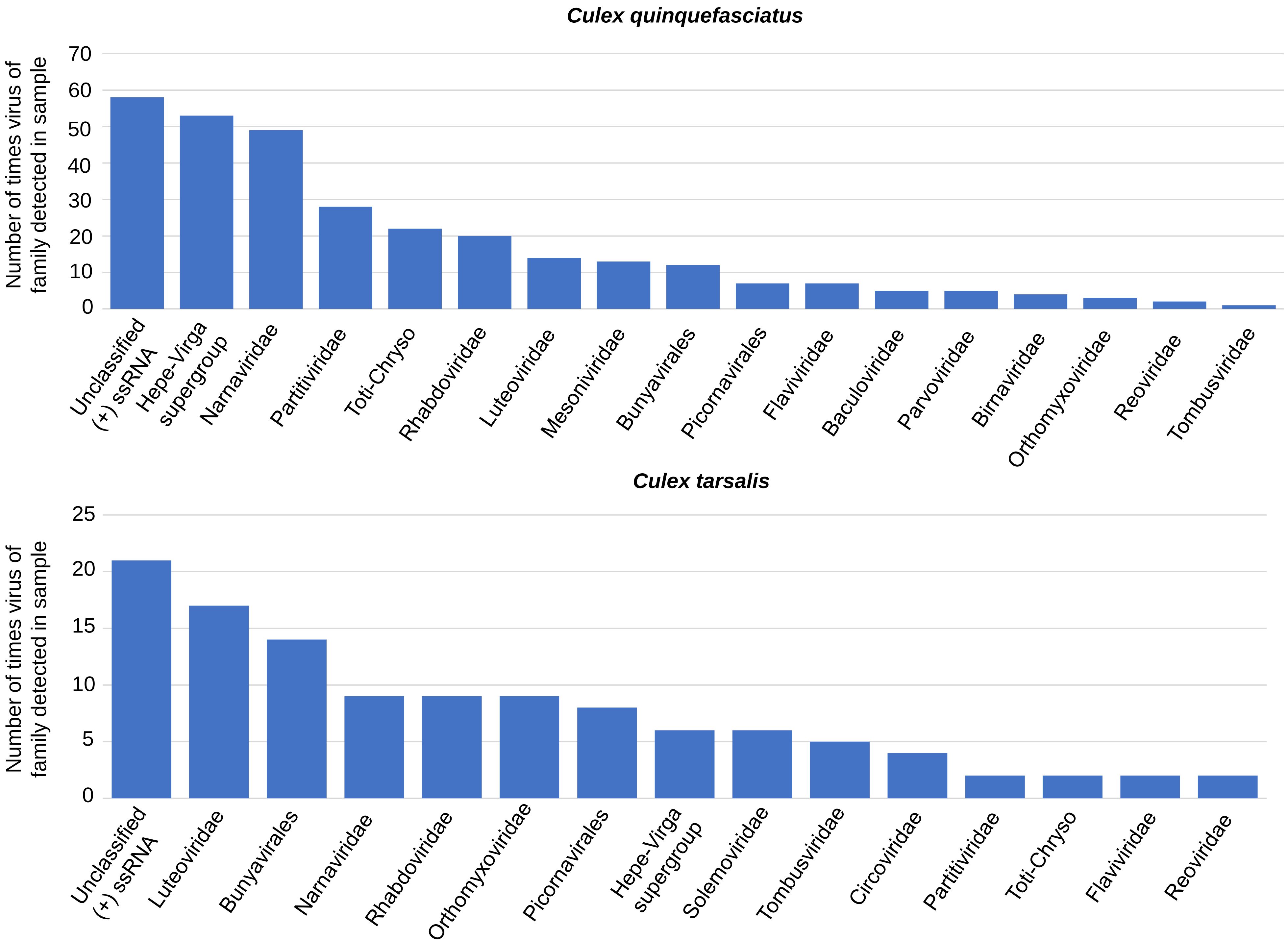

Fig. S2: Virus detections by family, separated by mosquito species.

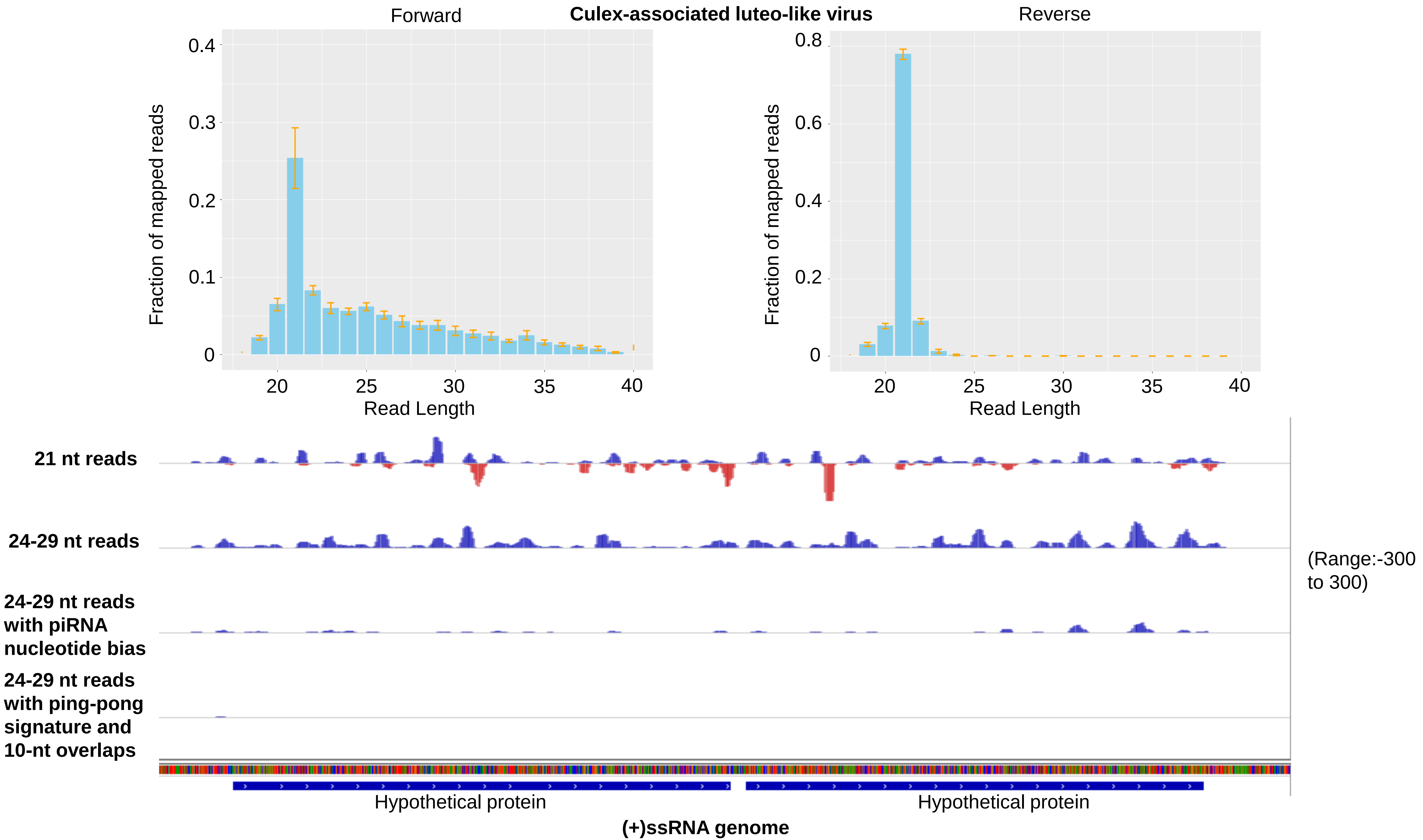

Fig. S3: Small RNA size profiles and genome coverage plots for all viruses not shown in main figures.

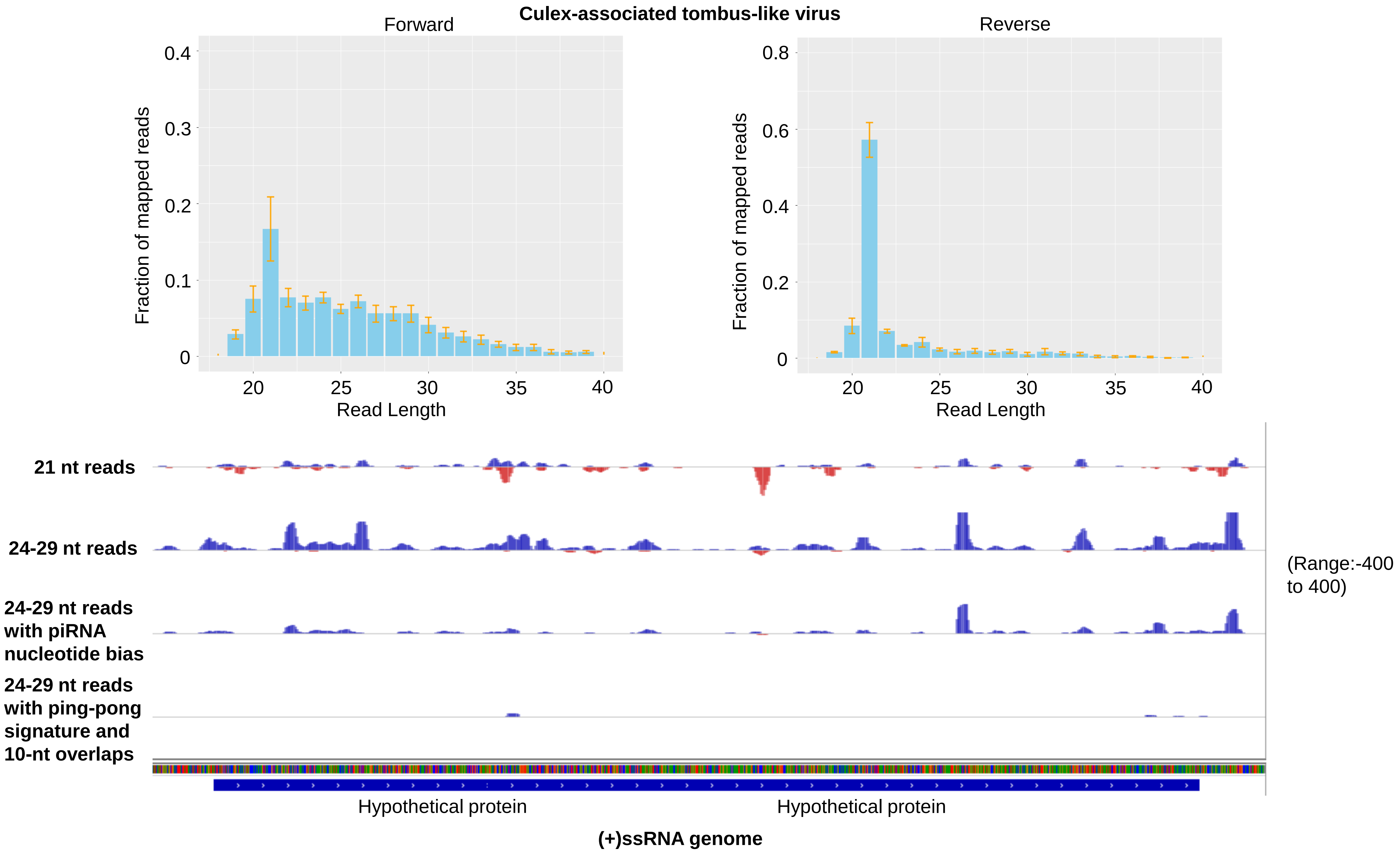

Culex-associated tombus-like virus
Forward
Reverse
Fraction of mapped reads
Read Length
0
0.1
0.2
0.3
0.4
0.6
0.8
20
25
30
35
40
21 nt reads
24-29 nt reads
24-29 nt reads with piRNA nucleotide bias
24-29 nt reads with ping-pong signature and 10-nt overlaps
(Range:-400 to 400)
Hypothetical protein
Hypothetical protein
(+)ssRNA genome

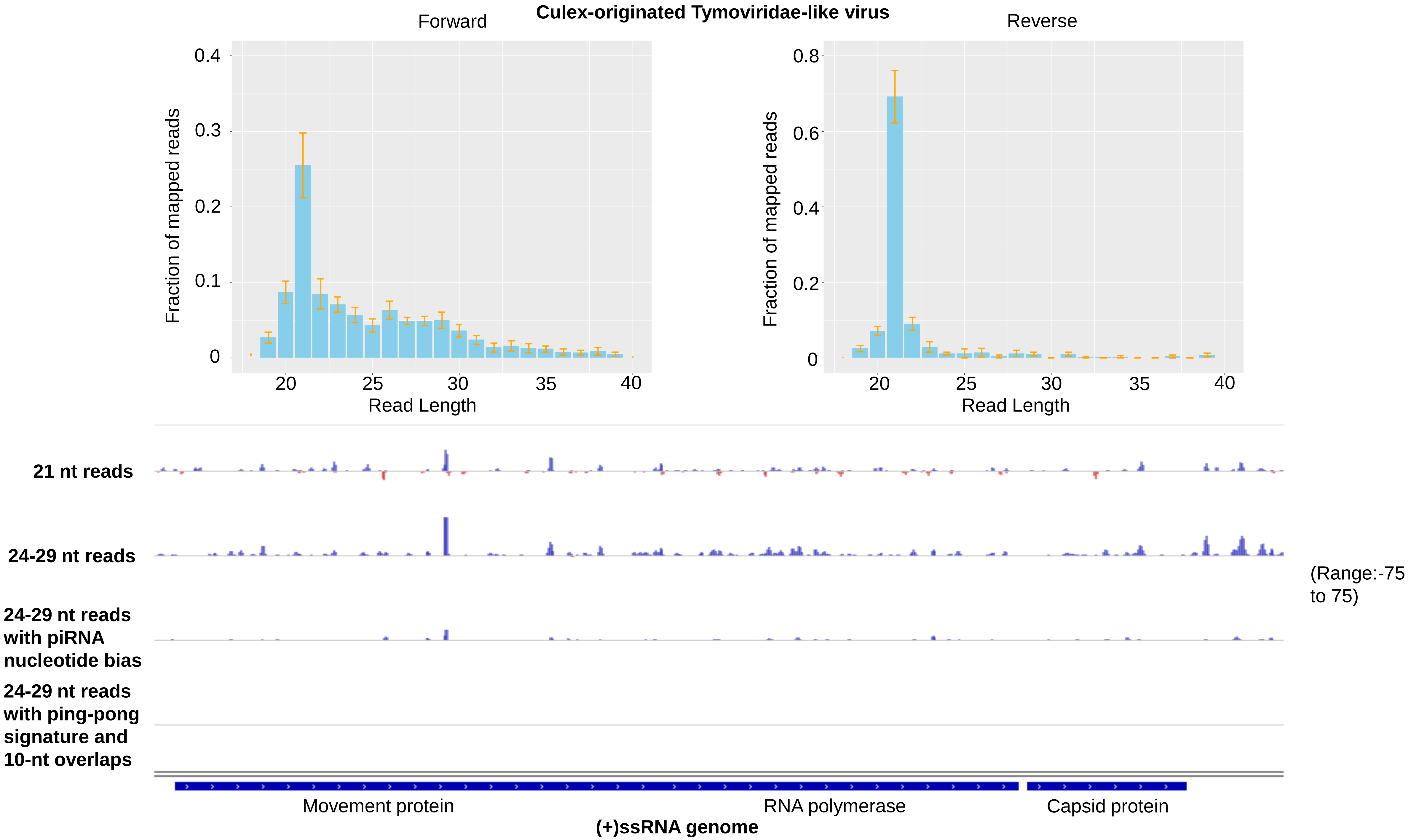

Culex-originated Tymoviridae-like virus
Forward
Reverse
Fraction of mapped reads
0.4
0.3
0.2
0.1
0
20
25
30
35
40
Read Length
0.8
0.6
0.4
0.2
0
21 nt reads
24-29 nt reads
24-29 nt reads with piRNA nucleotide bias
24-29 nt reads with ping-pong signature and 10-nt overlaps
(Range:-75 to 75)
Movement protein
RNA polymerase
Capsid protein
(+)ssRNA genome

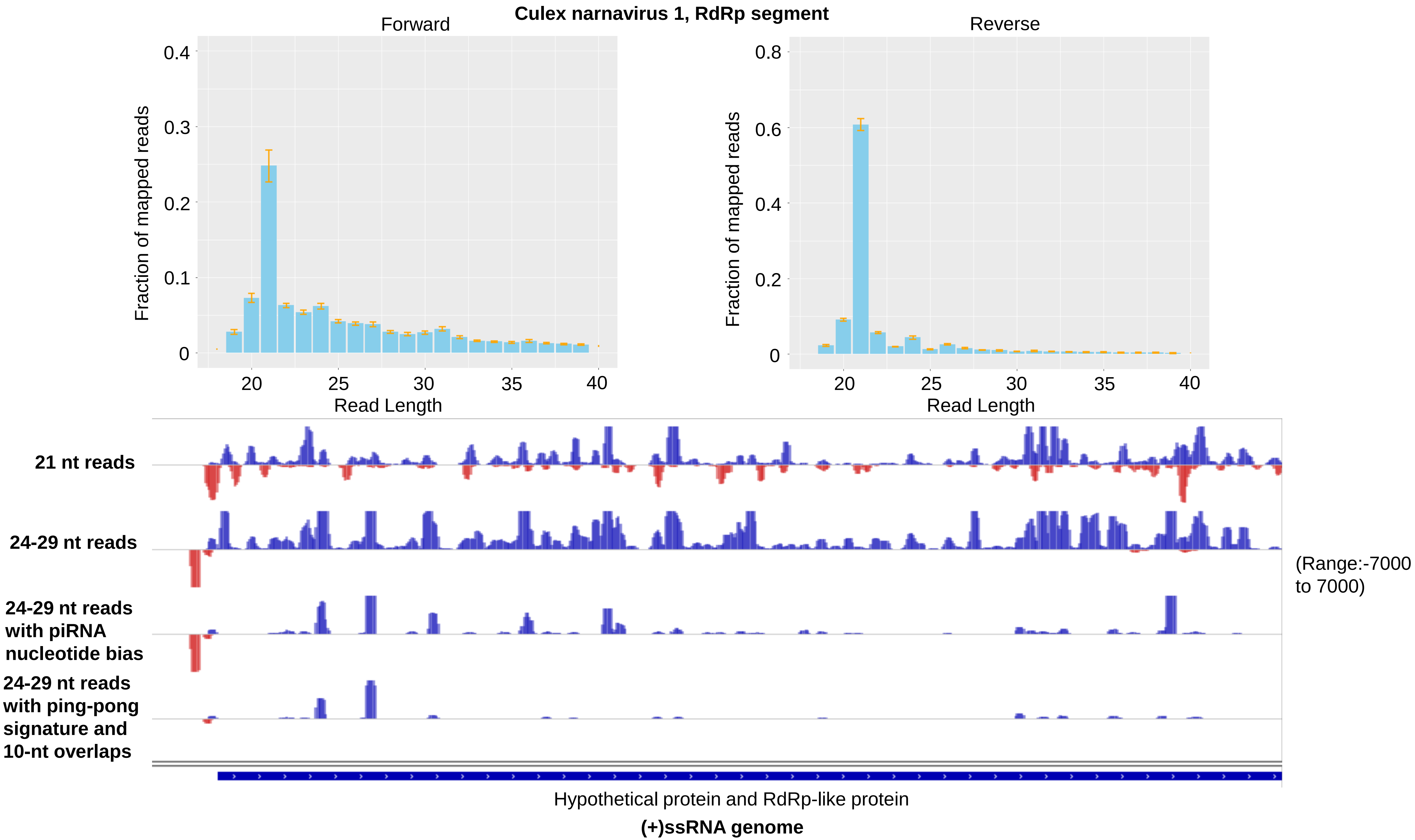
Culex narnavirus 1, RdRp segment
Forward
Reverse
Fraction of mapped reads
0
0.1
0.2
0.3
0.4
0.6
0.8
20
25
30
35
40
Read Length
21 nt reads
24-29 nt reads
24-29 nt reads with piRNA nucleotide bias
24-29 nt reads with ping-pong signature and 10-nt overlaps
(Range:-7000 to 7000)
Hypothetical protein and RdRp-like protein
(+)ssRNA genome

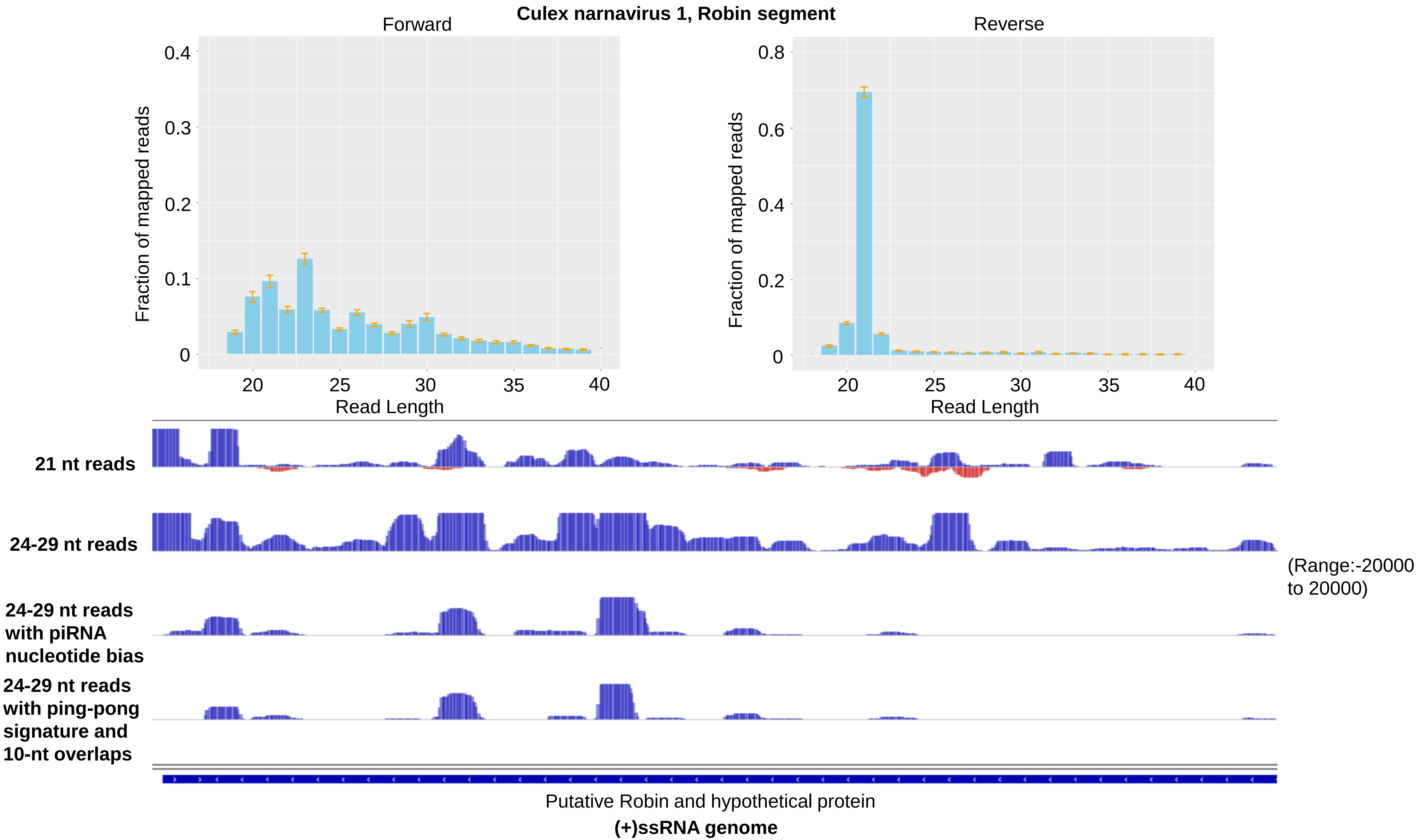

Culex narnavirus 1, Robin segment
Forward
Reverse
Fraction of mapped reads
0.4
0.3
0.2
0.1
0
0.8
0.6
0.4
0.2
0
20
25
30
35
40
Read Length
21 nt reads
24-29 nt reads
24-29 nt reads with piRNA nucleotide bias
24-29 nt reads with ping-pong signature and 10-nt overlaps
(Range:-20000 to 20000)
Putative Robin and hypothetical protein
(+)ssRNA genome

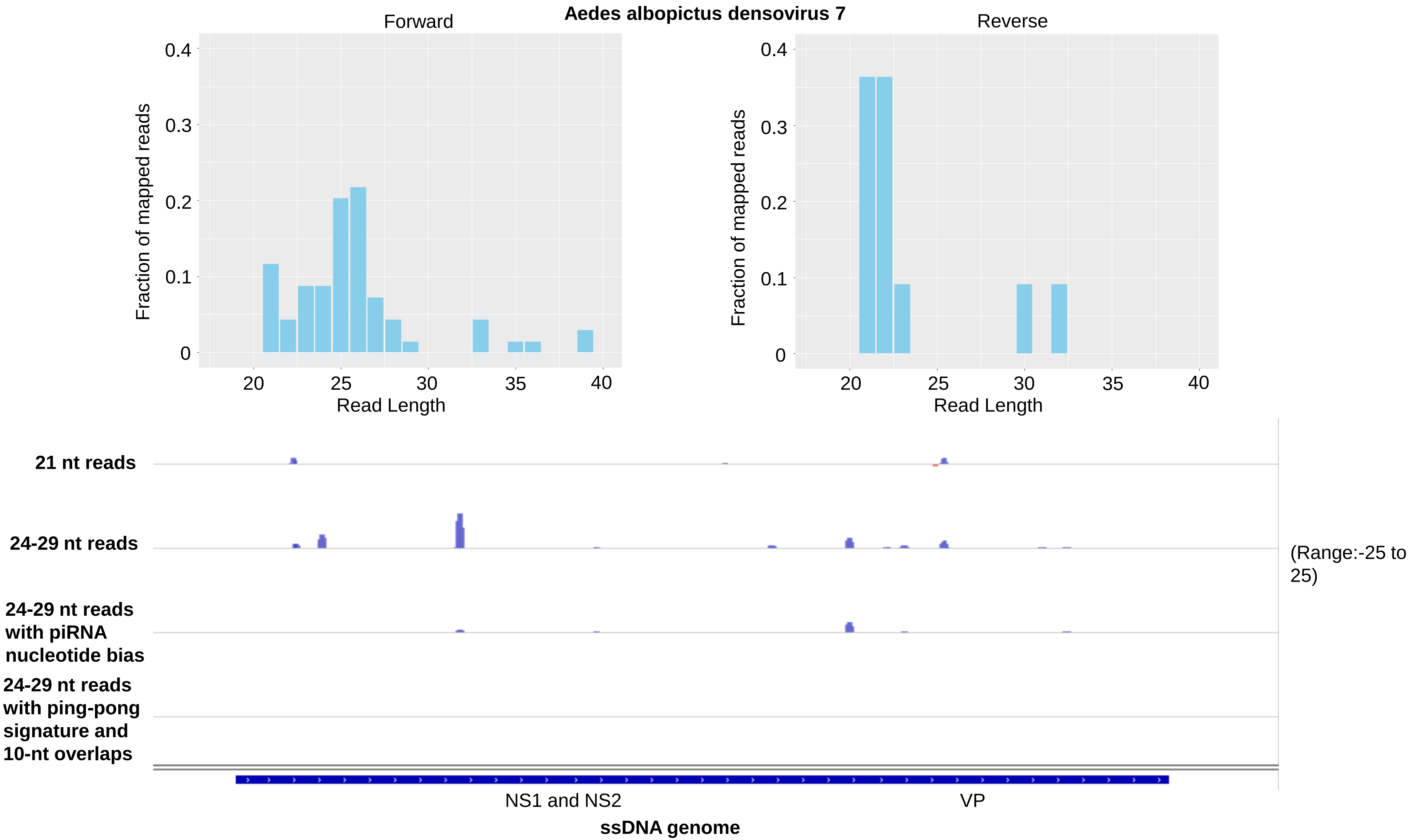

Aedes albopictus densovirus 7
Forward
Reverse
Fraction of mapped reads
0.4
0.3
0.2
0.1
0
20
25
30
35
40
Read Length
21 nt reads
24-29 nt reads
24-29 nt reads with piRNA nucleotide bias
24-29 nt reads with ping-pong signature and 10-nt overlaps
(Range:-25 to 25)
NS1 and NS2
VP
ssDNA genome

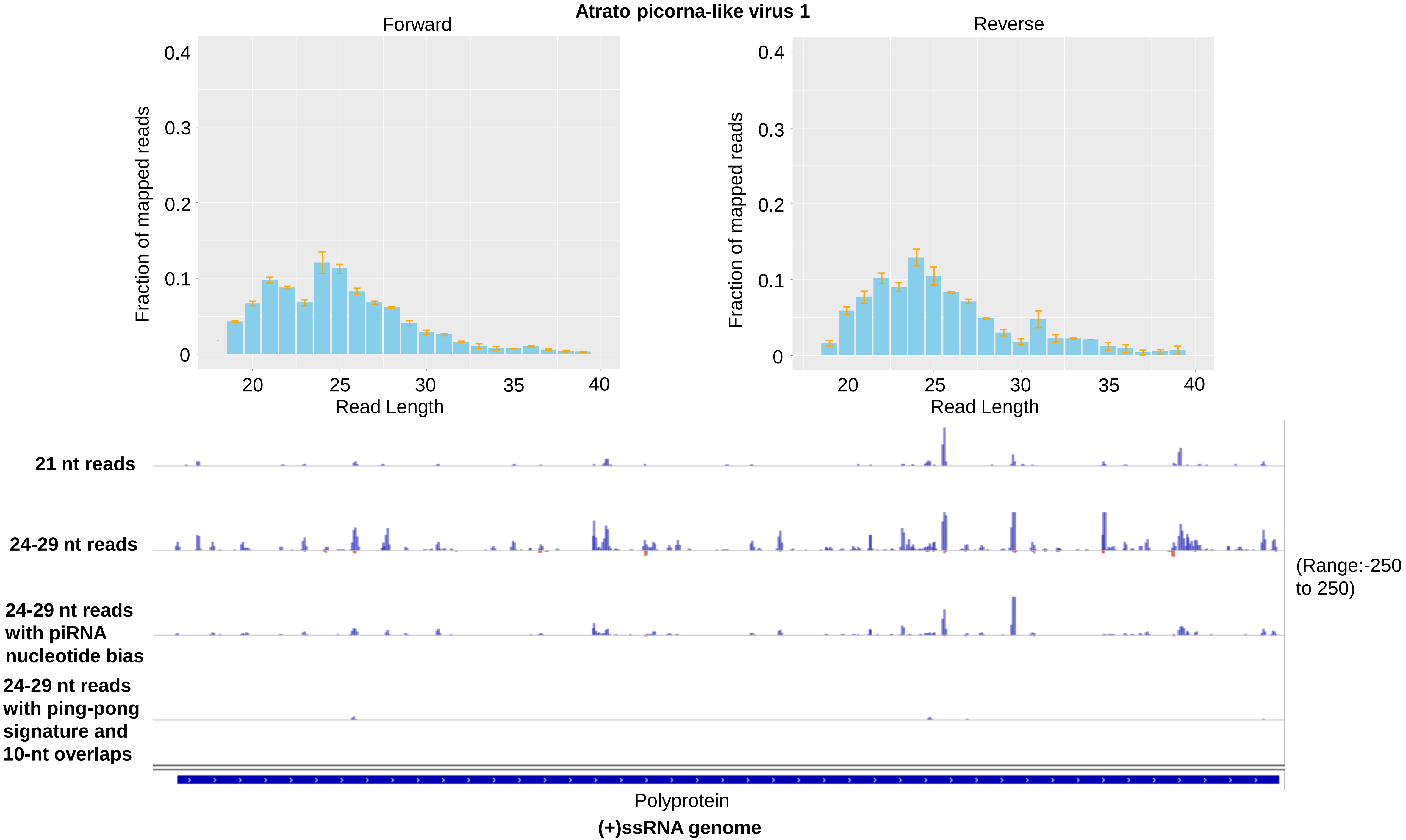

Atrato picorna-like virus 1
Forward
Reverse
Fraction of mapped reads
0.4
0.3
0.2
0.1
0
20
25
30
35
40
Read Length
21 nt reads
24-29 nt reads
24-29 nt reads with piRNA nucleotide bias
24-29 nt reads with ping-pong signature and 10-nt overlaps
(Range:-250 to 250)
Polyprotein
(+)ssRNA genome

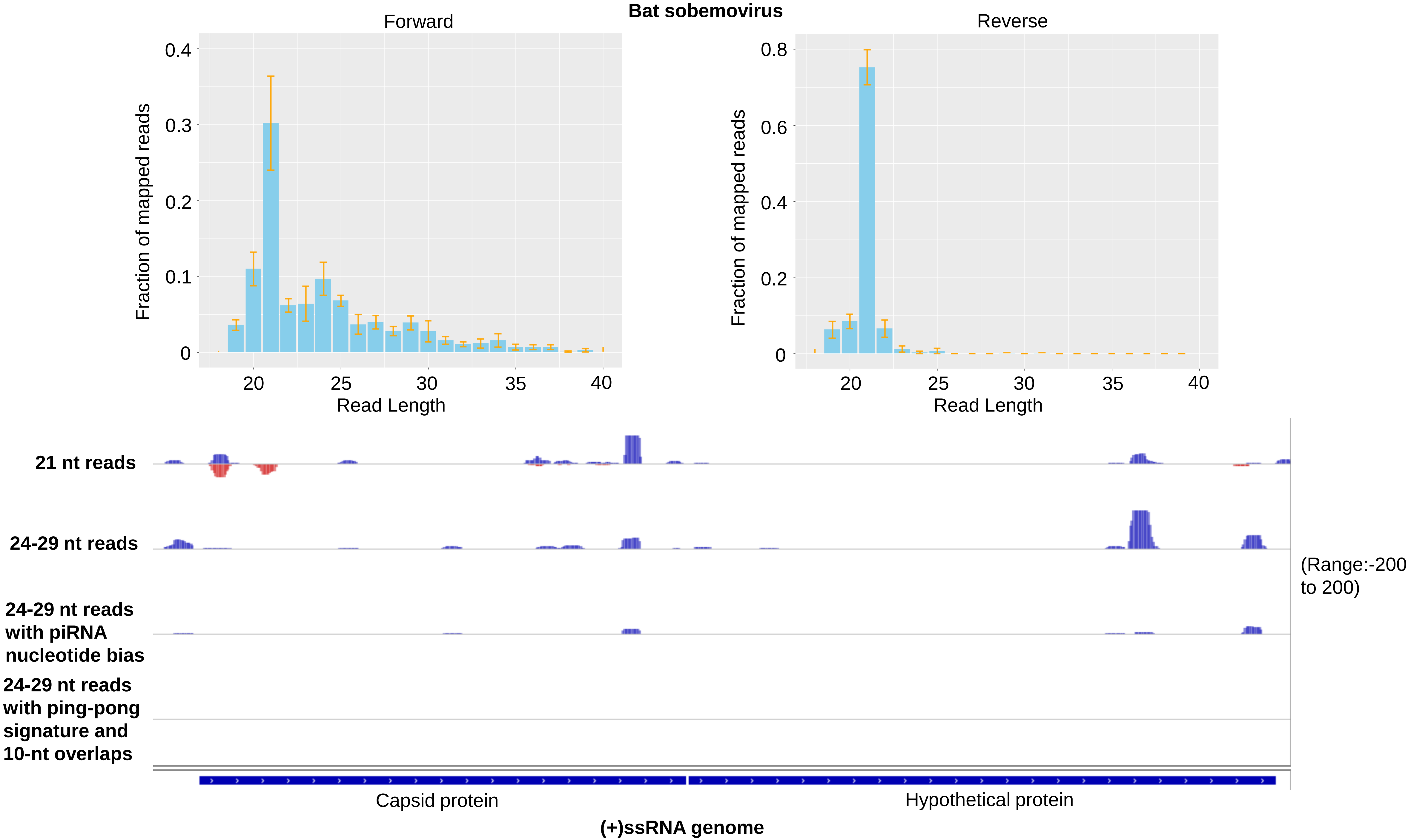

Bat sobemovirus
Forward
Reverse
Fraction of mapped reads
Read Length
0
0.1
0.2
0.3
0.4
0.6
0.8
20
25
30
35
40
21 nt reads
24-29 nt reads
24-29 nt reads with piRNA nucleotide bias
24-29 nt reads with ping-pong signature and 10-nt overlaps
(Range:-200 to 200)
Capsid protein
Hypothetical protein
(+)ssRNA genome

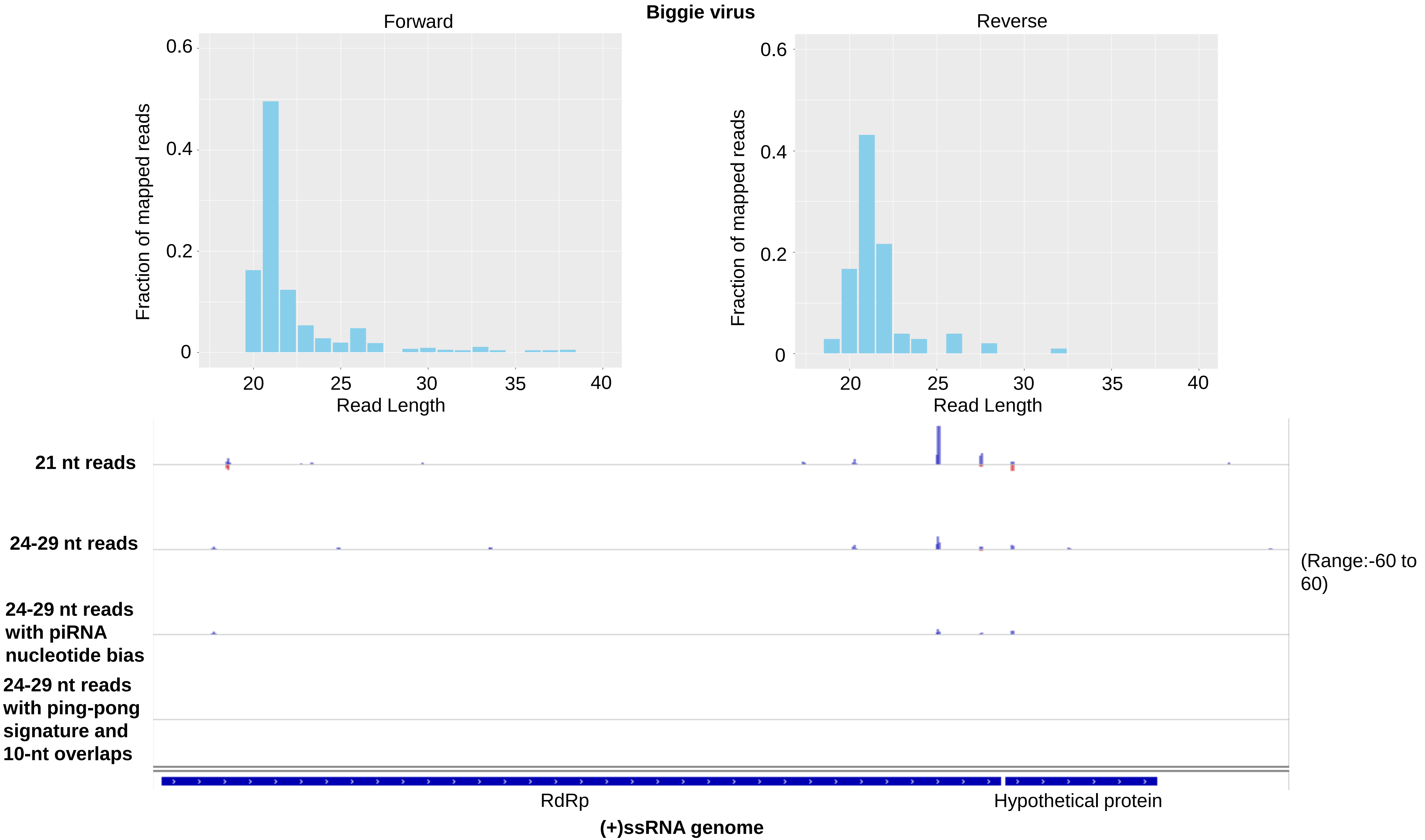
Biggie virus
Forward
Reverse
Fraction of mapped reads
0.6
0.4
0.2
0
20
25
30
35
40
Read Length
21 nt reads
24-29 nt reads
24-29 nt reads with piRNA nucleotide bias
24-29 nt reads with ping-pong signature and 10-nt overlaps
(Range:-60 to 60)
RdRp
Hypothetical protein
(+)ssRNA genome

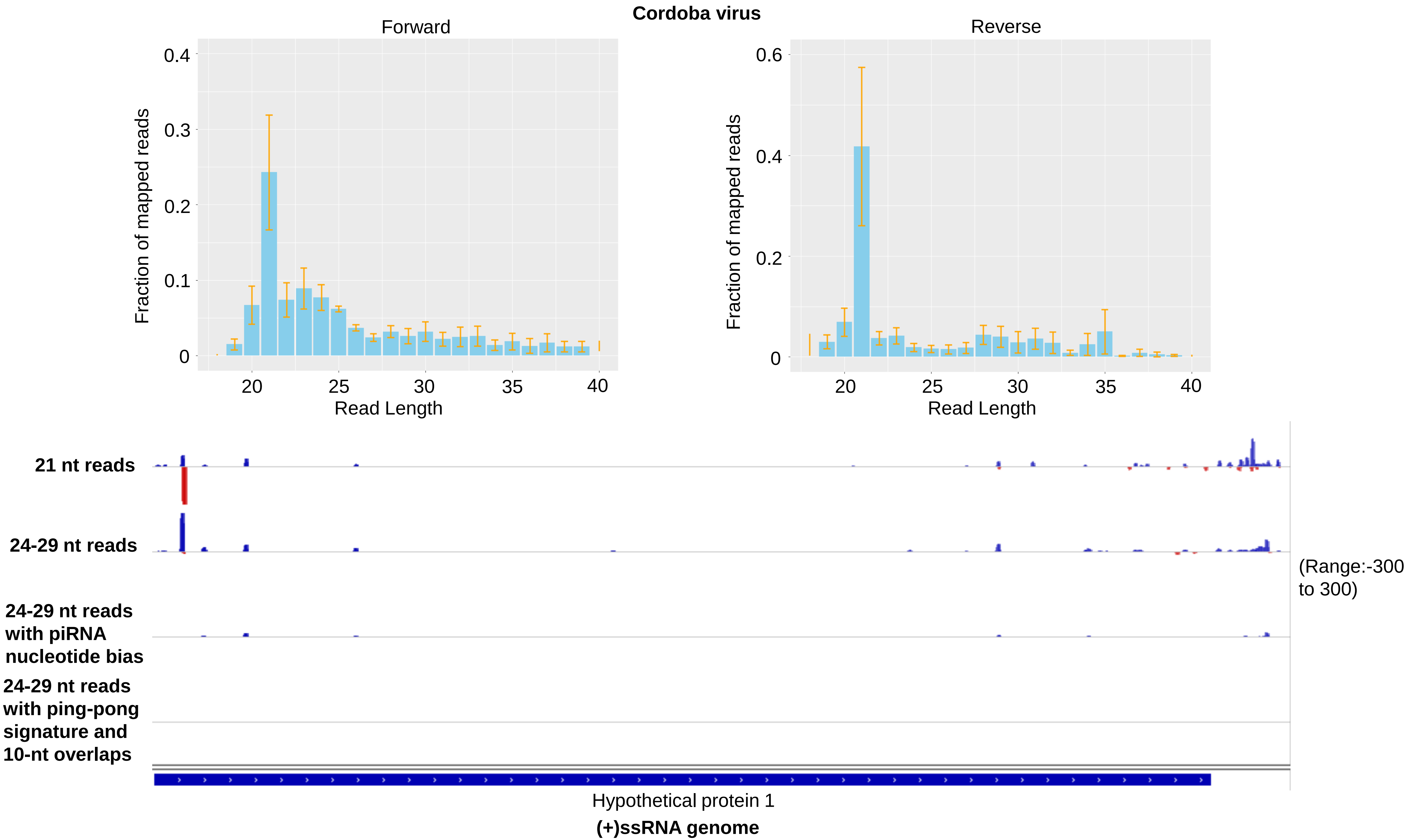
Cordoba virus
Forward
Reverse
Fraction of mapped reads
Read Length
0
0.1
0.2
0.3
0.4
0.6
20
25
30
35
40
21 nt reads
24-29 nt reads
24-29 nt reads with piRNA nucleotide bias
24-29 nt reads with ping-pong signature and 10-nt overlaps
(Range:-300 to 300)
Hypothetical protein 1
(+)ssRNA genome

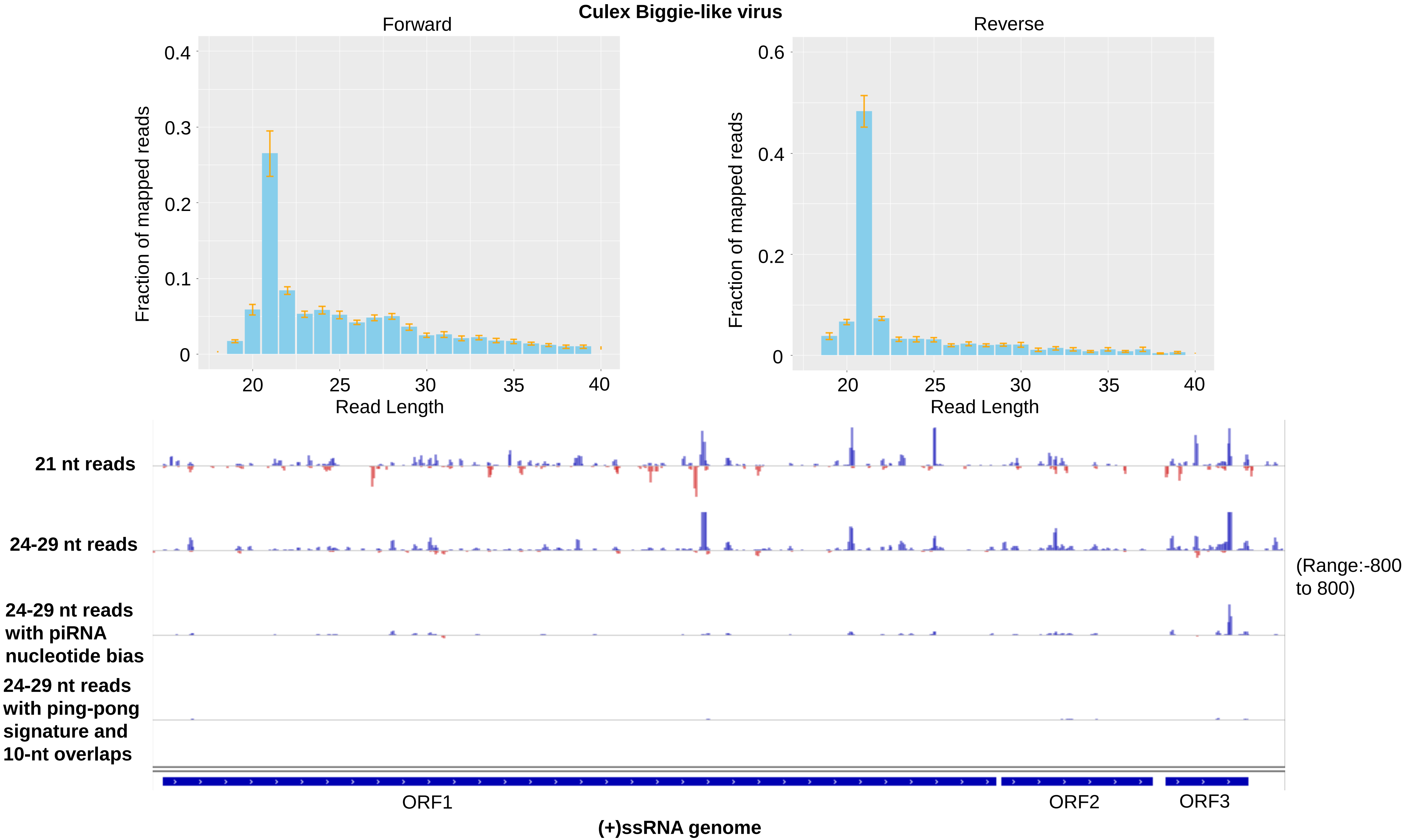
Culex Biggie-like virus
Forward
Reverse
Fraction of mapped reads
Read Length
21 nt reads
24-29 nt reads
24-29 nt reads with piRNA nucleotide bias
24-29 nt reads with ping-pong signature and 10-nt overlaps
(Range:-800 to 800)
ORF1
ORF2
ORF3
(+)ssRNA genome

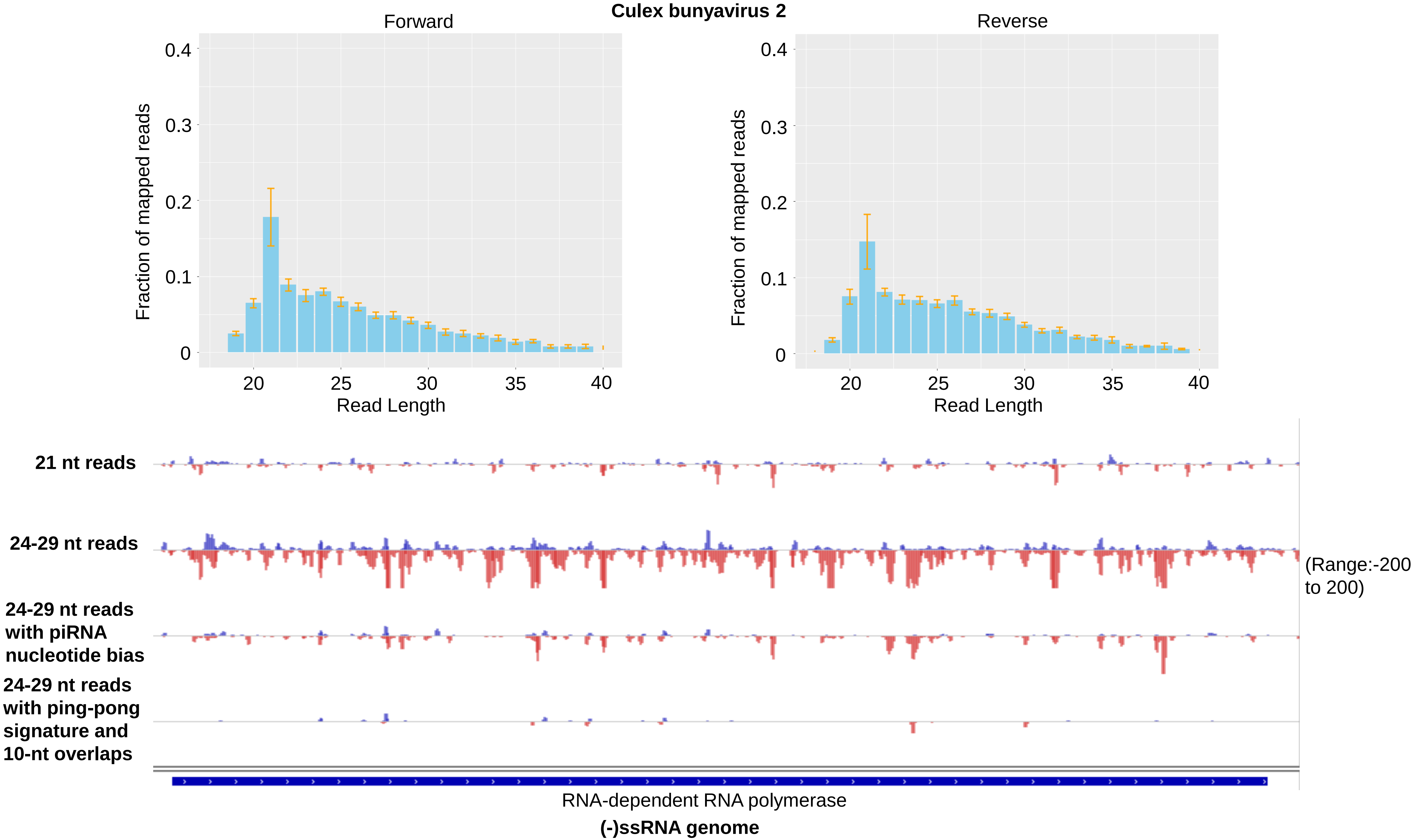
Culex bunyavirus 2
Forward
Reverse
Fraction of mapped reads
Read Length
0
0.1
0.2
0.3
0.4
20
25
30
35
40
21 nt reads
24-29 nt reads
24-29 nt reads with piRNA nucleotide bias
24-29 nt reads with ping-pong signature and 10-nt overlaps
(Range:-200 to 200)
RNA-dependent RNA polymerase
(-)ssRNA genome

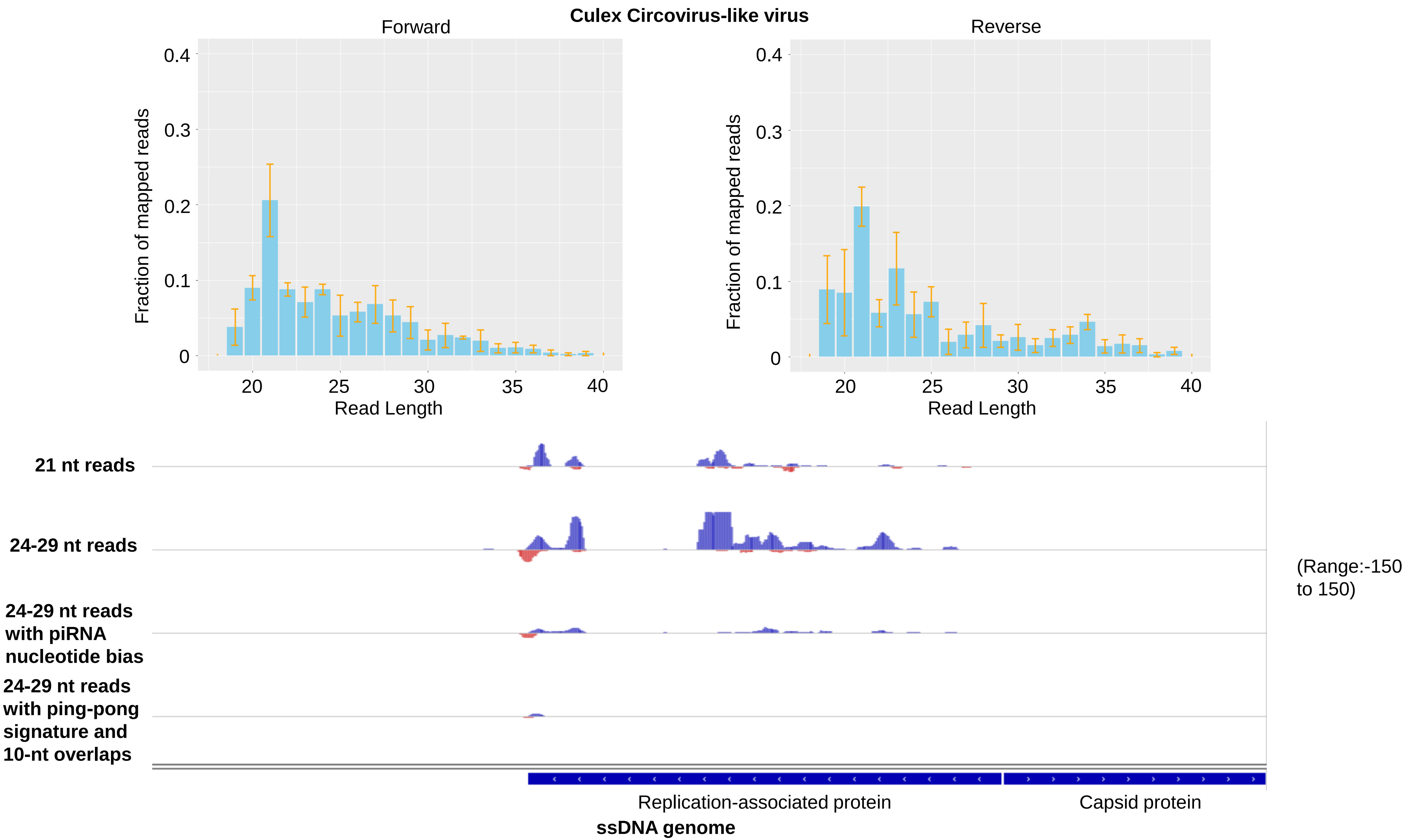
Culex Circovirus-like virus
Forward
Reverse
Fraction of mapped reads
0.4
0.3
0.2
0.1
0
20
25
30
35
40
Read Length
21 nt reads
24-29 nt reads
24-29 nt reads with piRNA nucleotide bias
24-29 nt reads with ping-pong signature and 10-nt overlaps
(Range:-150 to 150)
Replication-associated protein
Capsid protein
ssDNA genome

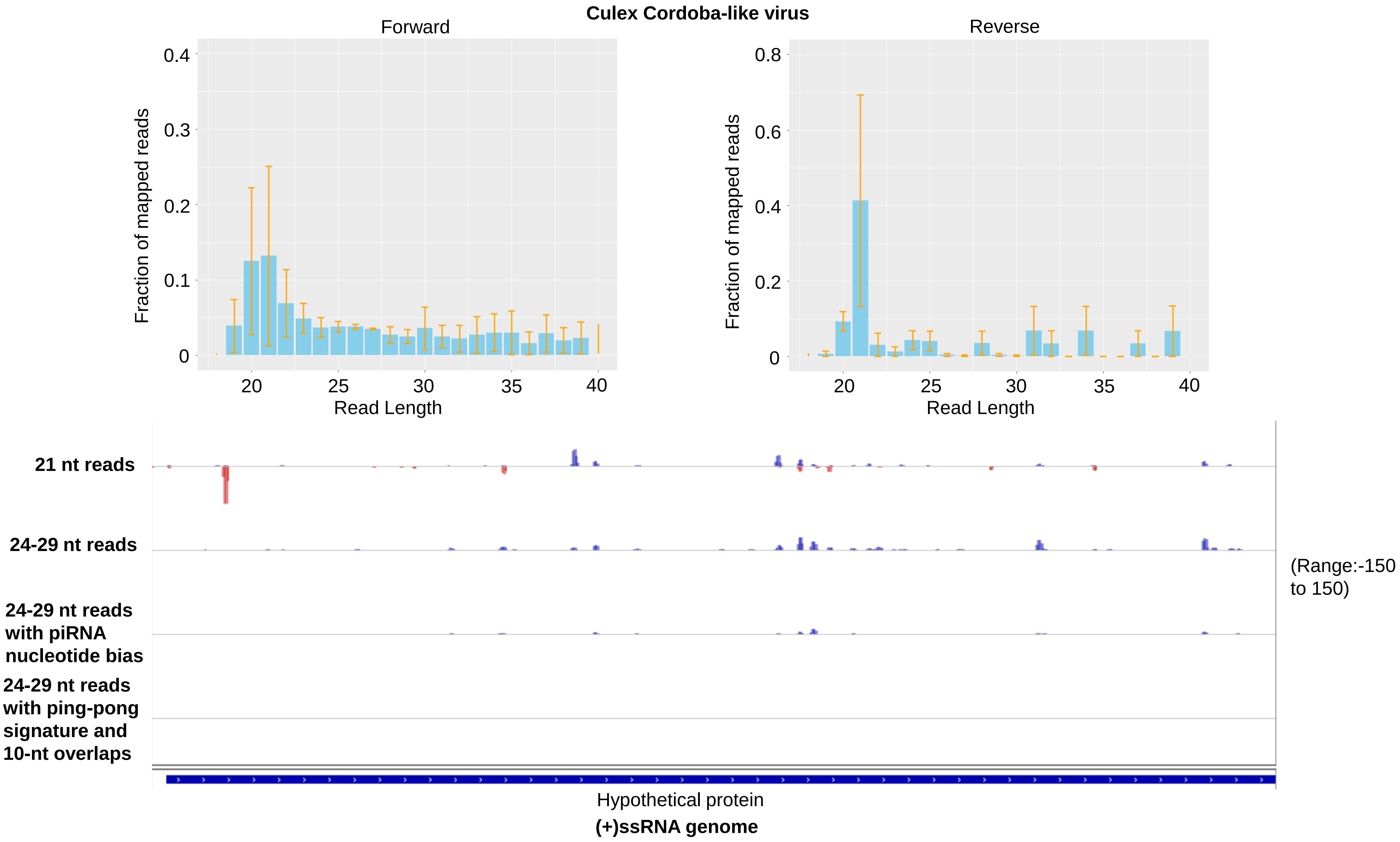

Culex Cordoba-like virus
Forward
Reverse
Fraction of mapped reads
0
0.1
0.2
0.3
0.4
0.6
0.8
20
25
30
35
40
Read Length
21 nt reads
24-29 nt reads
24-29 nt reads with piRNA nucleotide bias
24-29 nt reads with ping-pong signature and 10-nt overlaps
(Range:-150 to 150)
Hypothetical protein
(+)ssRNA genome

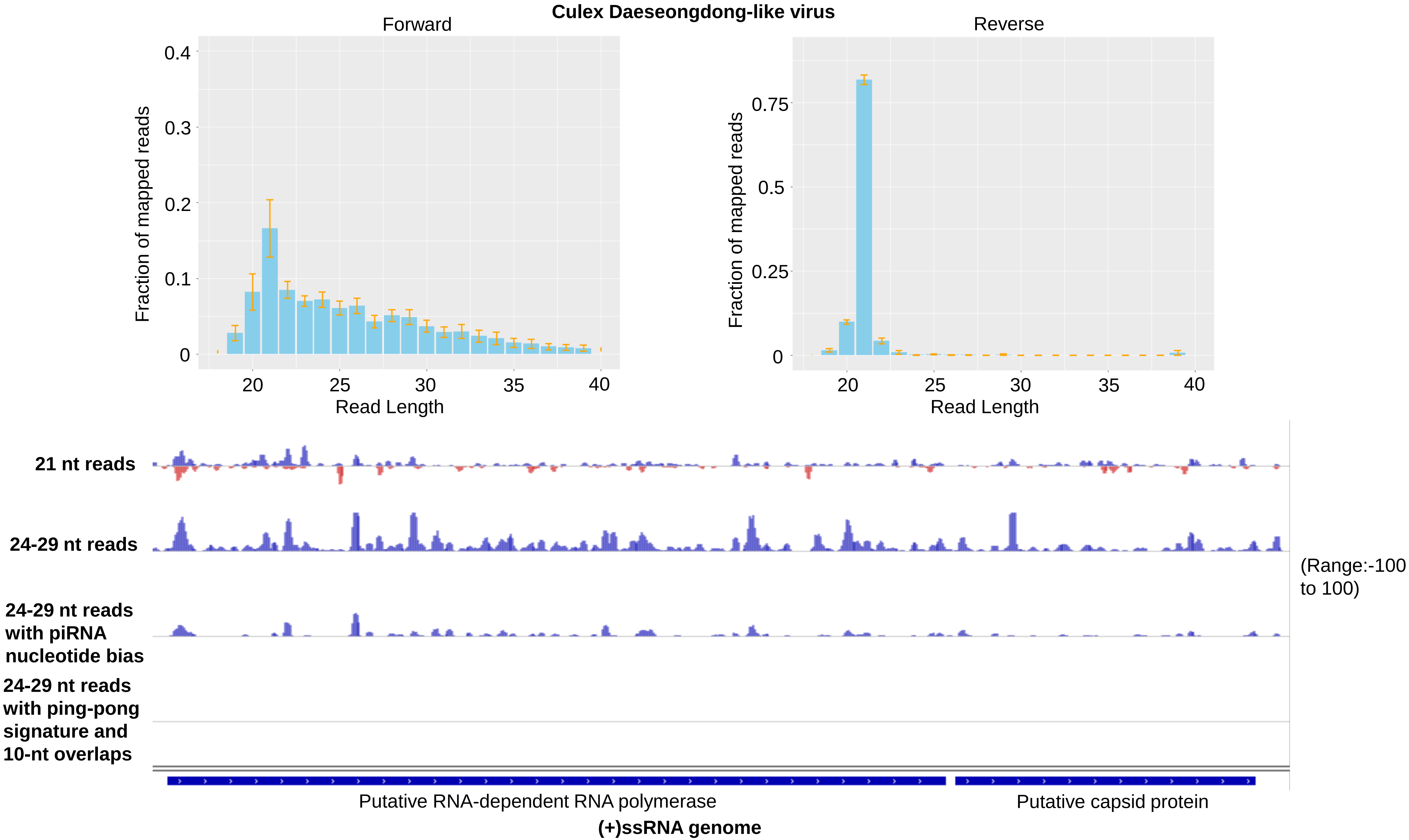
Culex Daeseongdong-like virus
Forward
Reverse
Fraction of mapped reads
0.4
0.3
0.2
0.1
0
0.75
0.5
0.25
20
25
30
35
40
Read Length
21 nt reads
24-29 nt reads
24-29 nt reads with piRNA nucleotide bias
24-29 nt reads with ping-pong signature and 10-nt overlaps
(Range:-100 to 100)
Putative RNA-dependent RNA polymerase
Putative capsid protein
(+)ssRNA genome

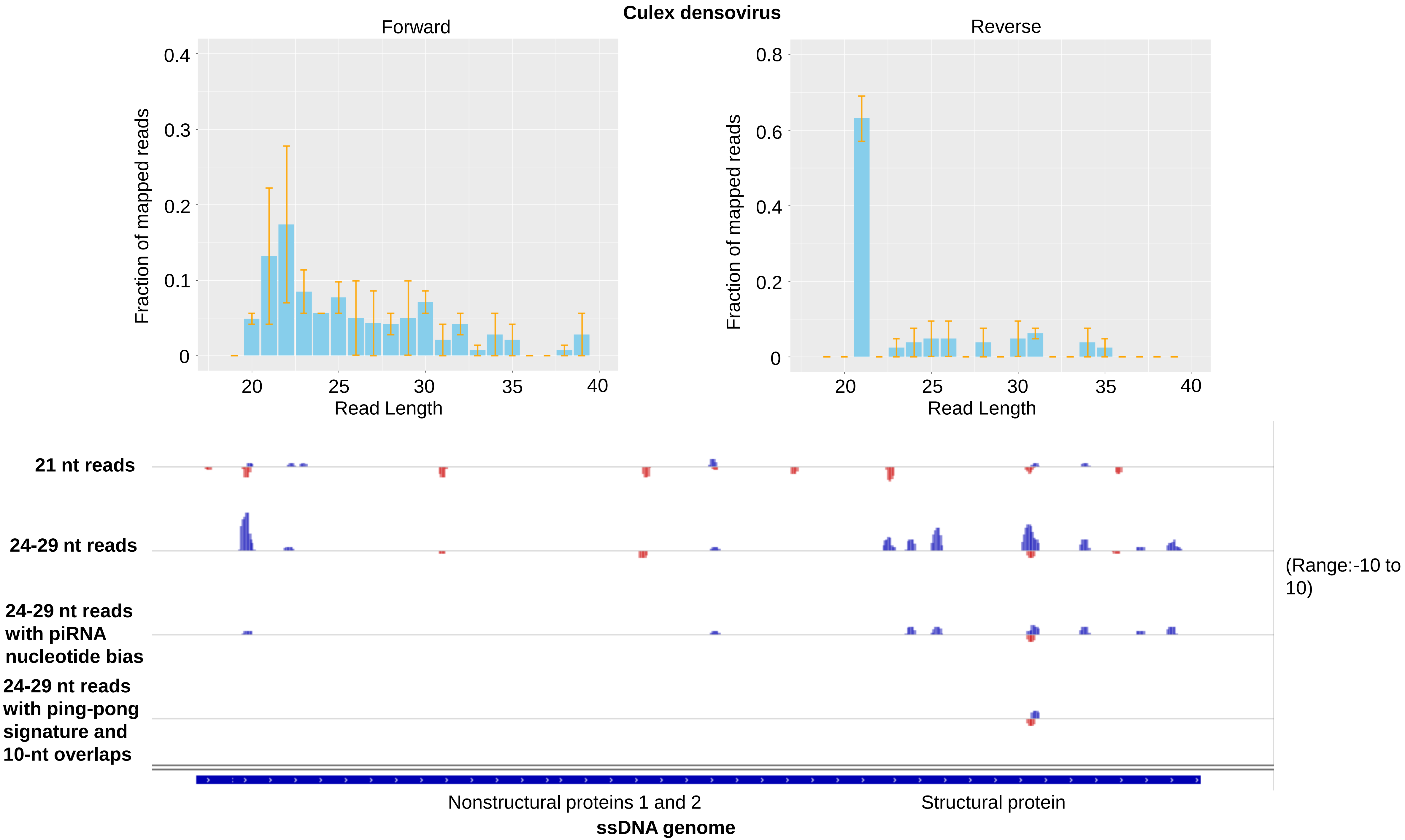
Culex densovirus
Forward
Reverse
Fraction of mapped reads
Read Length
21 nt reads
24-29 nt reads
24-29 nt reads with piRNA nucleotide bias
24-29 nt reads with ping-pong signature and 10-nt overlaps
(Range:-10 to 10)
Nonstructural proteins 1 and 2
Structural protein
ssDNA genome

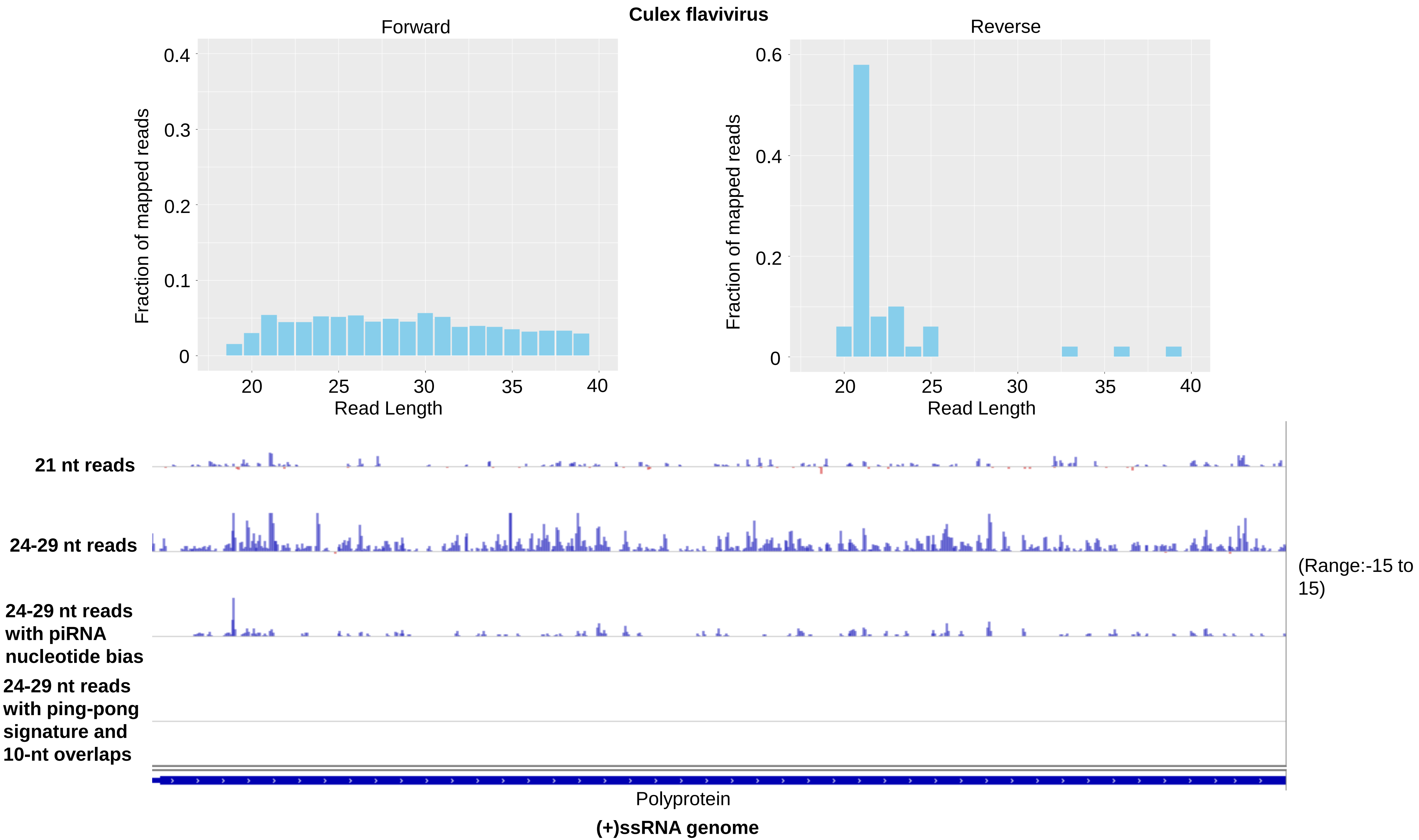

Culex flavivirus
Forward
Reverse
Fraction of mapped reads
0.4
0.3
0.2
0.1
0
0.6
20
25
30
35
40
Read Length
21 nt reads
24-29 nt reads
24-29 nt reads with piRNA nucleotide bias
24-29 nt reads with ping-pong signature and 10-nt overlaps
(Range:-15 to 15)
Polyprotein
(+)ssRNA genome

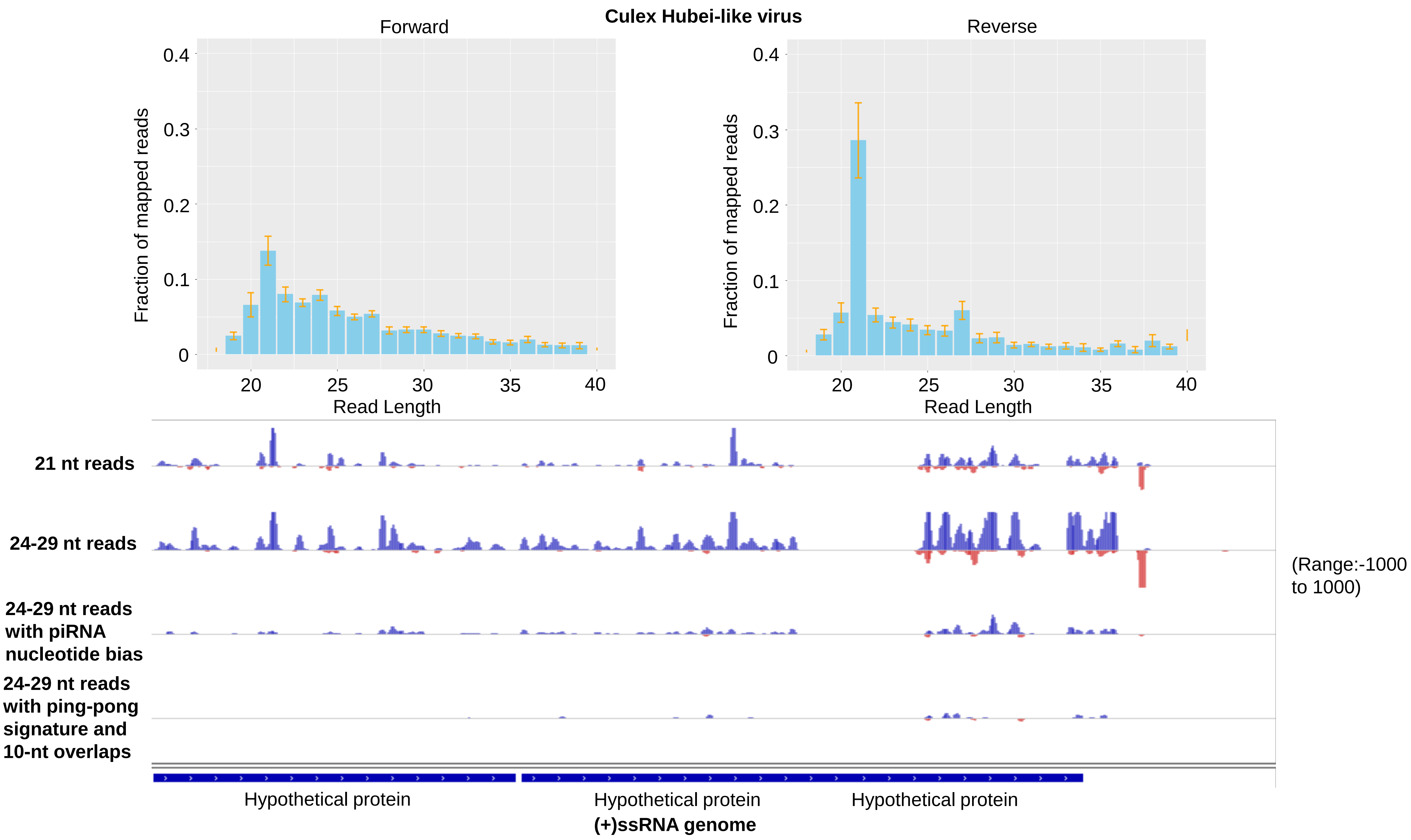

Culex Hubei-like virus
Forward
Reverse
Fraction of mapped reads
0.4
0.3
0.2
0.1
0
20
25
30
35
40
Read Length
21 nt reads
24-29 nt reads
24-29 nt reads with piRNA nucleotide bias
24-29 nt reads with ping-pong signature and 10-nt overlaps
(Range:-1000 to 1000)
Hypothetical protein
Hypothetical protein
Hypothetical protein
(+)ssRNA genome

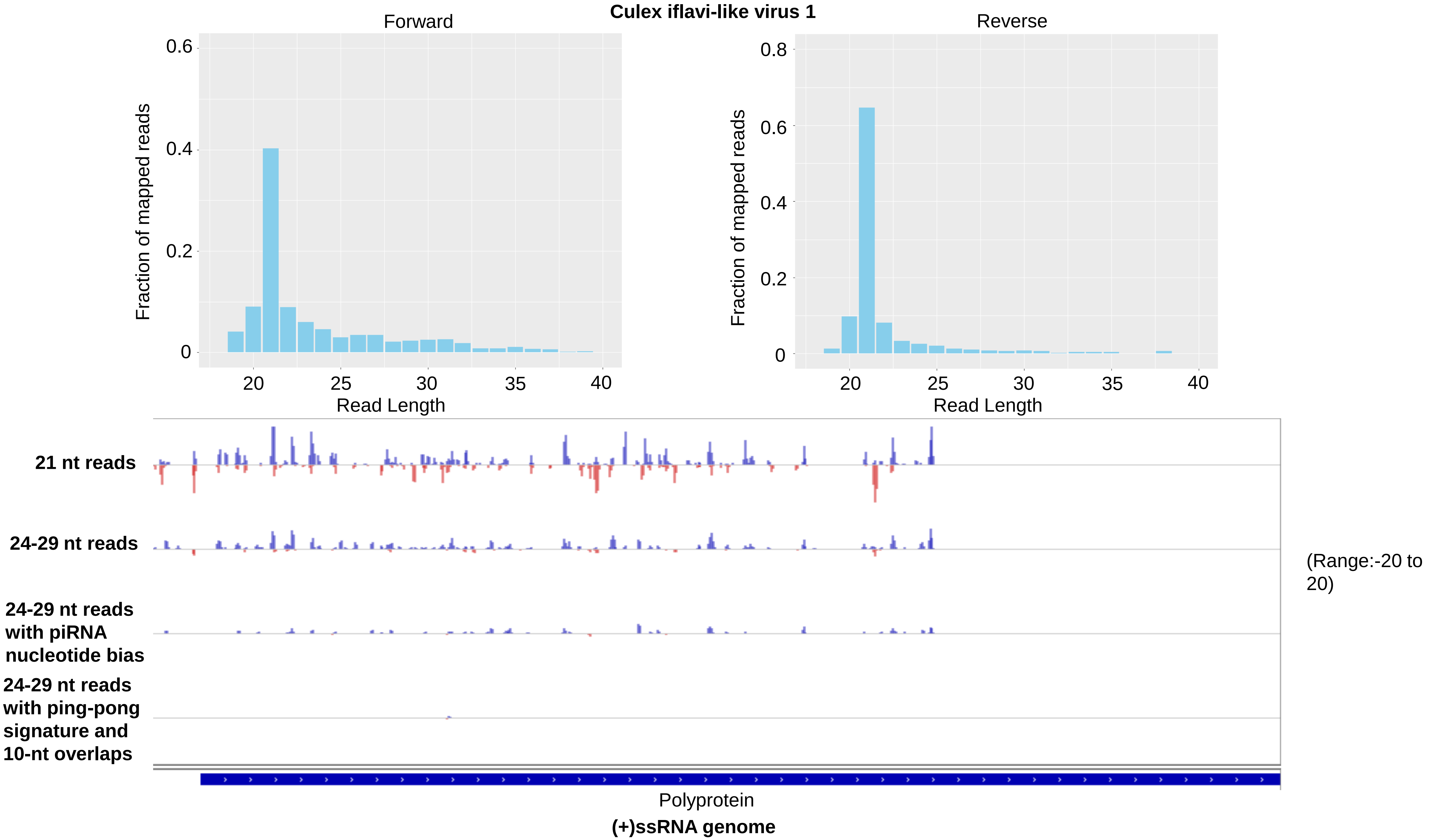
Culex iflavi-like virus 1
Forward
Reverse
Fraction of mapped reads
Read Length
0.6
0.4
0.2
0
0.8
20
25
30
35
40
21 nt reads
24-29 nt reads
24-29 nt reads with piRNA nucleotide bias
24-29 nt reads with ping-pong signature and 10-nt overlaps
(Range:-20 to 20)
Polyprotein
(+)ssRNA genome

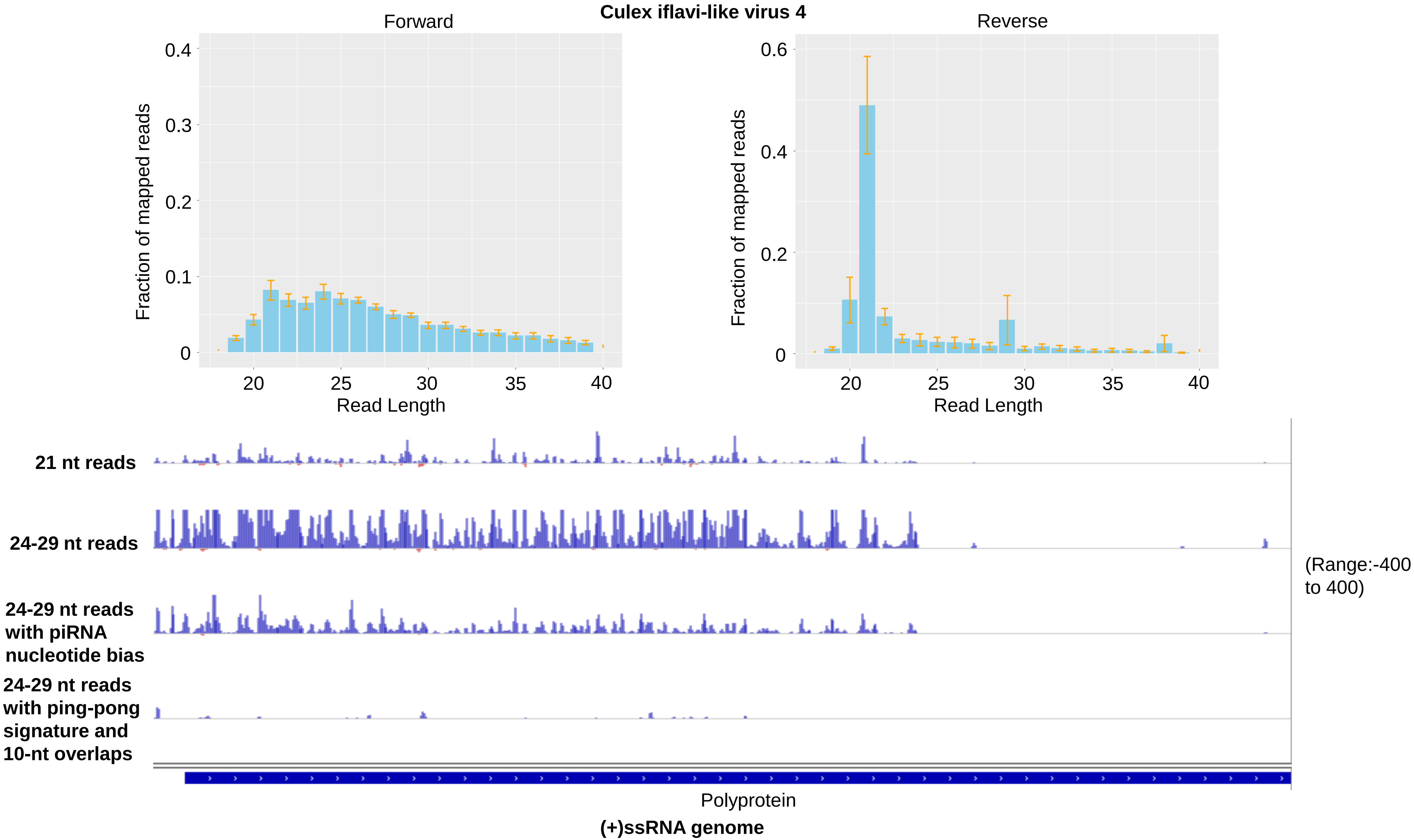

Culex iflavi-like virus 4
Forward
Reverse
Fraction of mapped reads
Read Length
21 nt reads
24-29 nt reads
24-29 nt reads with piRNA nucleotide bias
24-29 nt reads with ping-pong signature and 10-nt overlaps
(Range:-400 to 400)
Polyprotein
(+)ssRNA genome

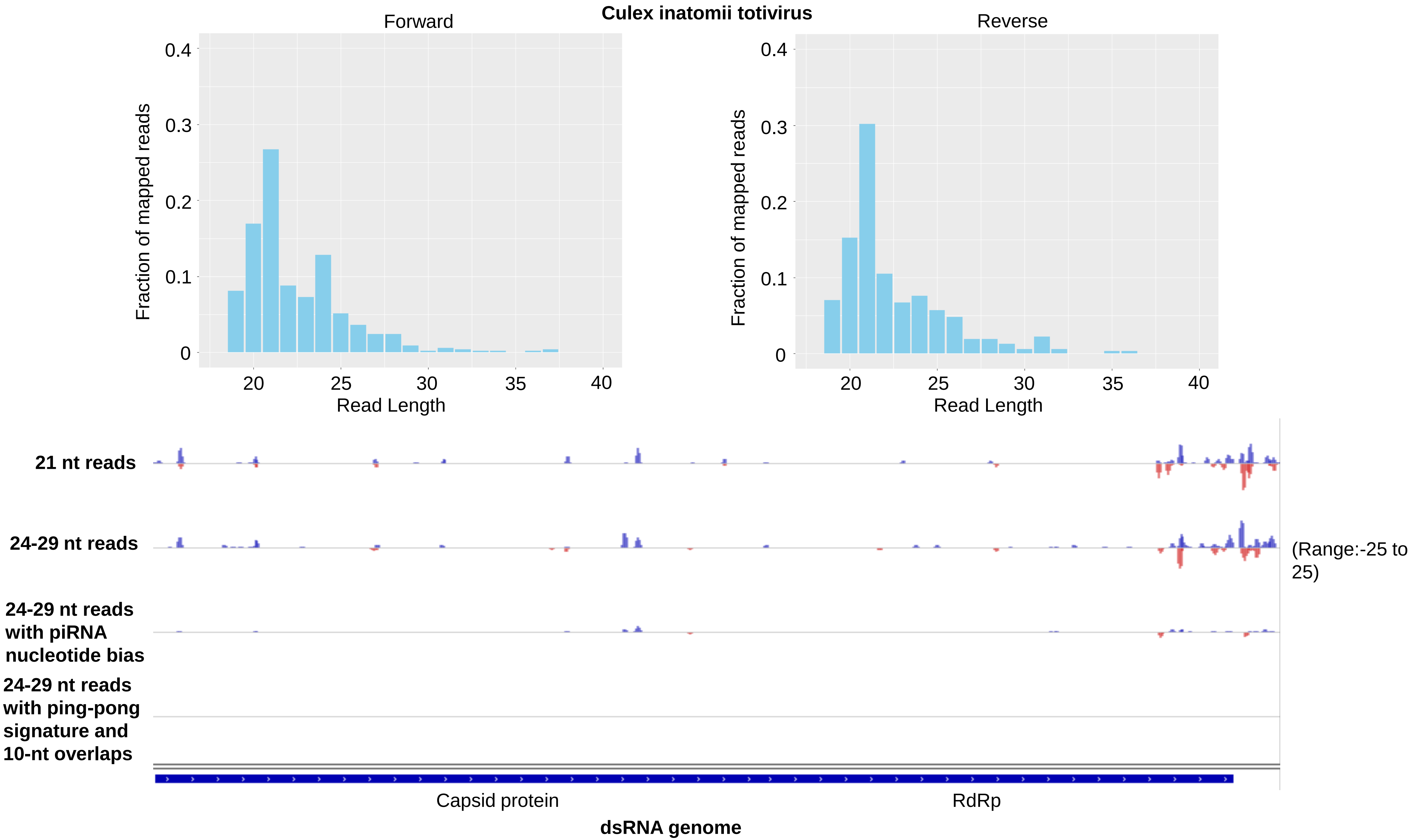

Culex inatomii totivirus
Forward
Reverse
Fraction of mapped reads
0.4
0.3
0.2
0.1
0
20
25
30
35
40
Read Length
21 nt reads
24-29 nt reads
24-29 nt reads with piRNA nucleotide bias
24-29 nt reads with ping-pong signature and 10-nt overlaps
(Range:-25 to 25)
Capsid protein
RdRp
dsRNA genome

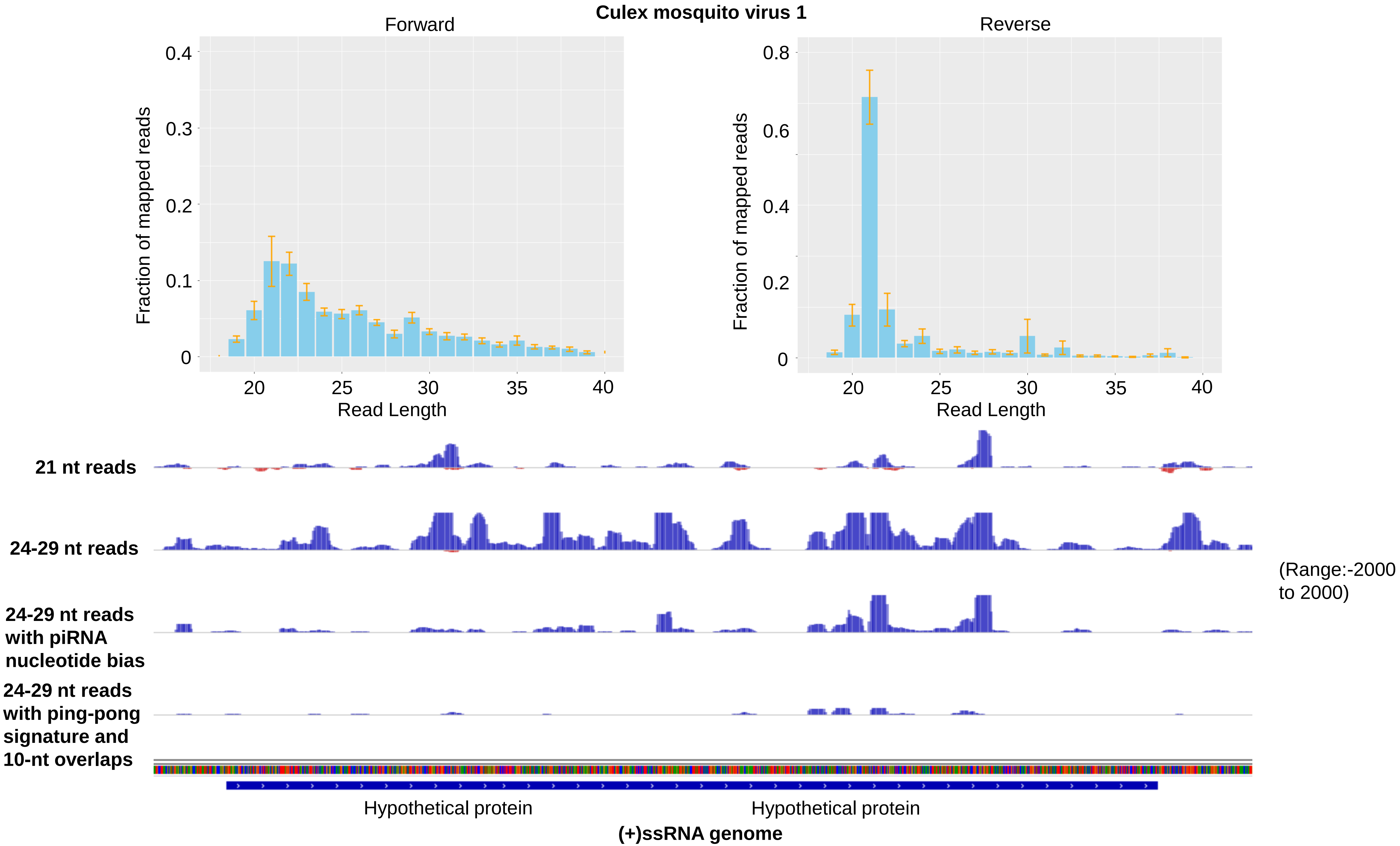

Culex mosquito virus 1
Forward
Reverse
Fraction of mapped reads
Read Length
0
0.1
0.2
0.3
0.4
0.6
0.8
20
25
30
35
40
21 nt reads
24-29 nt reads
24-29 nt reads with piRNA nucleotide bias
24-29 nt reads with ping-pong signature and 10-nt overlaps
(Range:-2000 to 2000)
Hypothetical protein
Hypothetical protein
(+)ssRNA genome

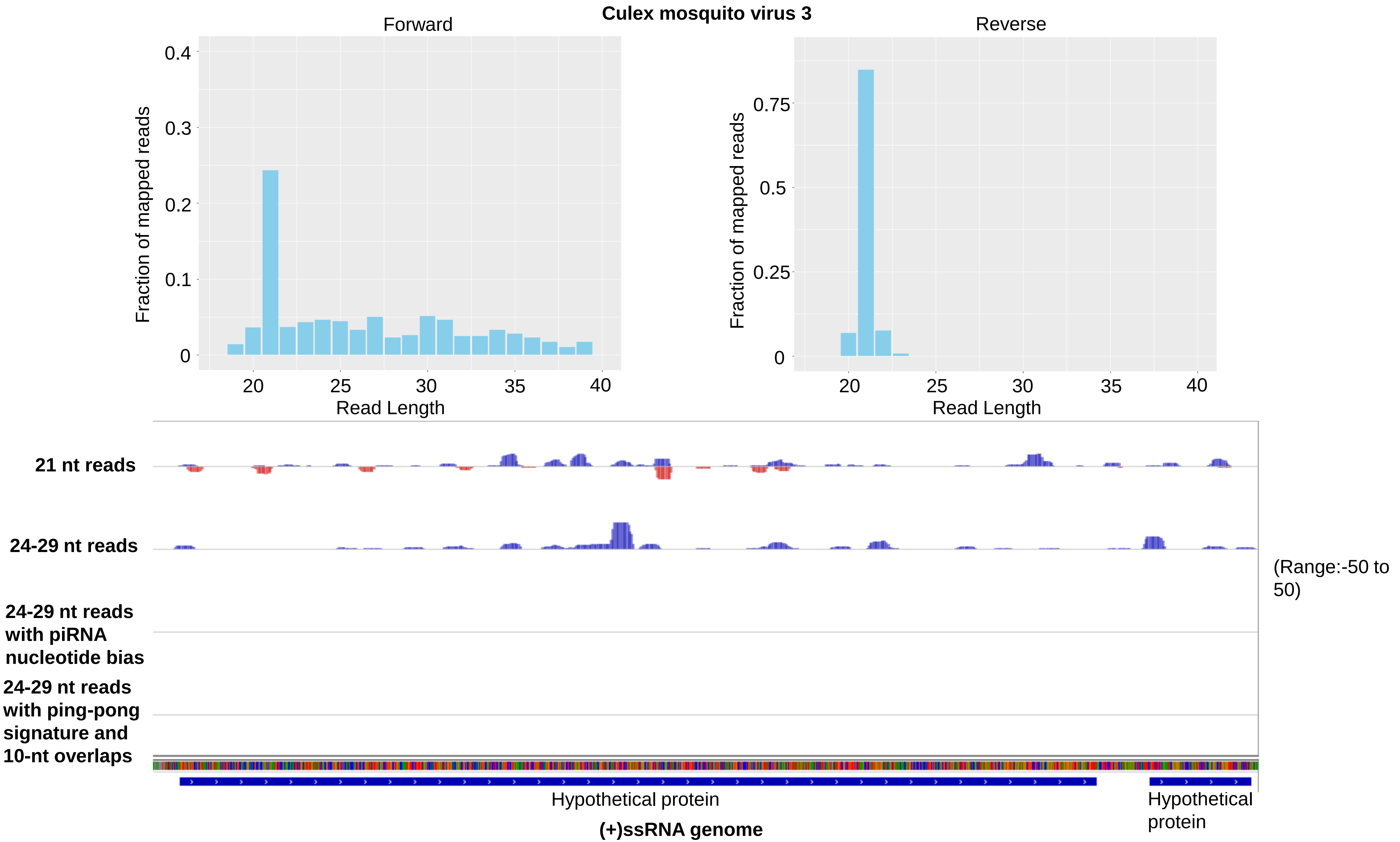
Culex mosquito virus 3
Forward
Reverse
Fraction of mapped reads
Read Length
21 nt reads
24-29 nt reads
24-29 nt reads with piRNA nucleotide bias
24-29 nt reads with ping-pong signature and 10-nt overlaps
(Range:-50 to 50)
Hypothetical protein
Hypothetical protein
(+)ssRNA genome

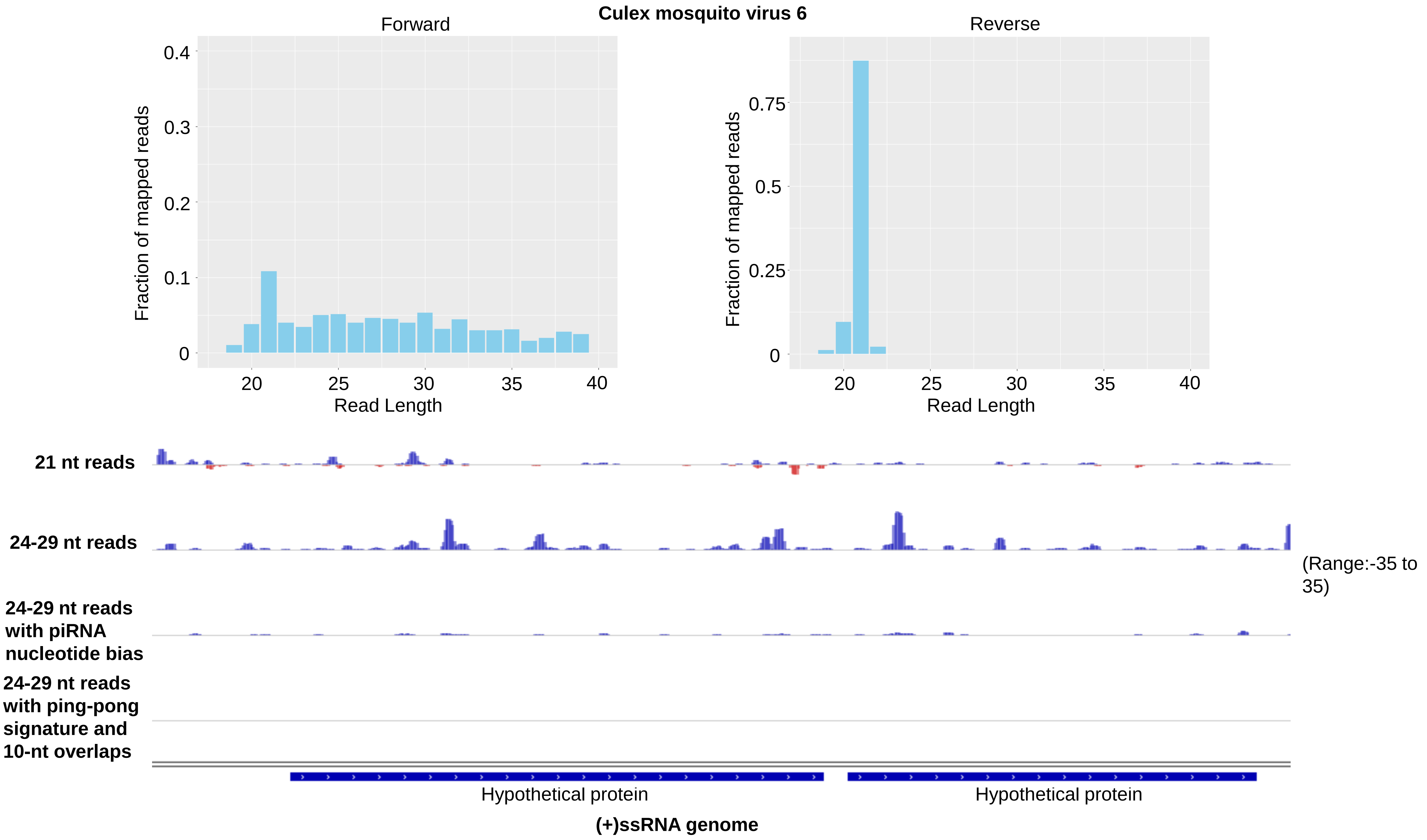

Culex mosquito virus 6
Forward
Reverse
Fraction of mapped reads
0.4
0.3
0.2
0.1
0
0.75
0.5
0.25
20
25
30
35
40
Read Length
21 nt reads
24-29 nt reads
24-29 nt reads with piRNA nucleotide bias
24-29 nt reads with ping-pong signature and 10-nt overlaps
(Range:-35 to 35)
Hypothetical protein
Hypothetical protein
(+)ssRNA genome

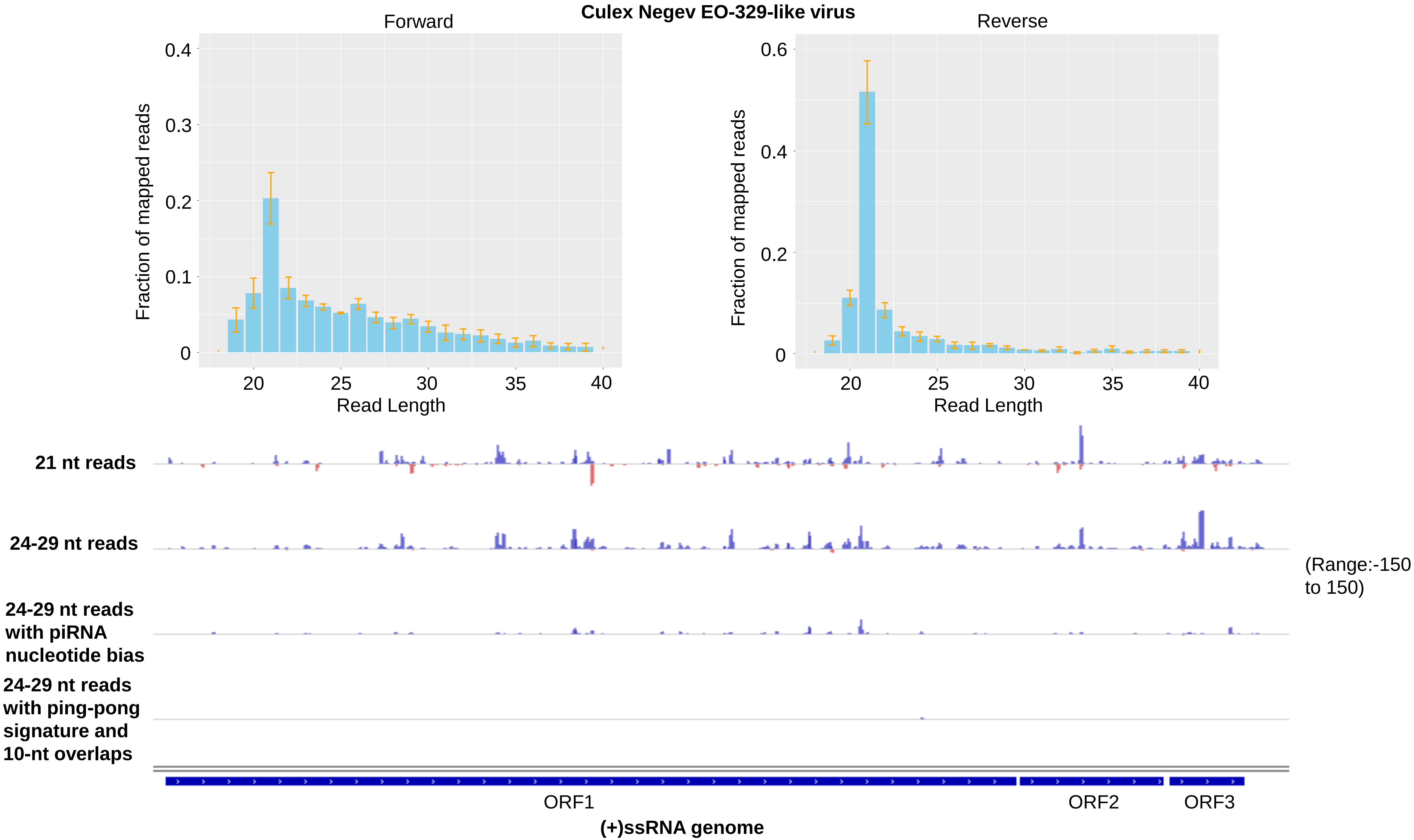

Culex Negev EO-329-like virus
Forward
Reverse
Fraction of mapped reads
0.4
0.3
0.2
0.1
0
0.6
Read Length
20
25
30
35
40
21 nt reads
24-29 nt reads
24-29 nt reads with piRNA nucleotide bias
24-29 nt reads with ping-pong signature and 10-nt overlaps
(Range:-150 to 150)
ORF1
ORF2
ORF3
(+)ssRNA genome

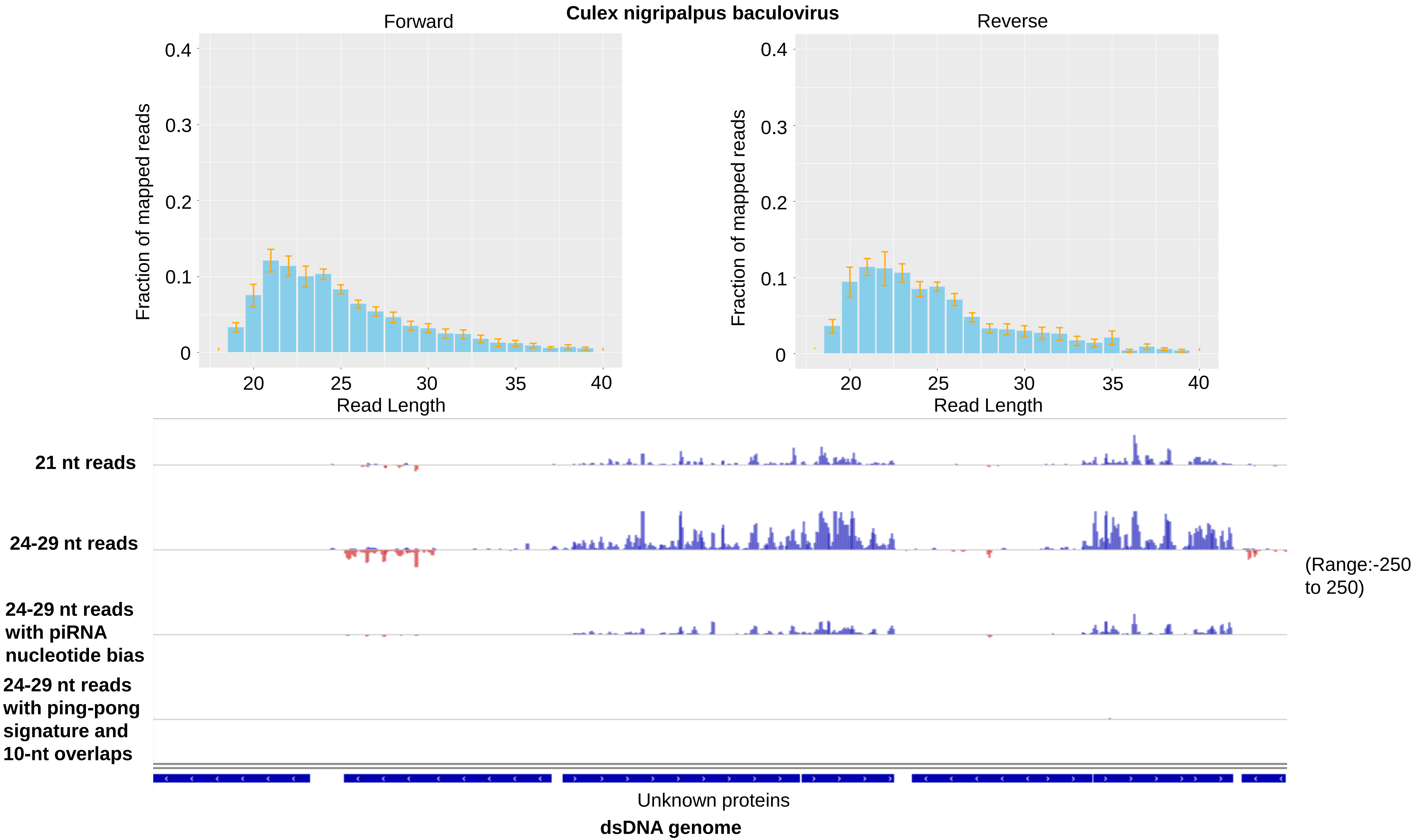

Culex nigripalpus baculovirus
Forward
Reverse
Fraction of mapped reads
0.4
0.3
0.2
0.1
0
20
25
30
35
40
Read Length
21 nt reads
24-29 nt reads
24-29 nt reads with piRNA nucleotide bias
24-29 nt reads with ping-pong signature and 10-nt overlaps
(Range:-250 to 250)
Unknown proteins
dsDNA genome

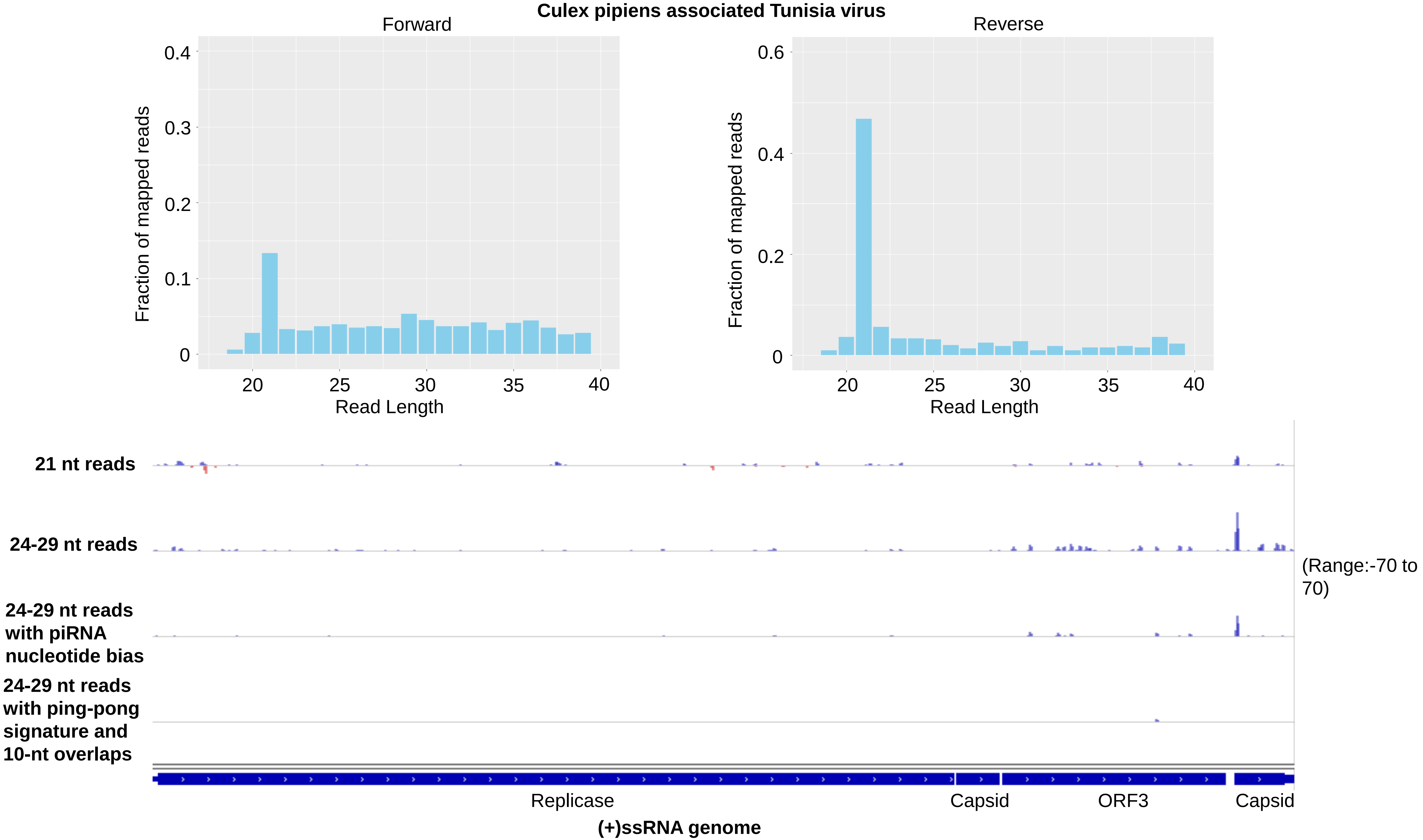

Culex pipiens associated Tunisia virus
Forward
Reverse
Fraction of mapped reads
Read Length
0.4
0.3
0.2
0.1
0
0.6
20
25
30
35
40
21 nt reads
24-29 nt reads
24-29 nt reads with piRNA nucleotide bias
24-29 nt reads with ping-pong signature and 10-nt overlaps
(Range:-70 to 70)
Replicase
Capsid
ORF3
Capsid
(+)ssRNA genome

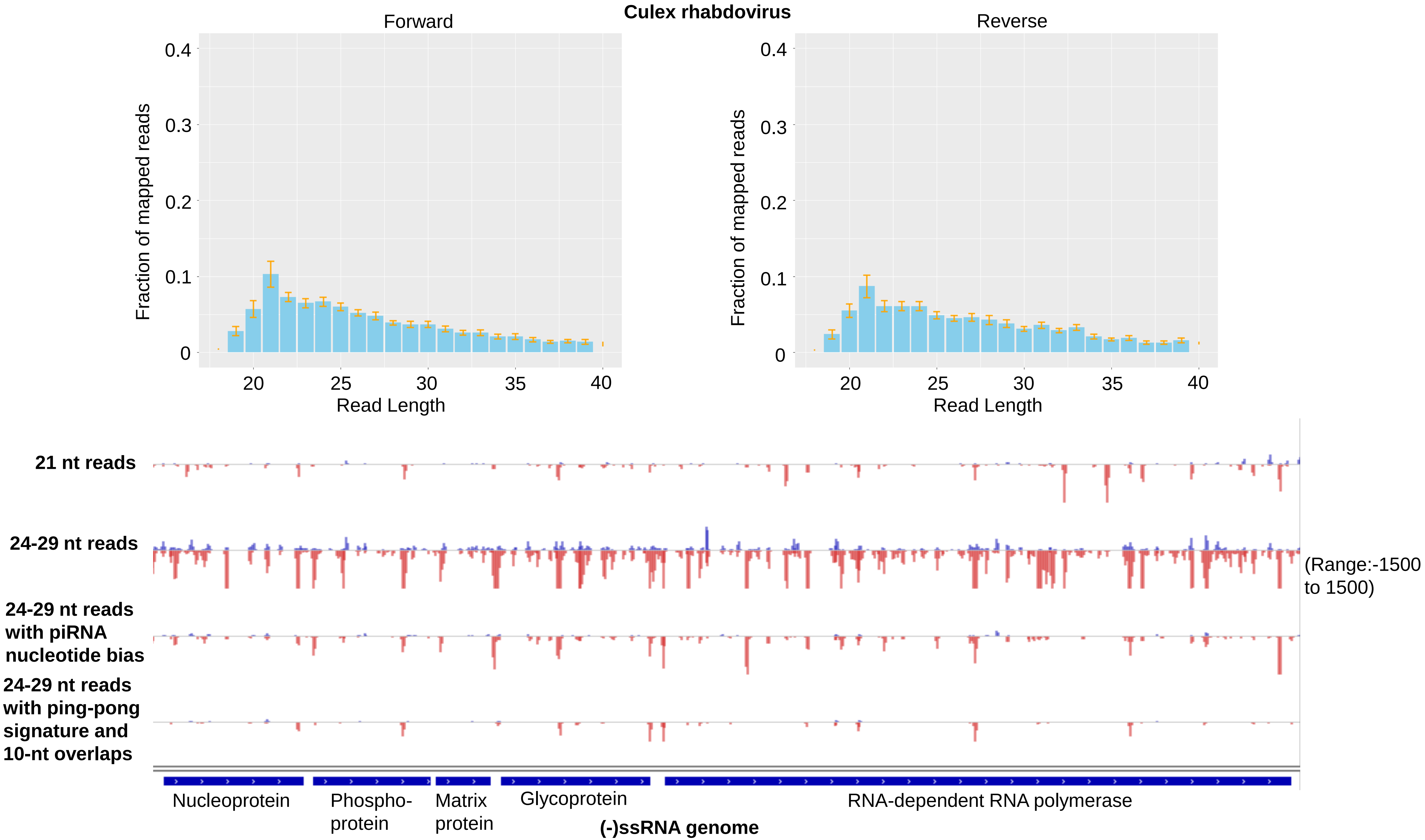

Culex rhabdovirus
Forward
Reverse
Fraction of mapped reads
0.4
0.3
0.2
0.1
0
20
25
30
35
40
Read Length
21 nt reads
24-29 nt reads
24-29 nt reads with piRNA nucleotide bias
24-29 nt reads with ping-pong signature and 10-nt overlaps
(Range:-1500 to 1500)
Nucleoprotein
Phospho-protein
Matrix protein
Glycoprotein
RNA-dependent RNA polymerase
(-)ssRNA genome

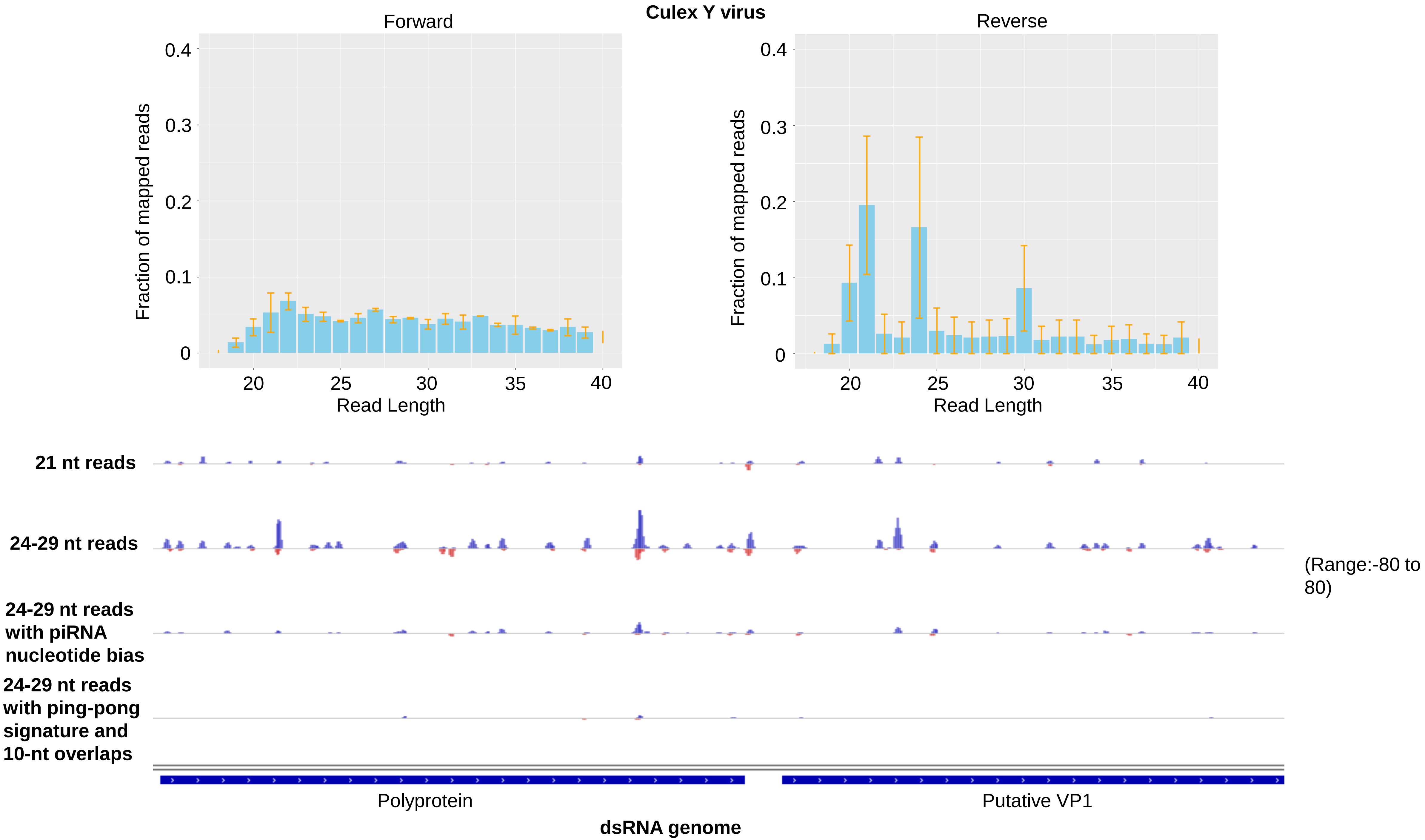
Culex Y virus
Forward
Reverse
Fraction of mapped reads
Read Length
0
0.1
0.2
0.3
0.4
20
25
30
35
40
21 nt reads
24-29 nt reads
24-29 nt reads with piRNA nucleotide bias
24-29 nt reads with ping-pong signature and 10-nt overlaps
(Range:-80 to 80)
Polyprotein
Putative VP1
dsRNA genome

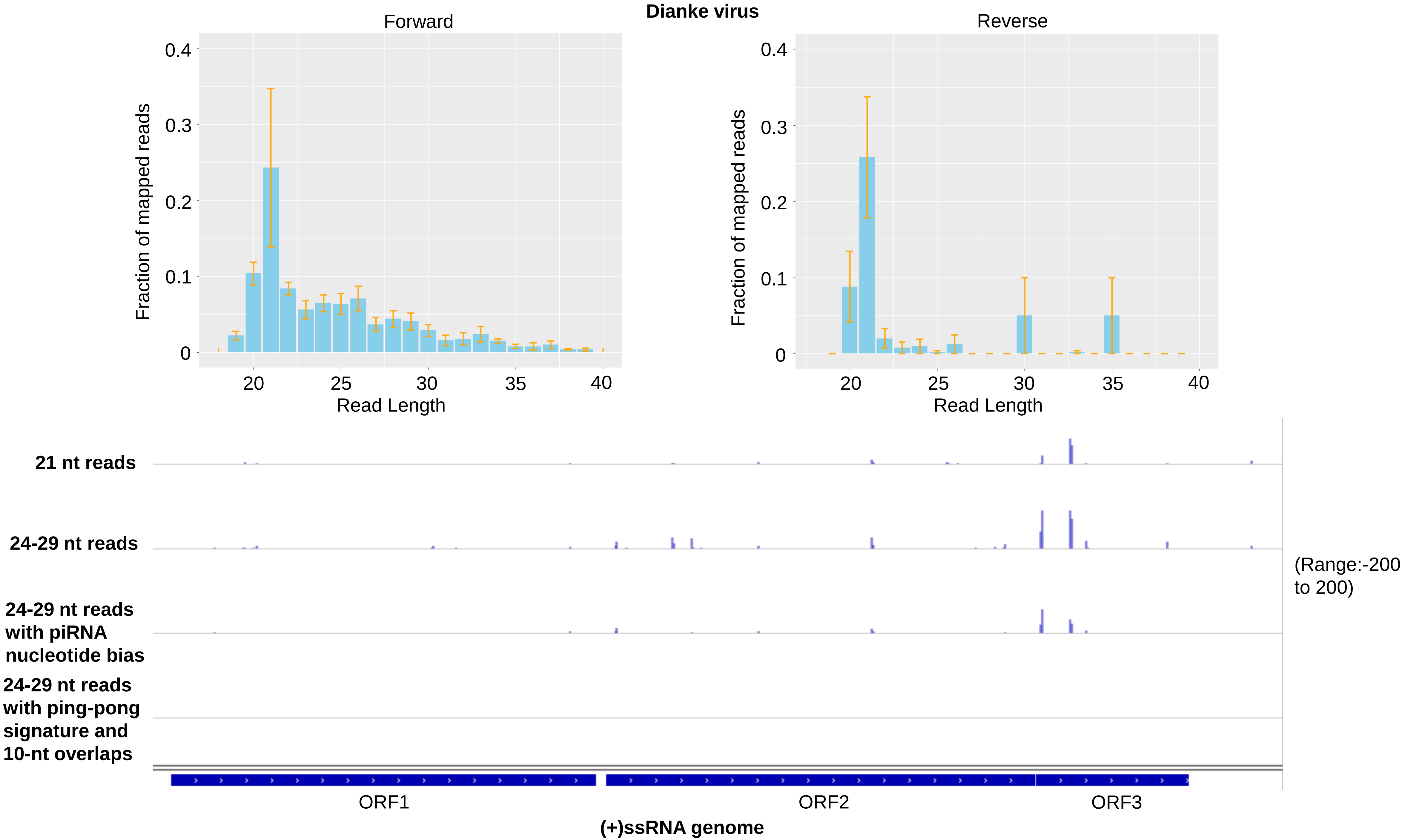
Dianke virus
Forward
Reverse
Fraction of mapped reads
Read Length
0
0.1
0.2
0.3
0.4
20
25
30
35
40
21 nt reads
24-29 nt reads
24-29 nt reads with piRNA nucleotide bias
24-29 nt reads with ping-pong signature and 10-nt overlaps
(Range:-200 to 200)
ORF1
ORF2
ORF3
(+)ssRNA genome

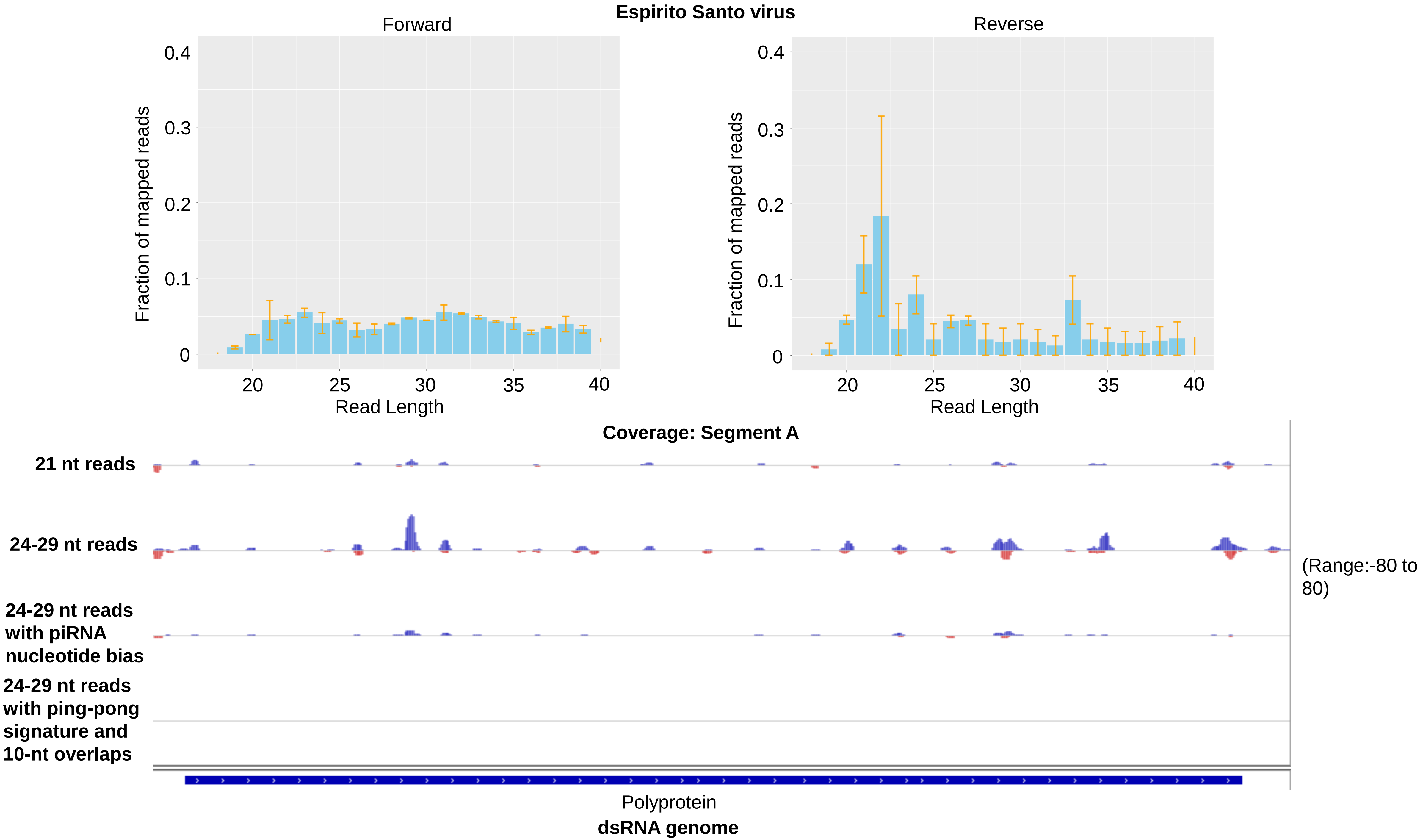

Espirito Santo virus
Forward
Reverse
Fraction of mapped reads
0.4
0.3
0.2
0.1
0
20
25
30
35
40
Read Length
Coverage: Segment A
21 nt reads
24-29 nt reads
24-29 nt reads with piRNA nucleotide bias
24-29 nt reads with ping-pong signature and 10-nt overlaps
(Range:-80 to 80)
Polyprotein
dsRNA genome

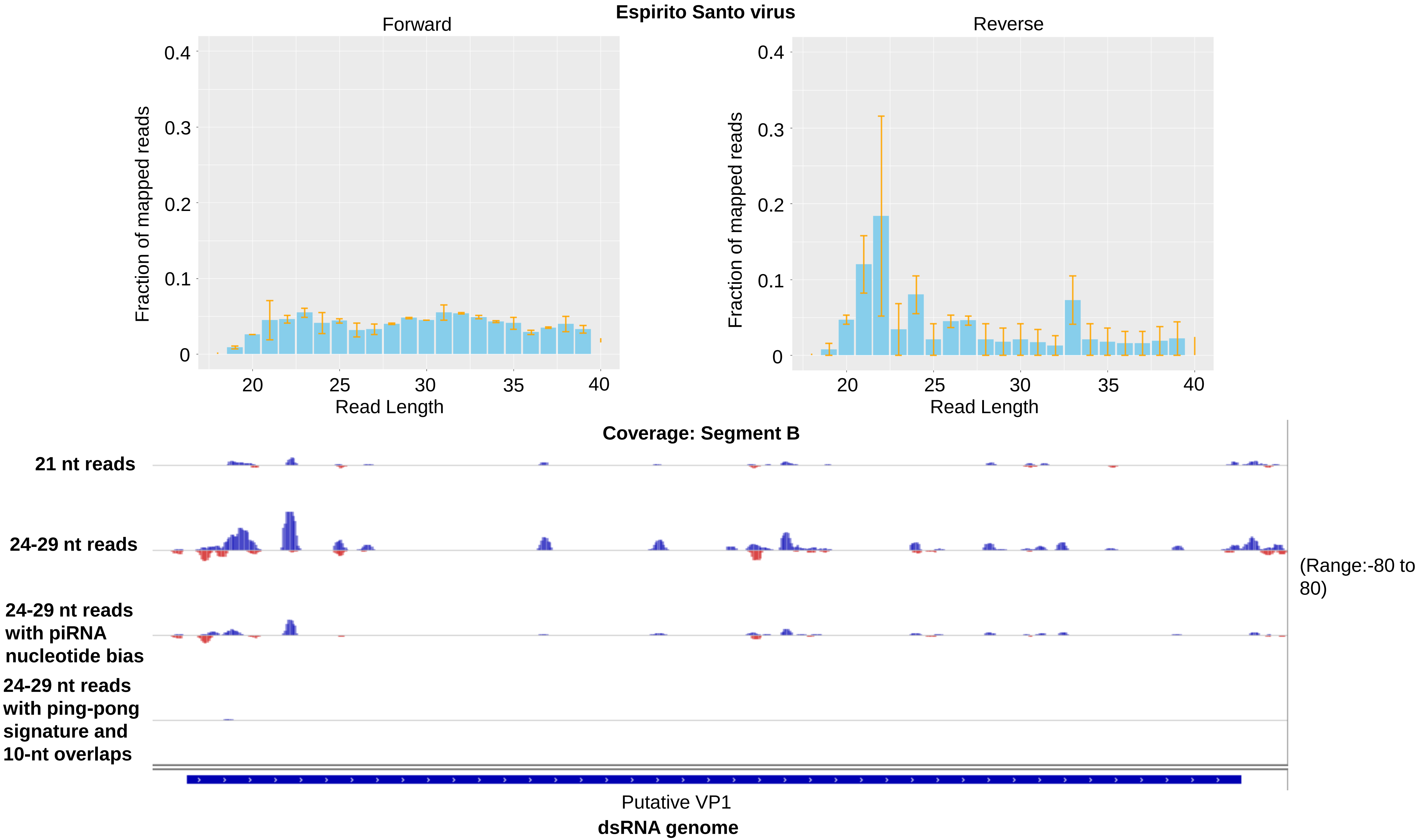

Espirito Santo virus
Forward
Reverse
Fraction of mapped reads
0
0.1
0.2
0.3
0.4
20
25
30
35
40
Read Length
Coverage: Segment B
21 nt reads
24-29 nt reads
24-29 nt reads with piRNA nucleotide bias
24-29 nt reads with ping-pong signature and 10-nt overlaps
(Range:-80 to 80)
Putative VP1
dsRNA genome

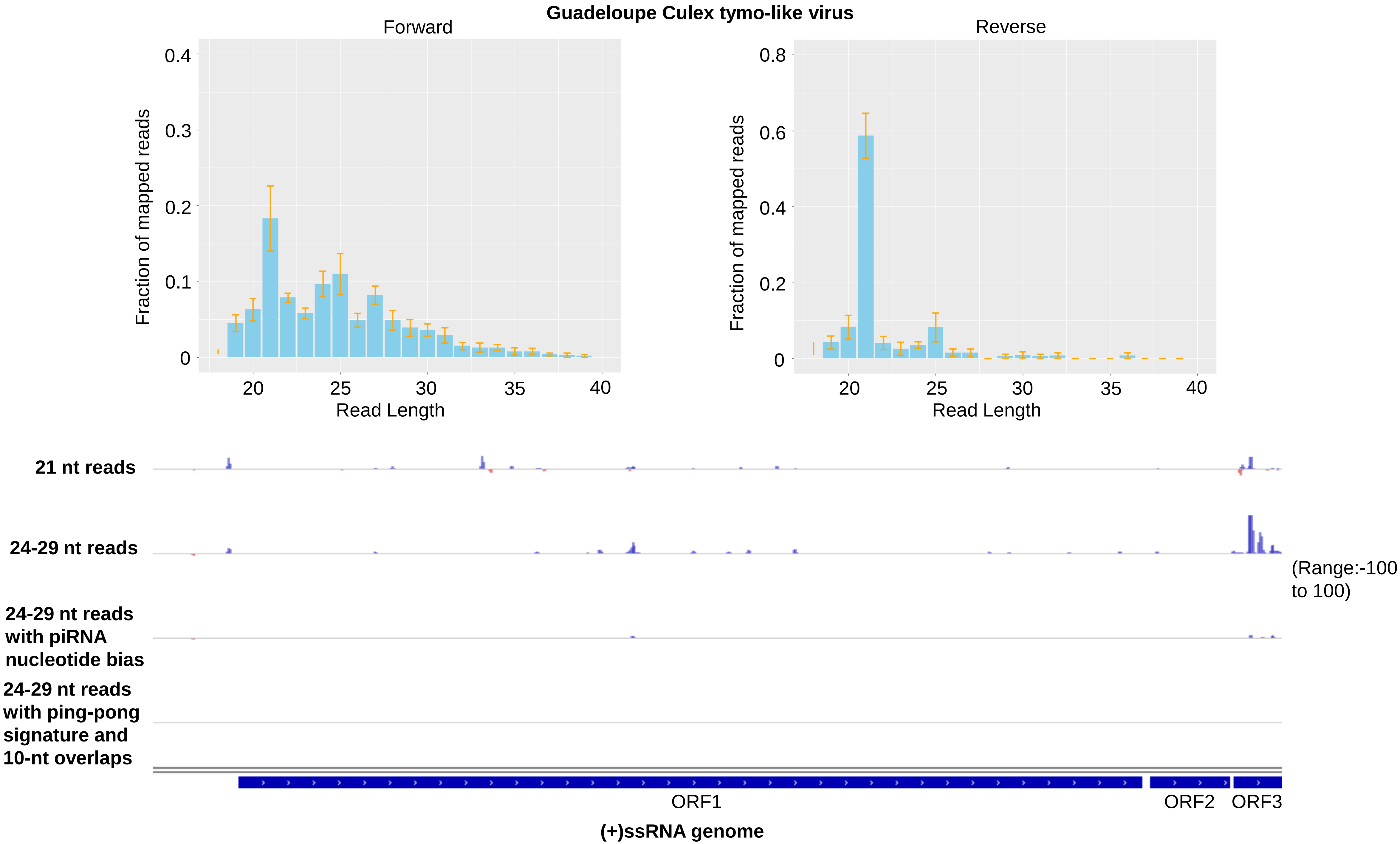

Guadeloupe Culex tymo-like virus
Forward
Reverse
Fraction of mapped reads
Read Length
0
0.1
0.2
0.3
0.4
0.6
0.8
20
25
30
35
40
21 nt reads
24-29 nt reads
24-29 nt reads with piRNA nucleotide bias
24-29 nt reads with ping-pong signature and 10-nt overlaps
(Range:-100 to 100)
ORF1
ORF2
ORF3
(+)ssRNA genome

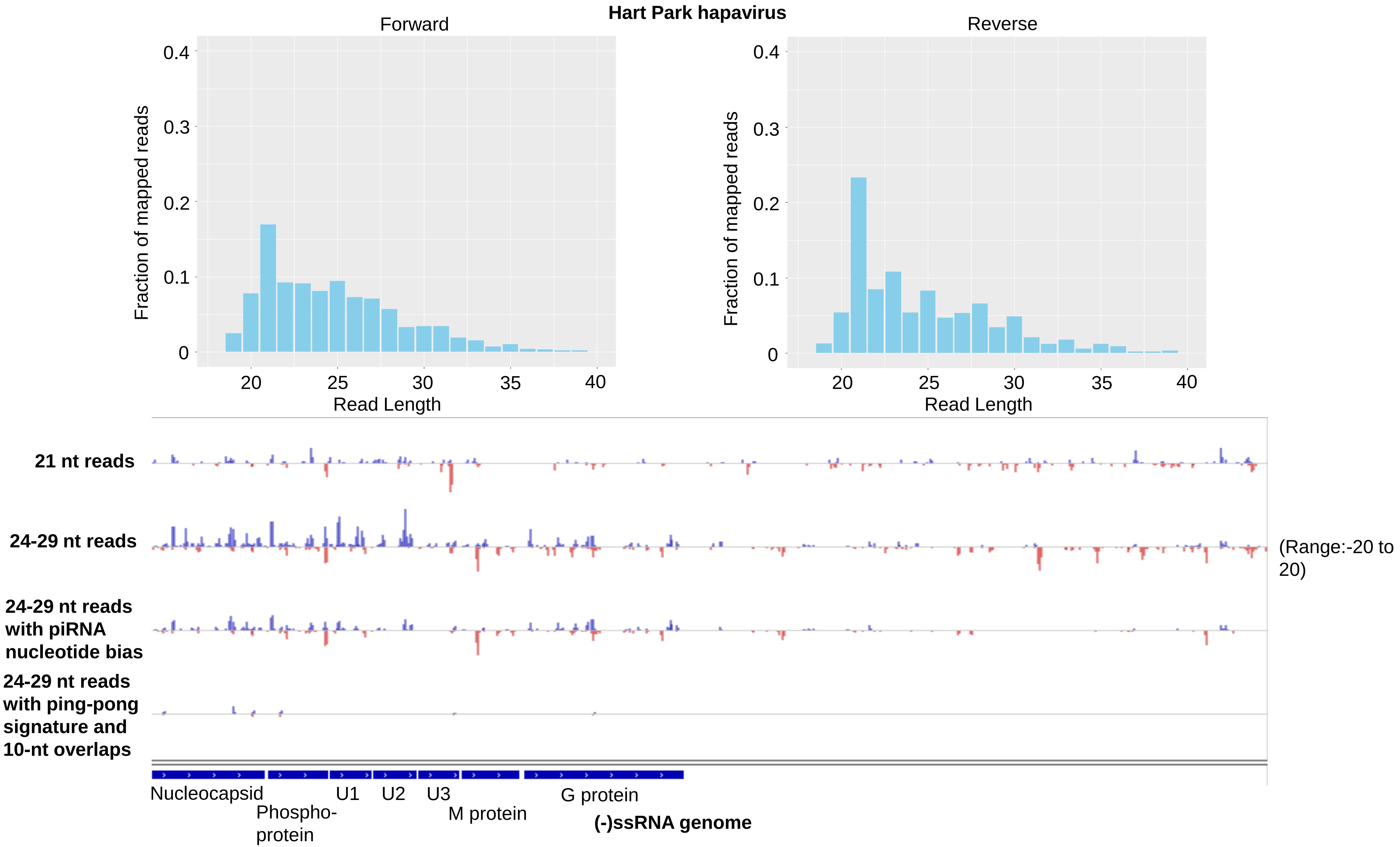
Hart Park hapavirus
Forward
Reverse
Fraction of mapped reads
Read Length
0.4
0.3
0.2
0.1
0
20
25
30
35
40
21 nt reads
24-29 nt reads
24-29 nt reads with piRNA nucleotide bias
24-29 nt reads with ping-pong signature and 10-nt overlaps
(Range:-20 to 20)
Nucleocapsid
Phospho-protein
U1
U2
U3
M protein
G protein
(-)ssRNA genome

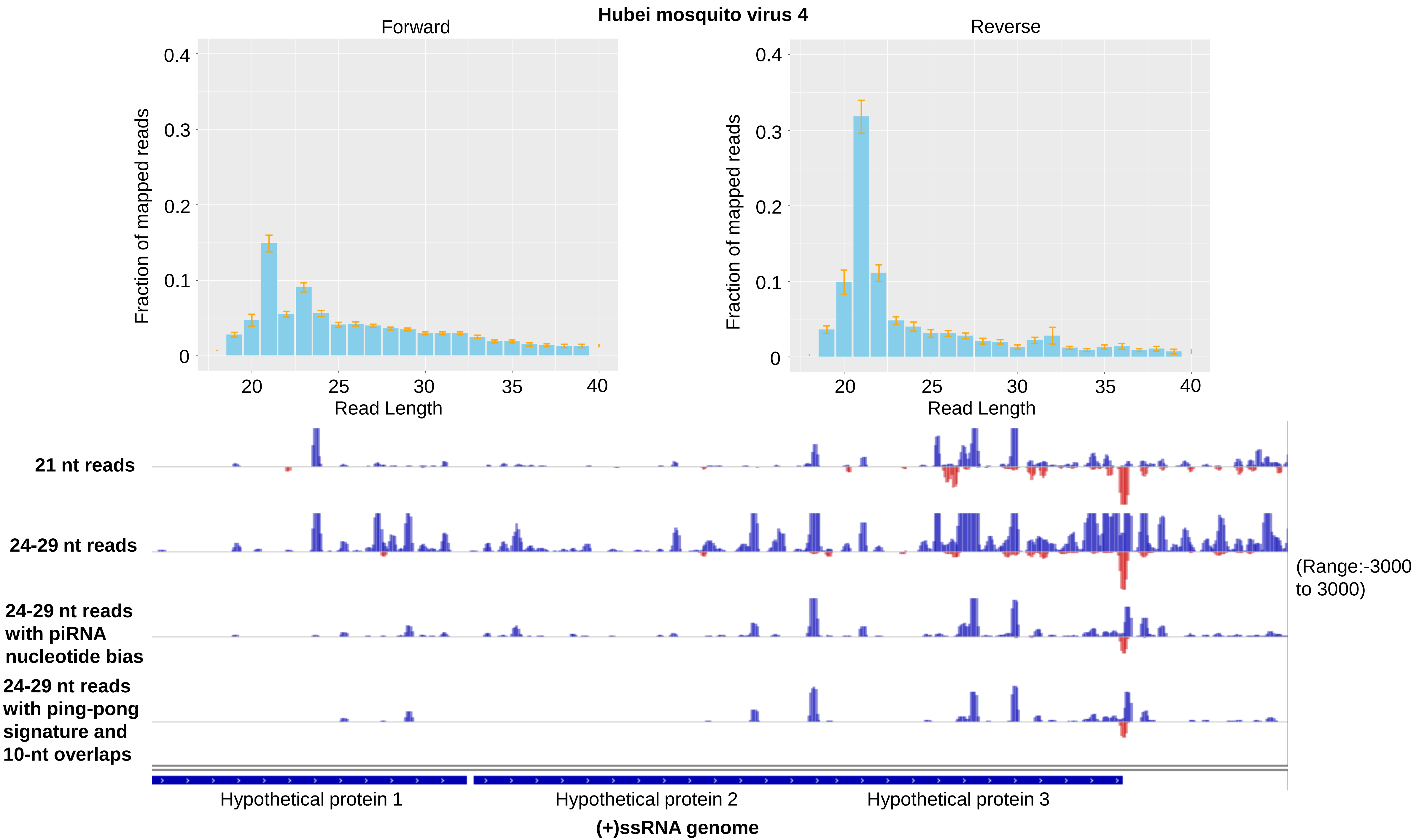

Hubei mosquito virus 4
Forward
Reverse
Fraction of mapped reads
0
0.1
0.2
0.3
0.4
20
25
30
35
40
Read Length
21 nt reads
24-29 nt reads
24-29 nt reads with piRNA nucleotide bias
24-29 nt reads with ping-pong signature and 10-nt overlaps
(Range:-3000 to 3000)
Hypothetical protein 1
Hypothetical protein 2
Hypothetical protein 3
(+)ssRNA genome

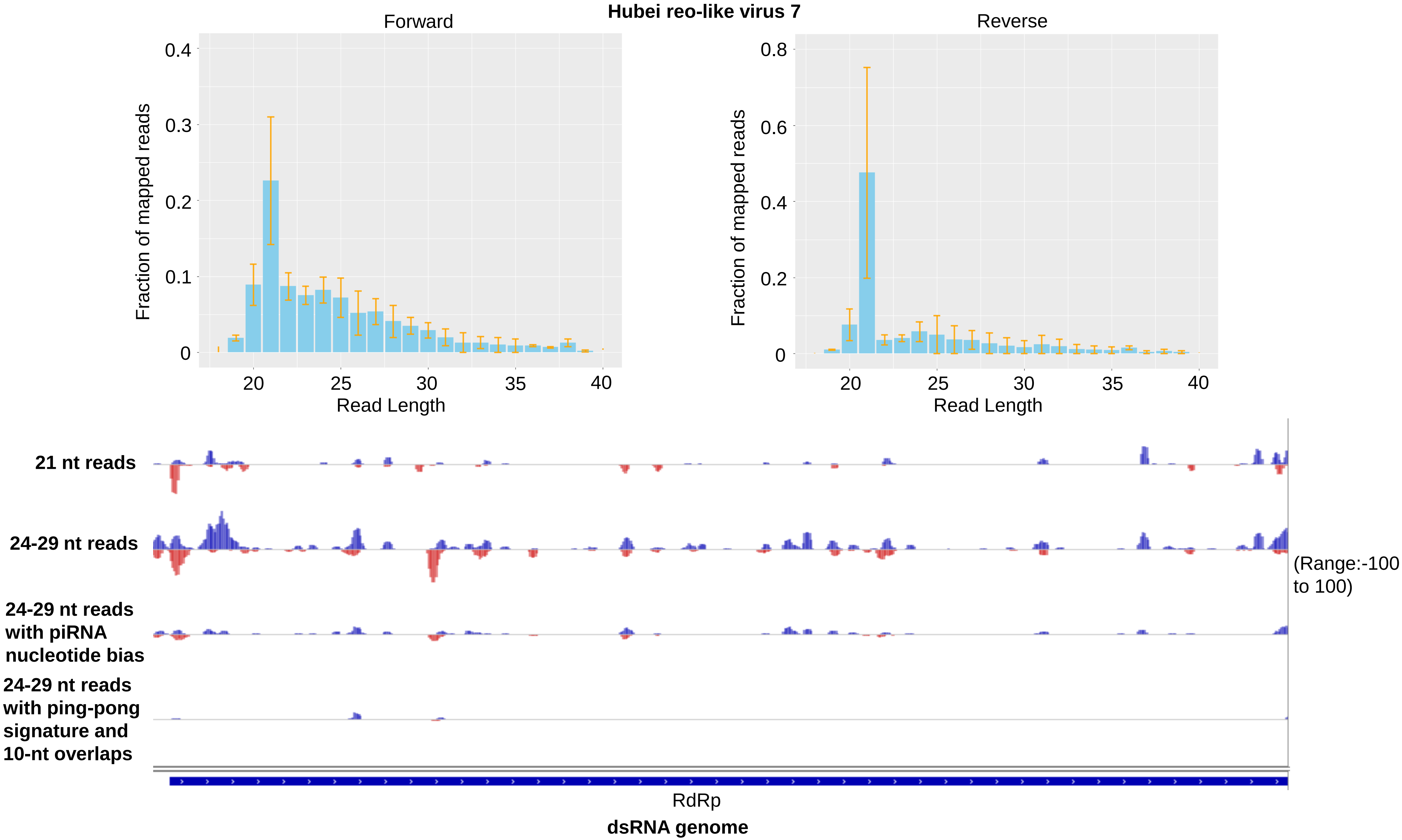

Hubei reo-like virus 7
Forward
Reverse
Fraction of mapped reads
0
0.1
0.2
0.3
0.4
0.6
0.8
20
25
30
35
40
Read Length
21 nt reads
24-29 nt reads
24-29 nt reads with piRNA nucleotide bias
24-29 nt reads with ping-pong signature and 10-nt overlaps
(Range:-100 to 100)
RdRp
dsRNA genome

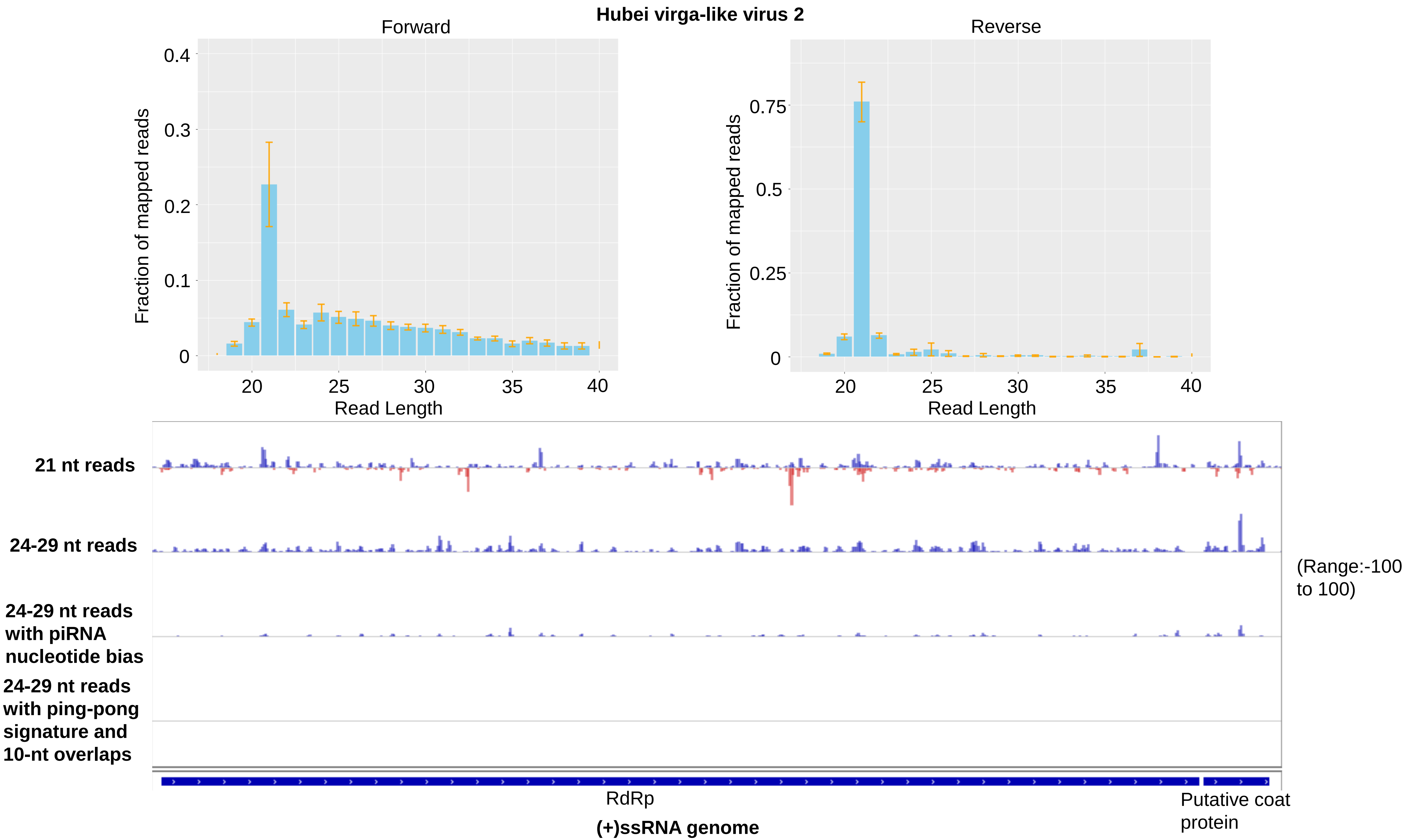

Hubei virga-like virus 2
Forward
Reverse
Fraction of mapped reads
0.4
0.3
0.2
0.1
0
0.75
0.5
0.25
20
25
30
35
40
Read Length
21 nt reads
24-29 nt reads
24-29 nt reads with piRNA nucleotide bias
24-29 nt reads with ping-pong signature and 10-nt overlaps
(Range:-100 to 100)
RdRp
Putative coat protein
(+)ssRNA genome

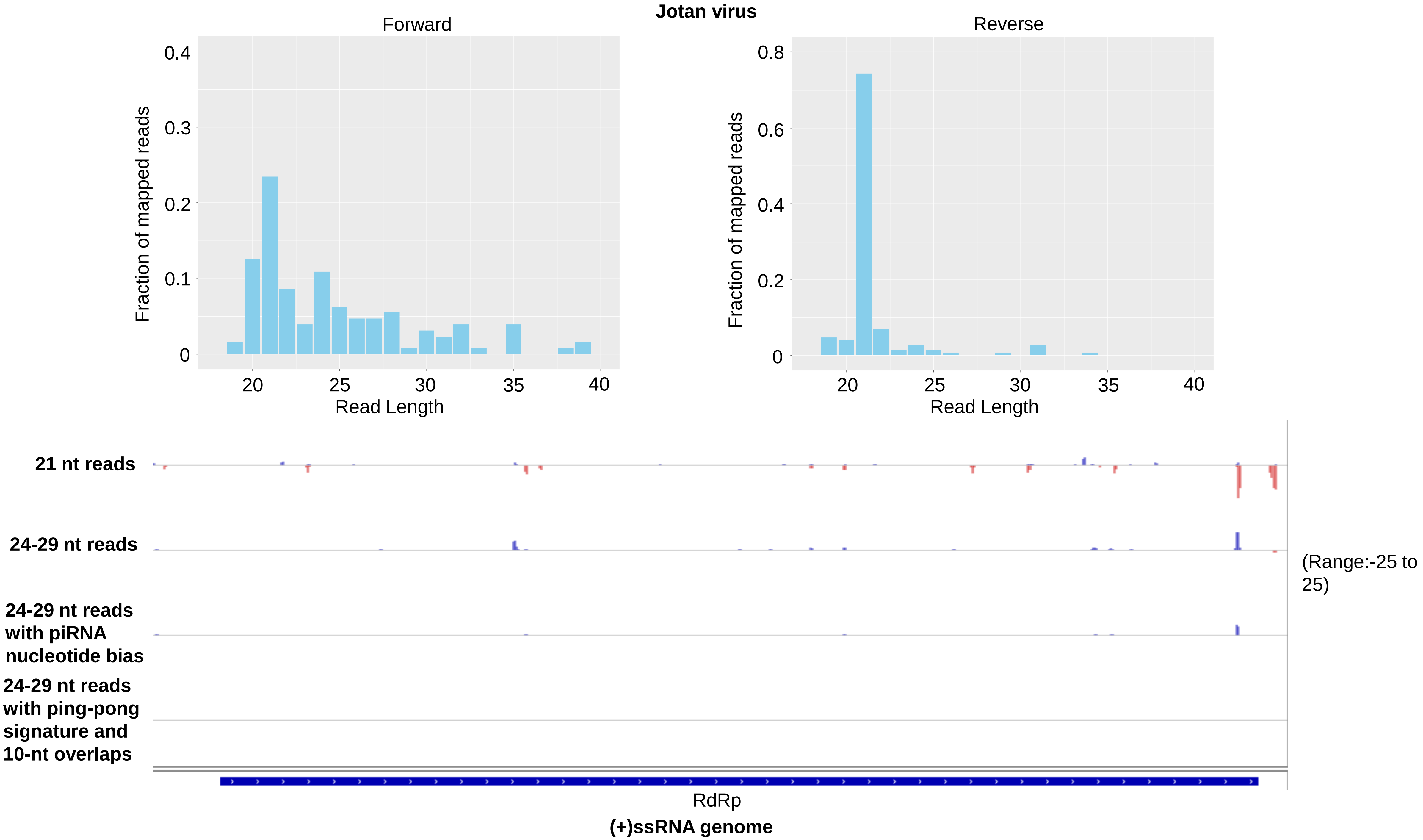
Jotan virus
Forward
Reverse
Fraction of mapped reads
0.4
0.3
0.2
0.1
0
0.8
0.6
0.4
0.2
0
20
25
30
35
40
Read Length
21 nt reads
24-29 nt reads
24-29 nt reads with piRNA nucleotide bias
24-29 nt reads with ping-pong signature and 10-nt overlaps
(Range:-25 to 25)
RdRp
(+)ssRNA genome

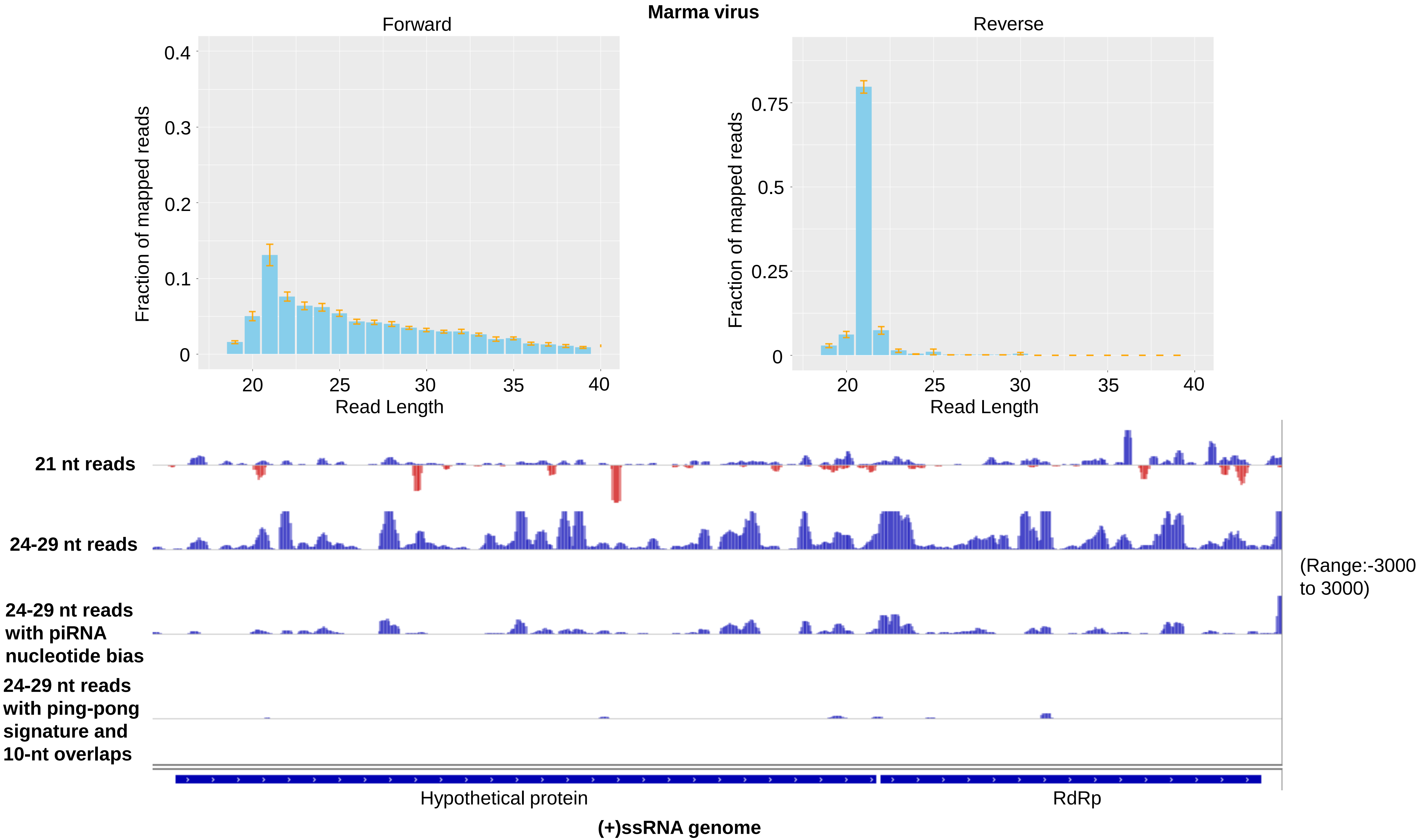
Marma virus
Forward
Reverse
Fraction of mapped reads
Read Length
0.4
0.3
0.2
0.1
0
0.75
0.5
0.25
20
25
30
35
40
21 nt reads
24-29 nt reads
24-29 nt reads with piRNA nucleotide bias
24-29 nt reads with ping-pong signature and 10-nt overlaps
(Range:-3000 to 3000)
Hypothetical protein
RdRp
(+)ssRNA genome

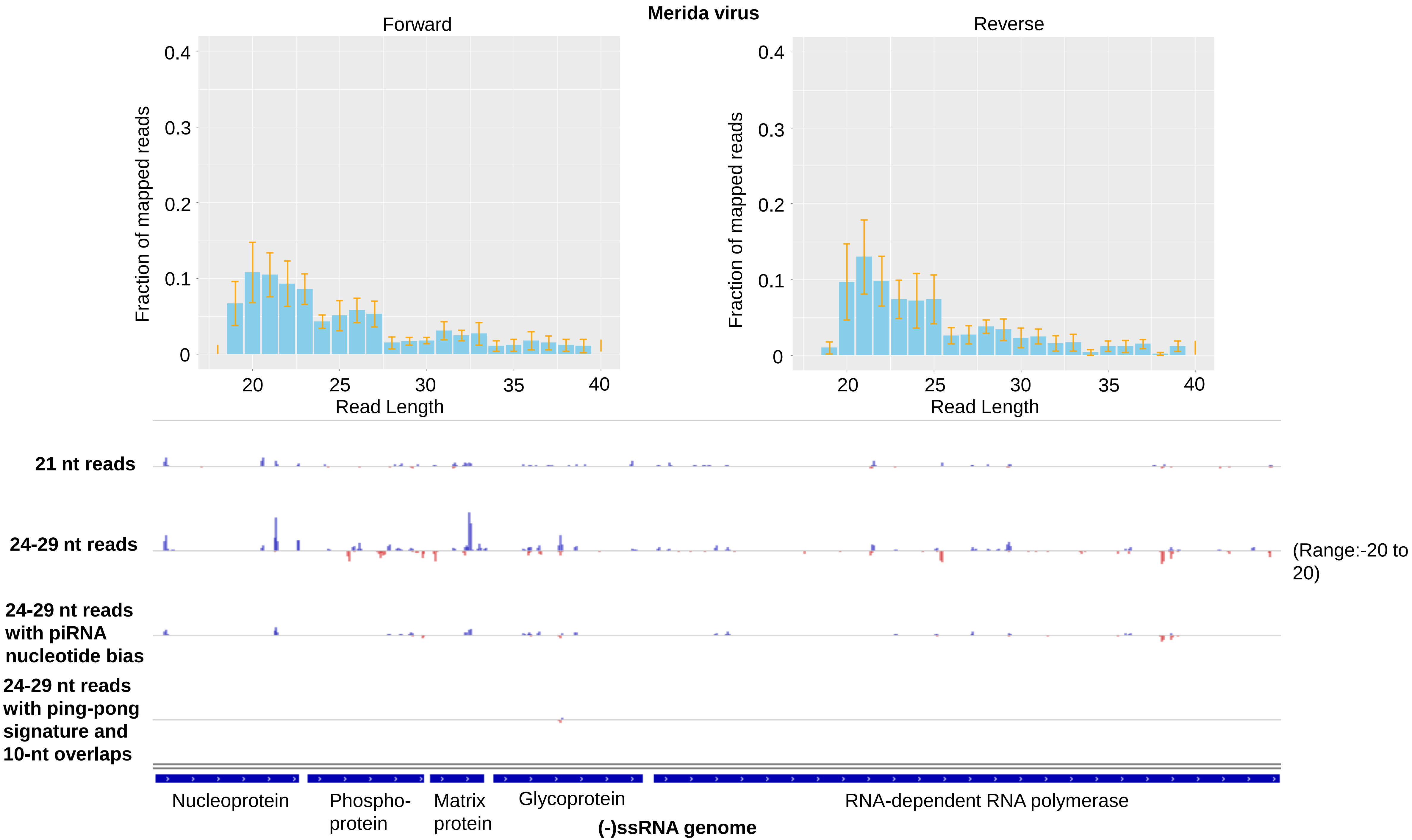

Merida virus
Forward
Reverse
Fraction of mapped reads
0.4
0.3
0.2
0.1
0
20
25
30
35
40
Read Length
21 nt reads
24-29 nt reads
24-29 nt reads with piRNA nucleotide bias
24-29 nt reads with ping-pong signature and 10-nt overlaps
(Range:-20 to 20)
Nucleoprotein
Phospho-protein
Matrix protein
Glycoprotein
RNA-dependent RNA polymerase
(-)ssRNA genome

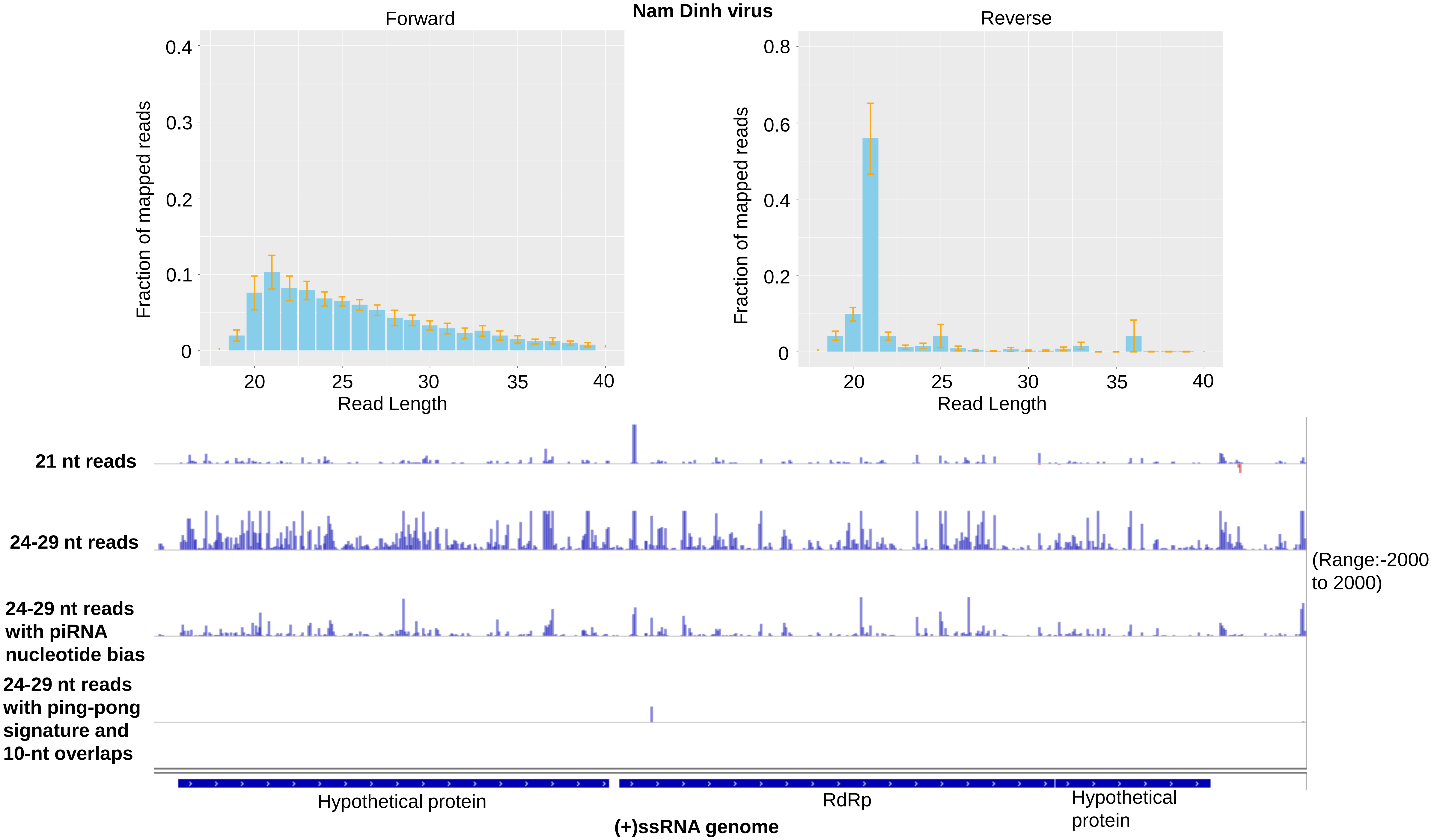
Nam Dinh virus
Forward
Reverse
Fraction of mapped reads
Read Length
21 nt reads
24-29 nt reads
24-29 nt reads with piRNA nucleotide bias
24-29 nt reads with ping-pong signature and 10-nt overlaps
(Range:-2000 to 2000)
Hypothetical protein
RdRp
Hypothetical protein
(+)ssRNA genome

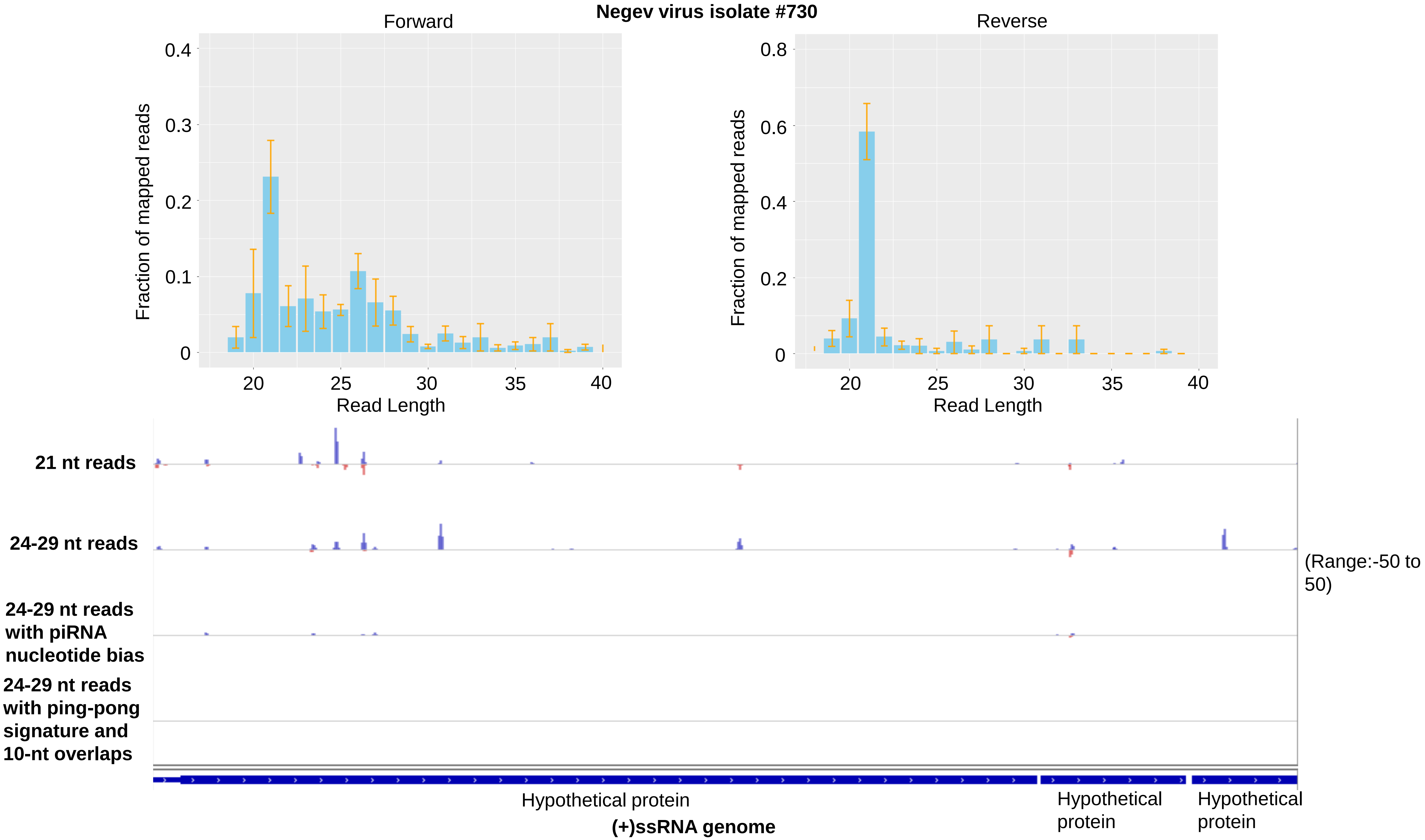

Negev virus isolate #730
Forward
Reverse
Fraction of mapped reads
0.4
0.3
0.2
0.1
0
0.8
0.6
0.4
0.2
0
20
25
30
35
40
Read Length
21 nt reads
24-29 nt reads
24-29 nt reads with piRNA nucleotide bias
24-29 nt reads with ping-pong signature and 10-nt overlaps
(Range:-50 to 50)
Hypothetical protein
Hypothetical protein
Hypothetical protein
(+)ssRNA genome

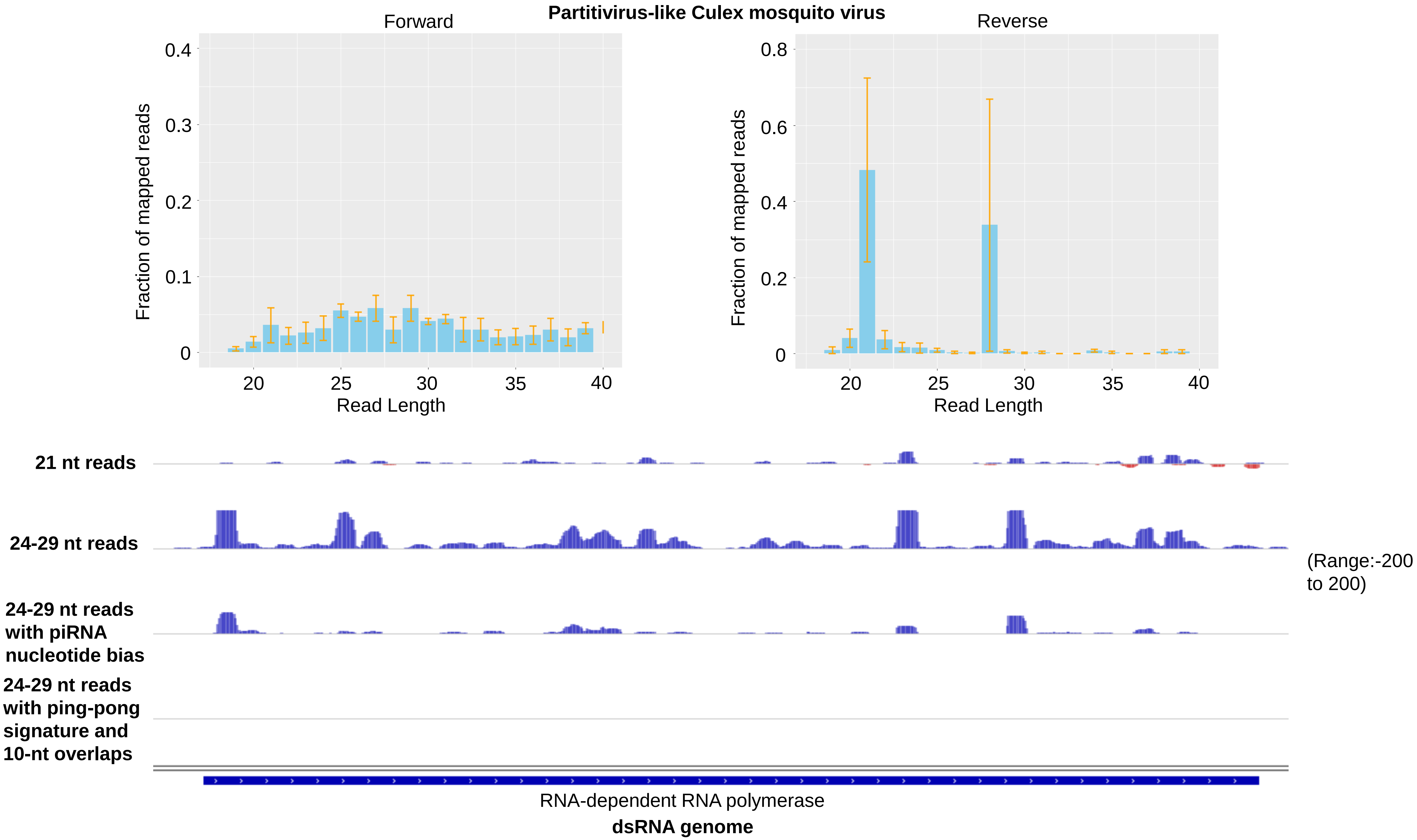

Partitivirus-like Culex mosquito virus
Forward
Reverse
Fraction of mapped reads
Read Length
0
0.1
0.2
0.3
0.4
0.6
0.8
20
25
30
35
40
21 nt reads
24-29 nt reads
24-29 nt reads with piRNA nucleotide bias
24-29 nt reads with ping-pong signature and 10-nt overlaps
(Range:-200 to 200)
RNA-dependent RNA polymerase
dsRNA genome

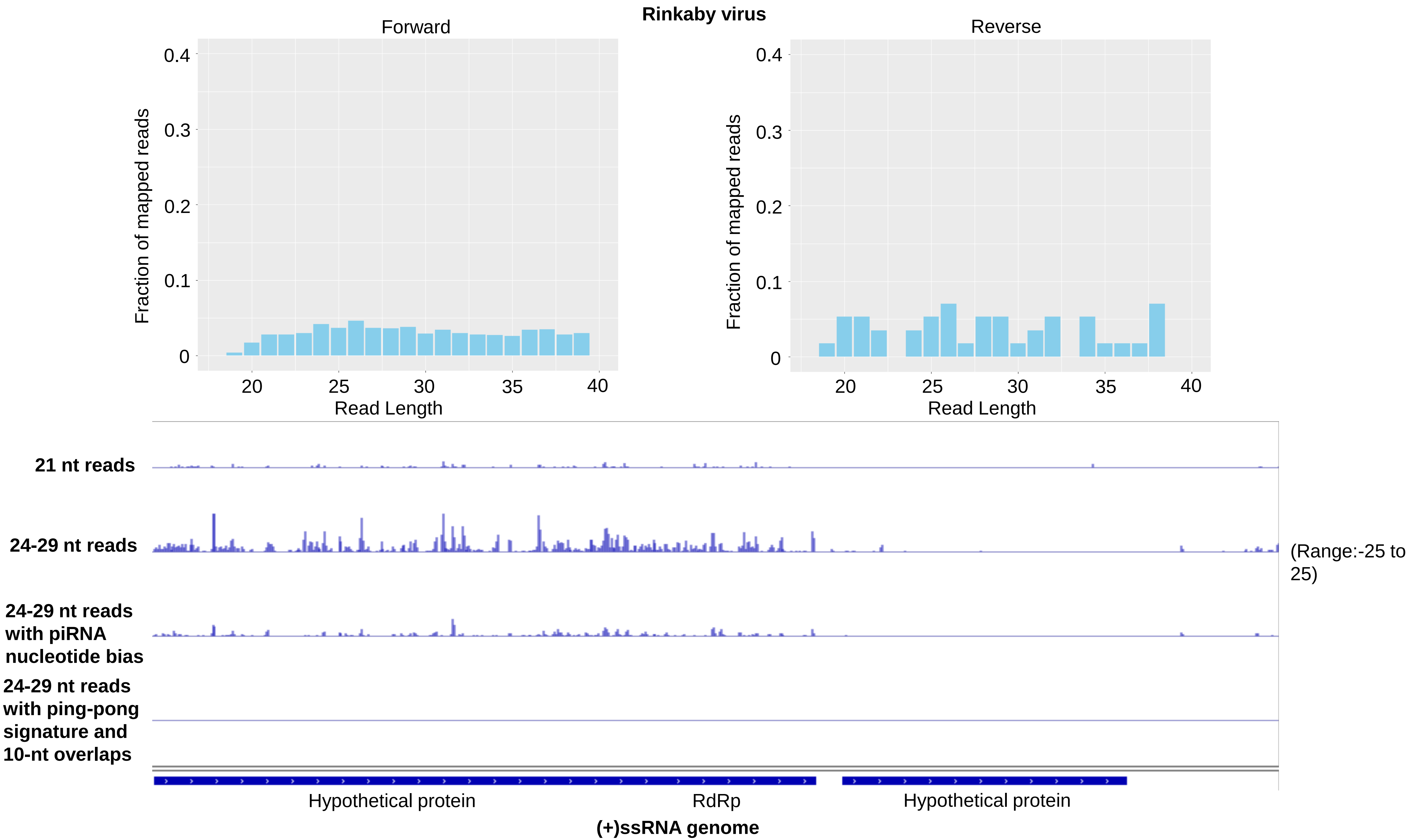

Rinkaby virus
Forward
Reverse
Fraction of mapped reads
0.4
0.3
0.2
0.1
0
20
25
30
35
40
Read Length
21 nt reads
24-29 nt reads
24-29 nt reads with piRNA nucleotide bias
24-29 nt reads with ping-pong signature and 10-nt overlaps
(Range:-25 to 25)
Hypothetical protein
RdRp
Hypothetical protein
(+)ssRNA genome

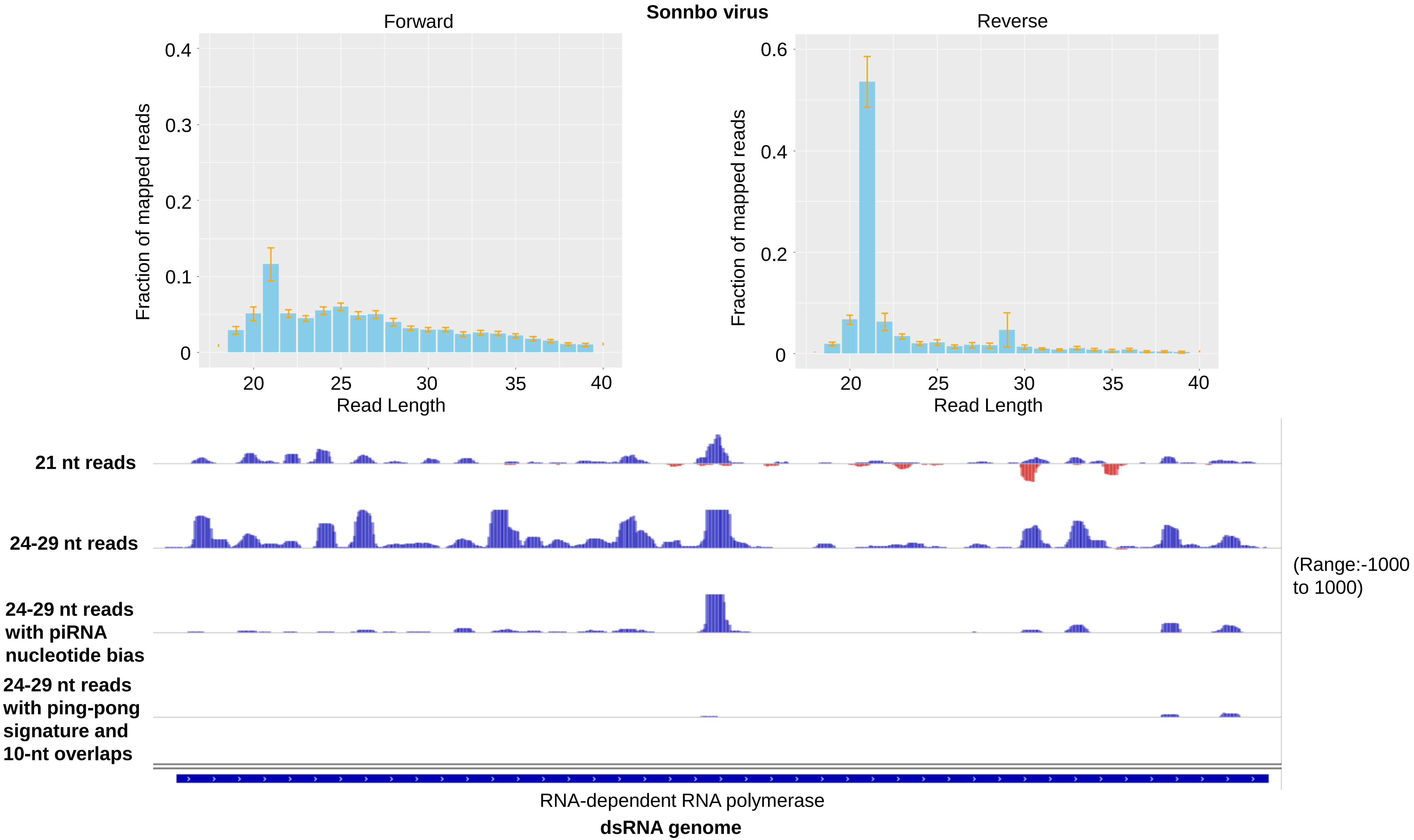

Sonnbo virus
Forward
Reverse
Fraction of mapped reads
Read Length
0
0.1
0.2
0.3
0.4
0.6
20
25
30
35
40
21 nt reads
24-29 nt reads
24-29 nt reads with piRNA nucleotide bias
24-29 nt reads with ping-pong signature and 10-nt overlaps
(Range:-1000 to 1000)
RNA-dependent RNA polymerase
dsRNA genome

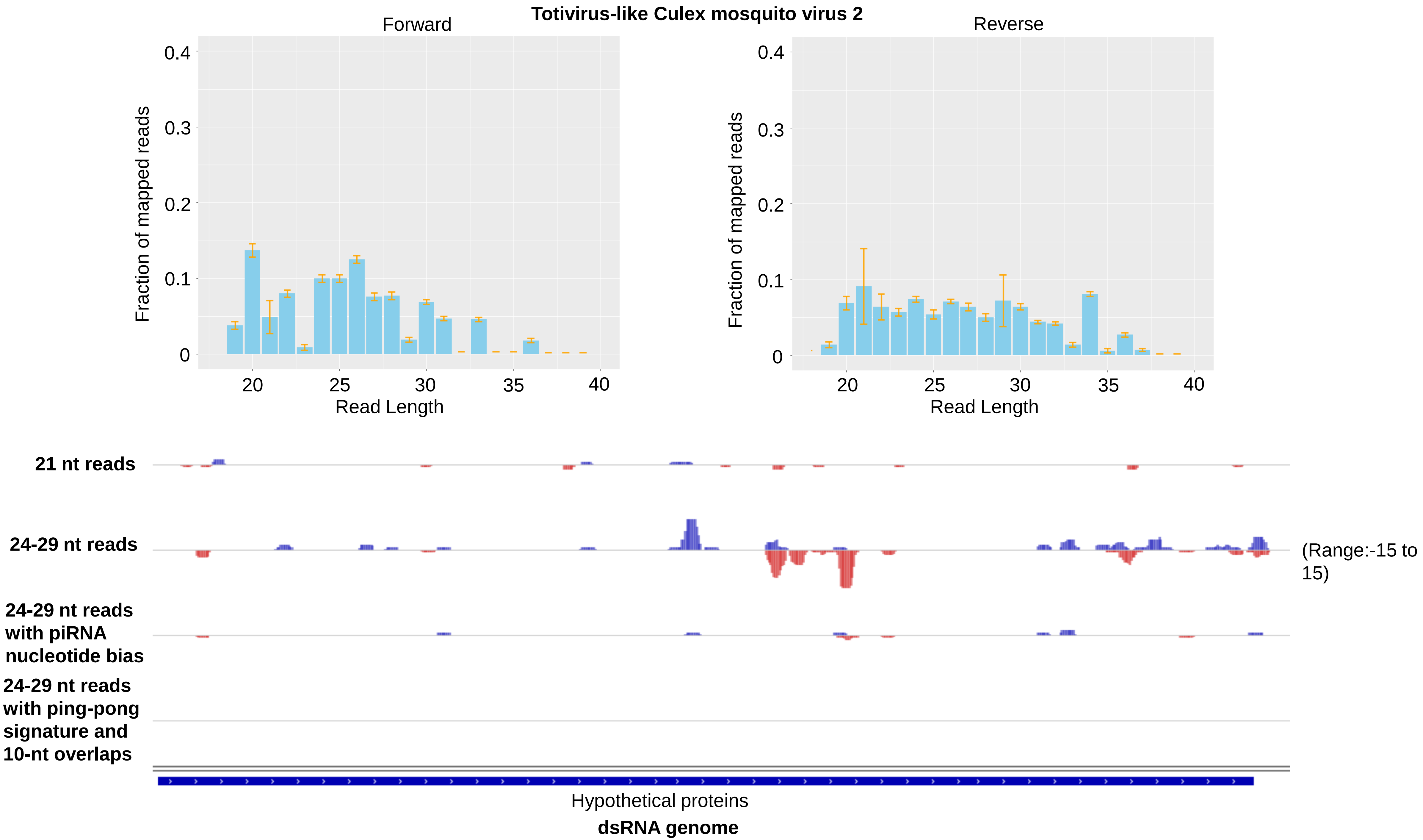
Totivirus-like Culex mosquito virus 2
Forward
Reverse
Fraction of mapped reads
0.4
0.3
0.2
0.1
0
20
25
30
35
40
Read Length
21 nt reads
24-29 nt reads
(Range:-15 to 15)
24-29 nt reads with piRNA nucleotide bias
24-29 nt reads with ping-pong signature and 10-nt overlaps
Hypothetical proteins
dsRNA genome

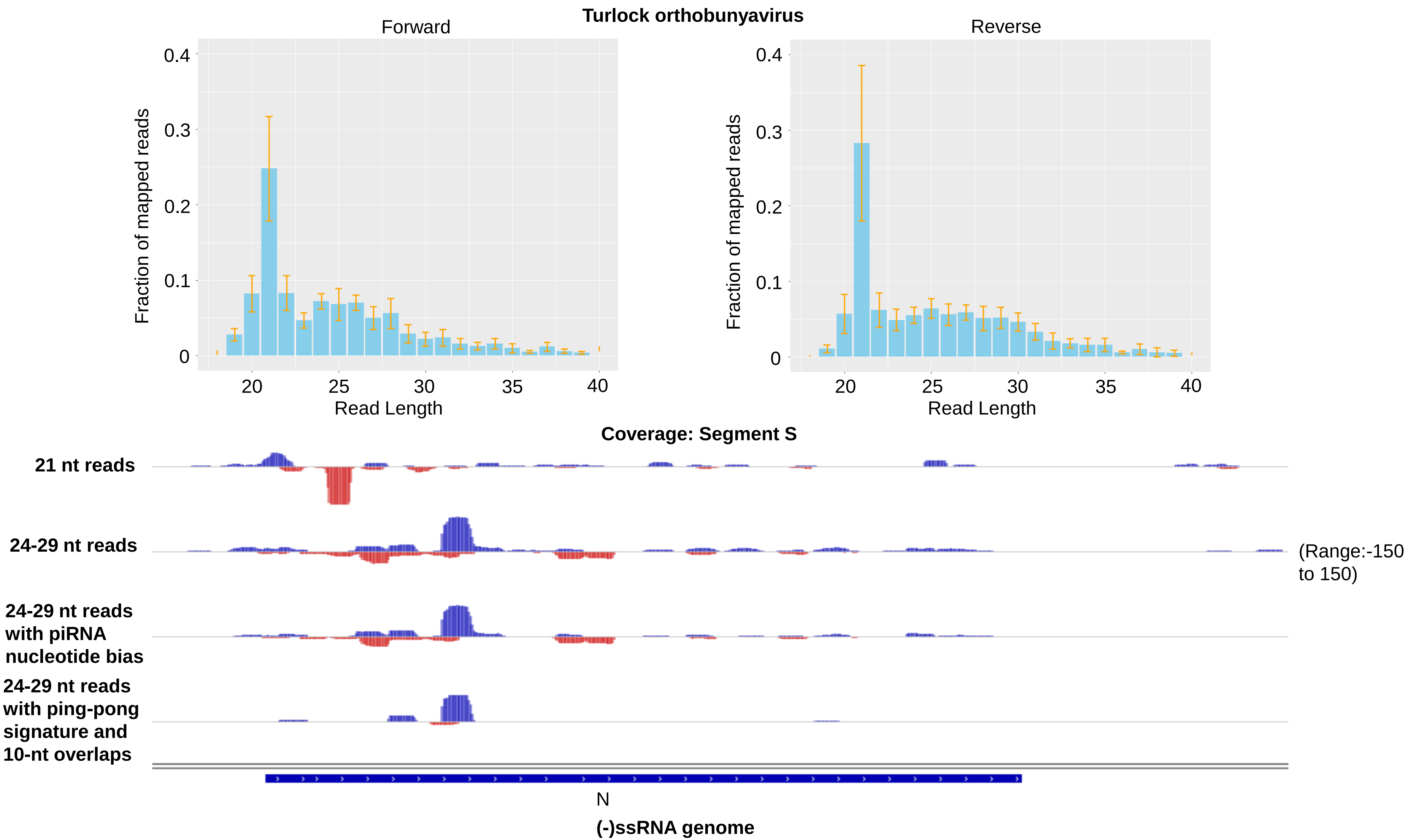

Turlock orthobunyavirus
Forward
Reverse
Fraction of mapped reads
0.4
0.3
0.2
0.1
0
20
25
30
35
40
Read Length
Coverage: Segment S
21 nt reads
24-29 nt reads
24-29 nt reads with piRNA nucleotide bias
24-29 nt reads with ping-pong signature and 10-nt overlaps
(Range:-150 to 150)
N
(-)ssRNA genome

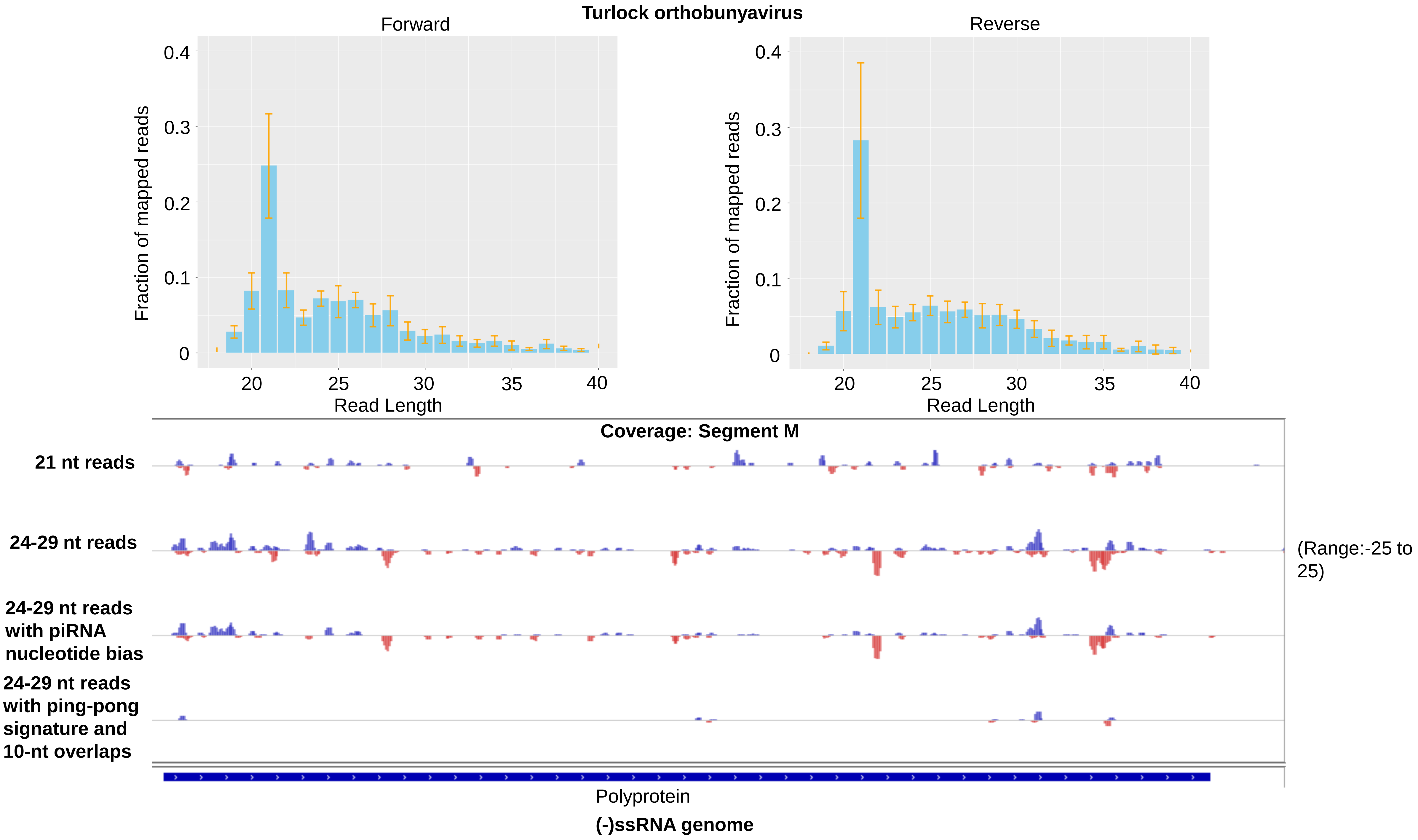

Turlock orthobunyavirus
Forward
Reverse
Fraction of mapped reads
0.4
0.3
0.2
0.1
0
20
25
30
35
40
Read Length
Coverage: Segment M
21 nt reads
24-29 nt reads
24-29 nt reads with piRNA nucleotide bias
24-29 nt reads with ping-pong signature and 10-nt overlaps
(Range:-25 to 25)
Polyprotein
(-)ssRNA genome

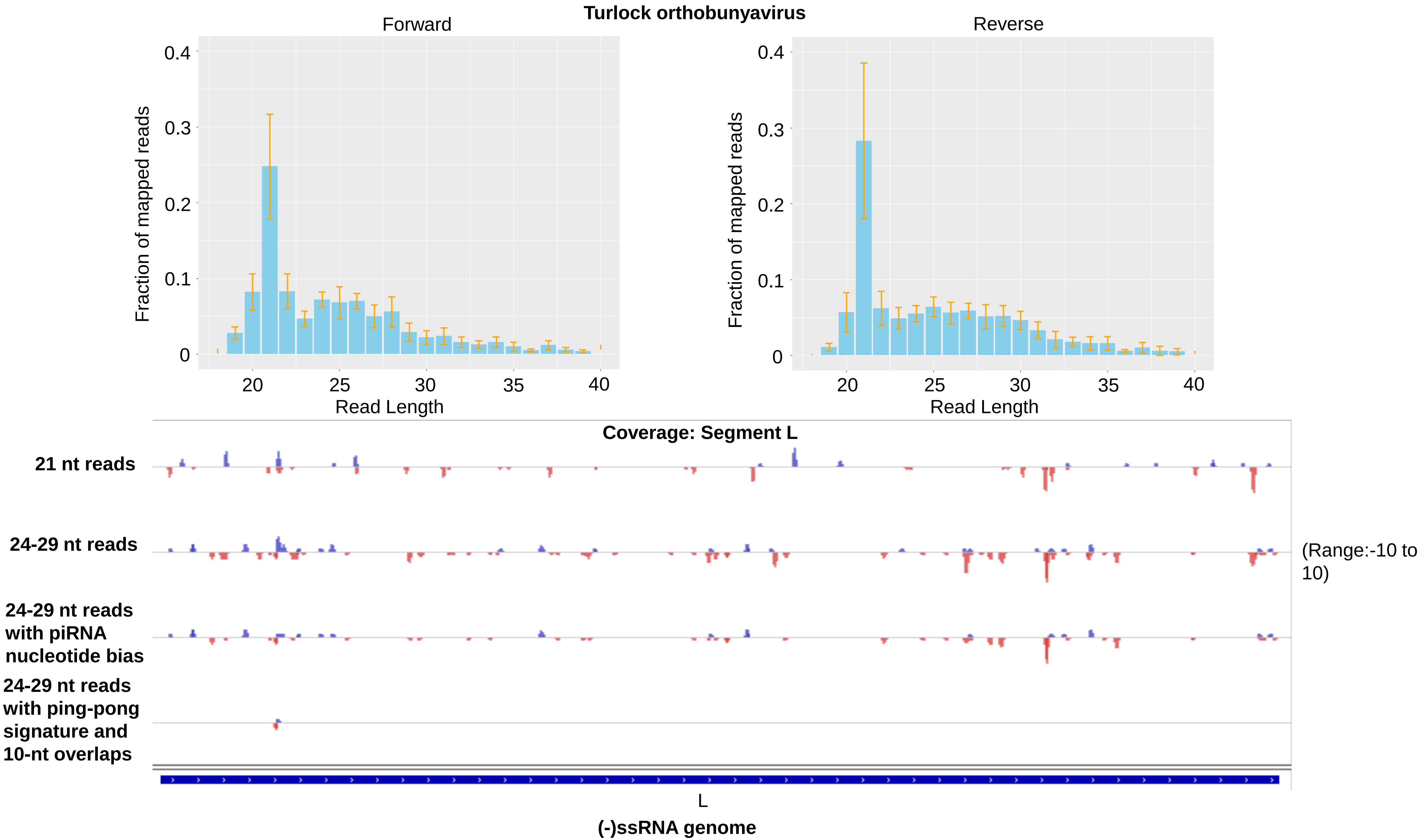

Turlock orthobunyavirus
Forward
Reverse
Fraction of mapped reads
Read Length
Coverage: Segment L
21 nt reads
24-29 nt reads
24-29 nt reads with piRNA nucleotide bias
24-29 nt reads with ping-pong signature and 10-nt overlaps
(Range:-10 to 10)
L
(-)ssRNA genome

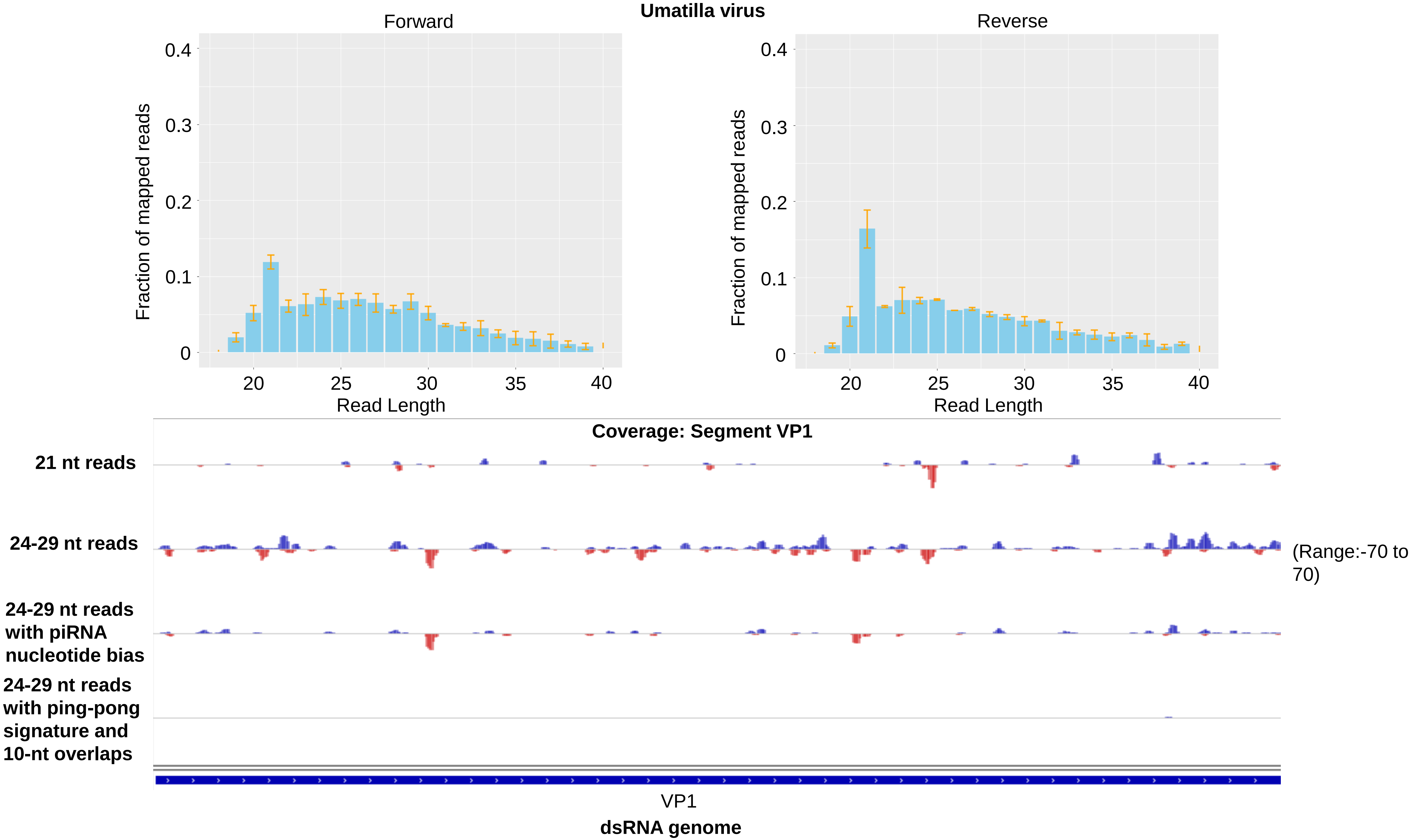

Umatilla virus
Forward
Reverse
Fraction of mapped reads
0.4
0.3
0.2
0.1
0
20
25
30
35
40
Read Length
Coverage: Segment VP1
21 nt reads
24-29 nt reads
24-29 nt reads with piRNA nucleotide bias
24-29 nt reads with ping-pong signature and 10-nt overlaps
(Range:-70 to 70)
VP1
dsRNA genome

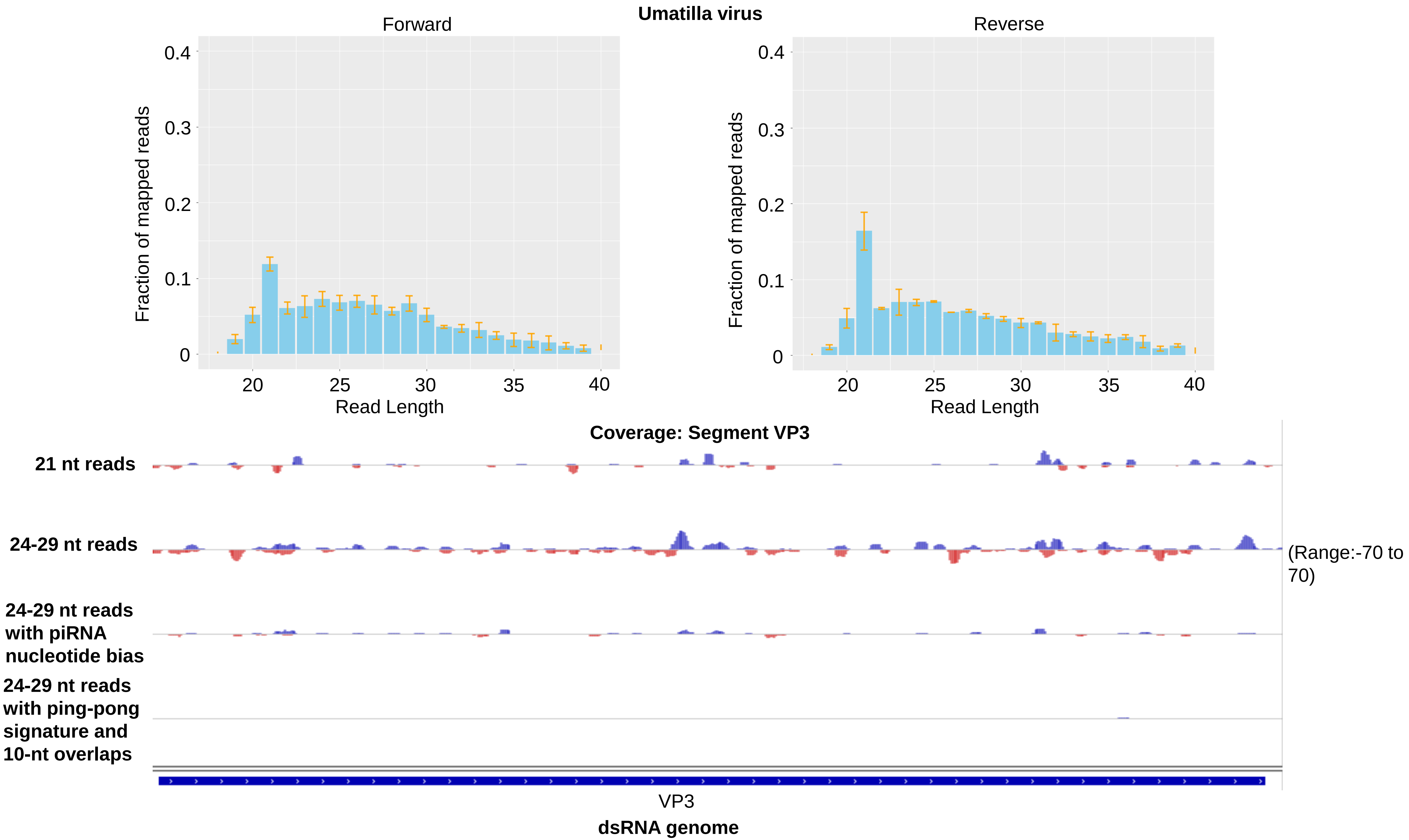
Umatilla virus
Forward
Reverse
Fraction of mapped reads
0.4
0.3
0.2
0.1
0
20
25
30
35
40
Read Length
Coverage: Segment VP3
21 nt reads
24-29 nt reads
24-29 nt reads with piRNA nucleotide bias
24-29 nt reads with ping-pong signature and 10-nt overlaps
(Range:-70 to 70)
VP3
dsRNA genome

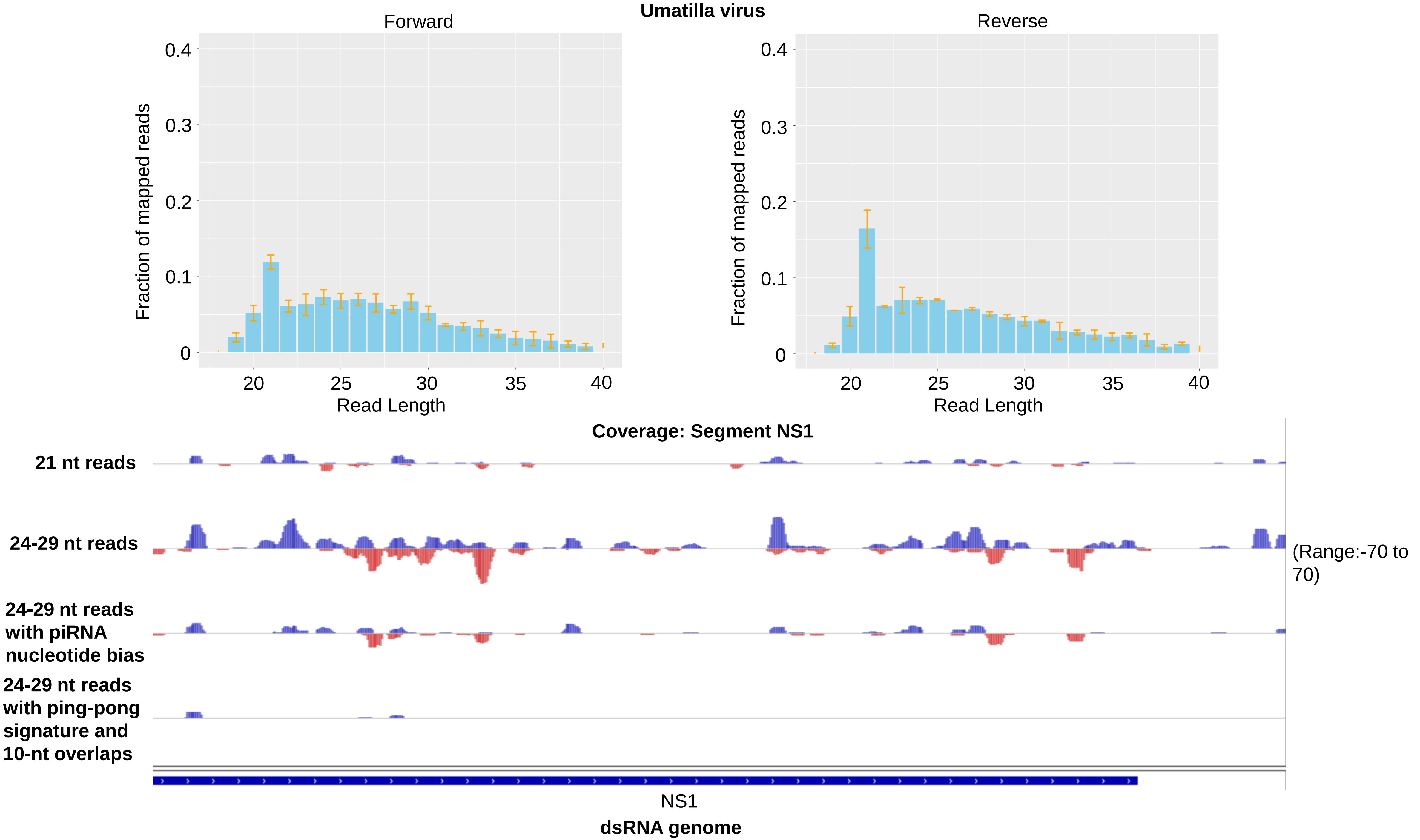
Umatilla virus
Forward
Reverse
Fraction of mapped reads
0.4
0.3
0.2
0.1
0
20
25
30
35
40
Read Length
Coverage: Segment NS1
21 nt reads
24-29 nt reads
24-29 nt reads with piRNA nucleotide bias
24-29 nt reads with ping-pong signature and 10-nt overlaps
(Range:-70 to 70)
NS1
dsRNA genome

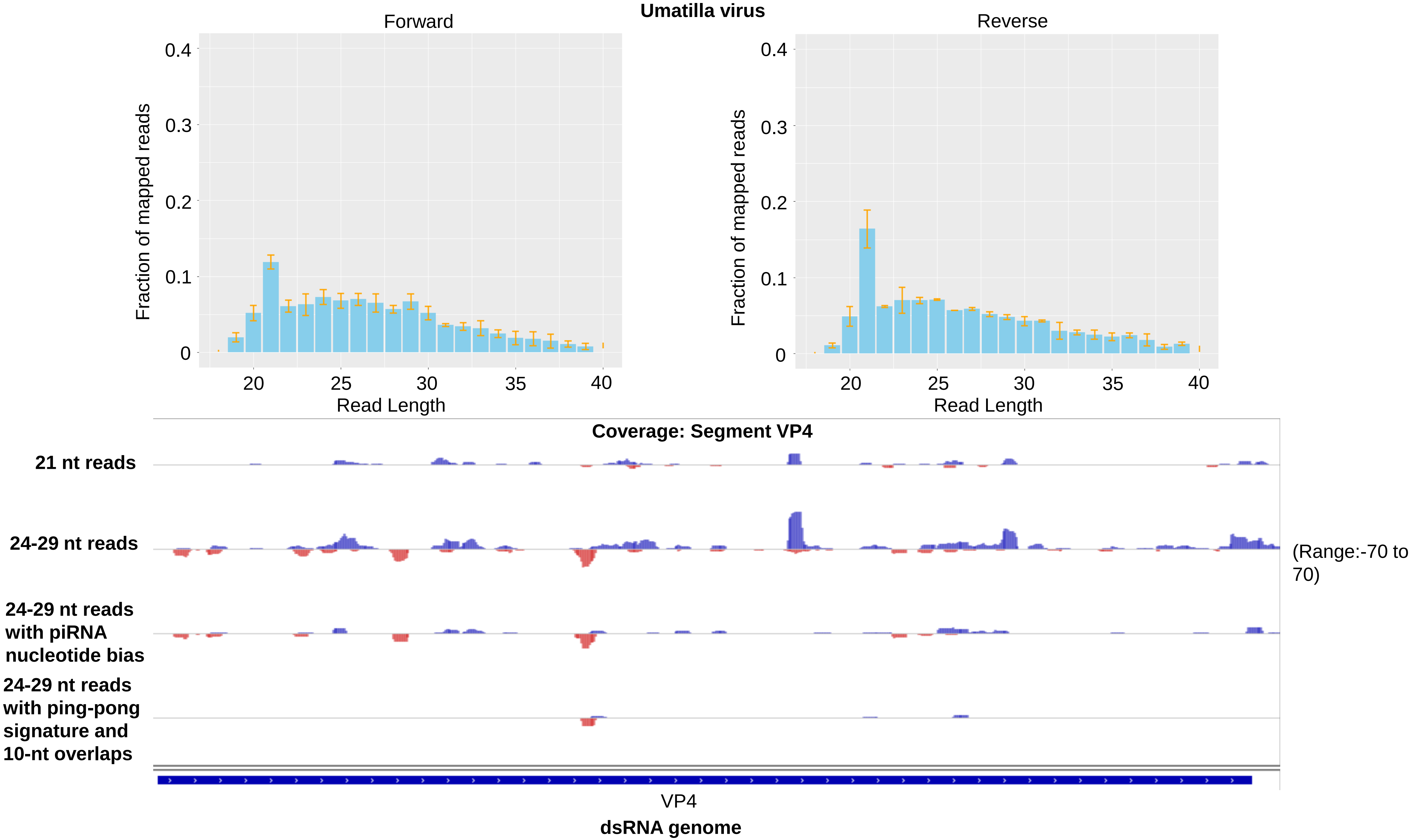
Umatilla virus
Forward
Reverse
Fraction of mapped reads
0.4
0.3
0.2
0.1
0
20
25
30
35
40
Read Length
Coverage: Segment VP4
21 nt reads
24-29 nt reads
24-29 nt reads with piRNA nucleotide bias
24-29 nt reads with ping-pong signature and 10-nt overlaps
(Range:-70 to 70)
VP4
dsRNA genome

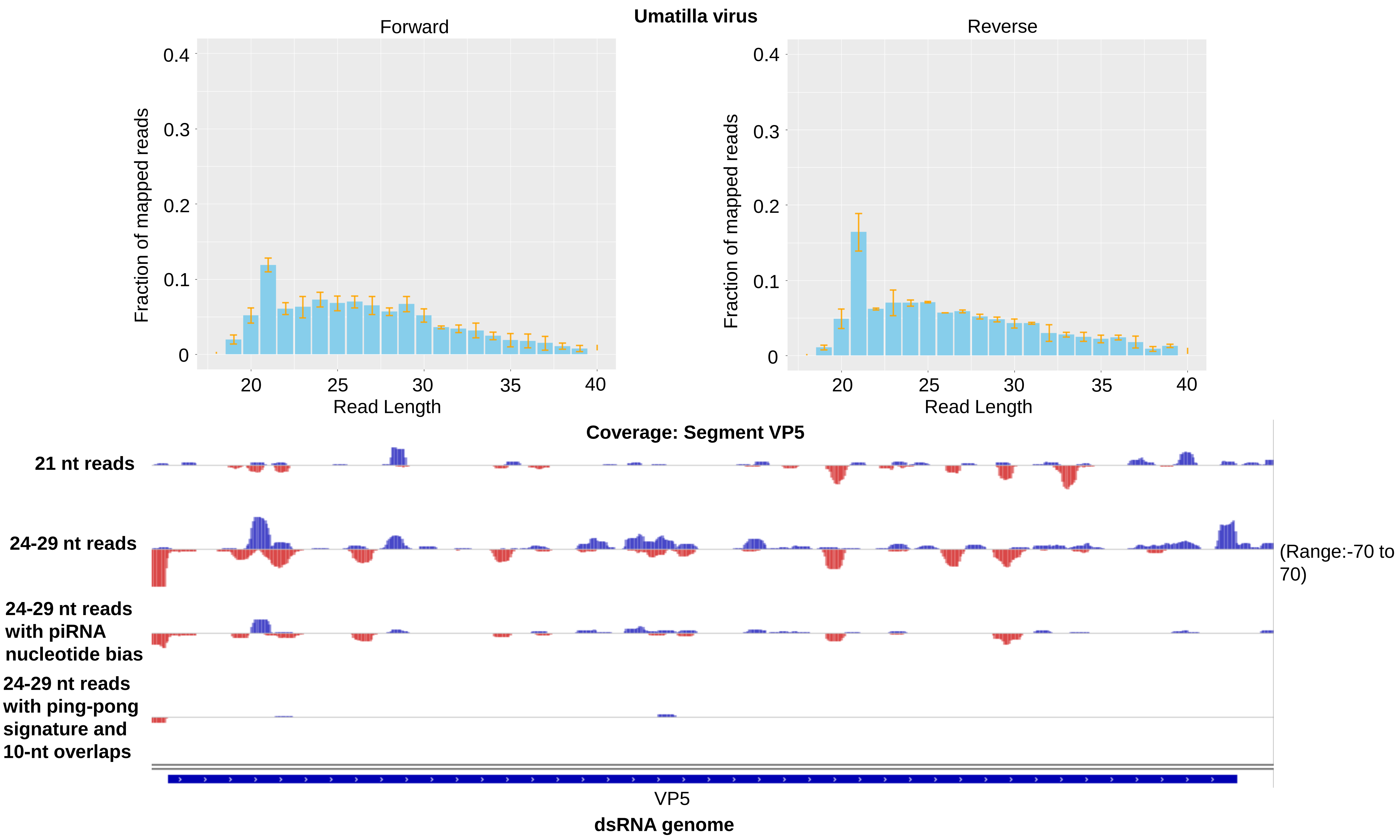

Umatilla virus
Forward
Reverse
Fraction of mapped reads
0.4
0.3
0.2
0.1
0
20
25
30
35
40
Read Length
Coverage: Segment VP5
21 nt reads
24-29 nt reads
24-29 nt reads with piRNA nucleotide bias
24-29 nt reads with ping-pong signature and 10-nt overlaps
(Range:-70 to 70)
VP5
dsRNA genome

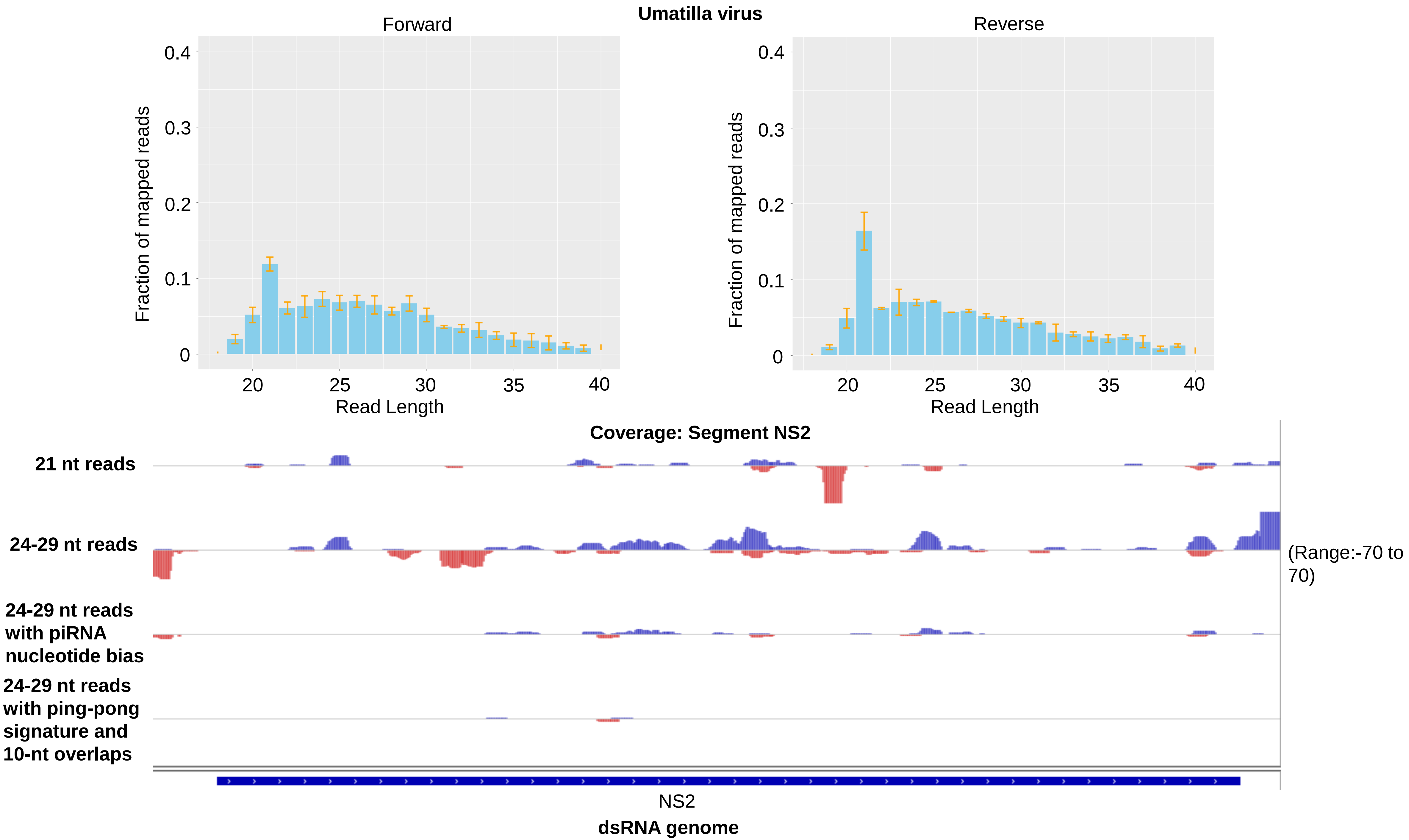

Umatilla virus
Forward
Reverse
Fraction of mapped reads
0.4
0.3
0.2
0.1
0
20
25
30
35
40
Read Length
Coverage: Segment NS2
21 nt reads
24-29 nt reads
24-29 nt reads with piRNA nucleotide bias
24-29 nt reads with ping-pong signature and 10-nt overlaps
(Range:-70 to 70)
NS2
dsRNA genome

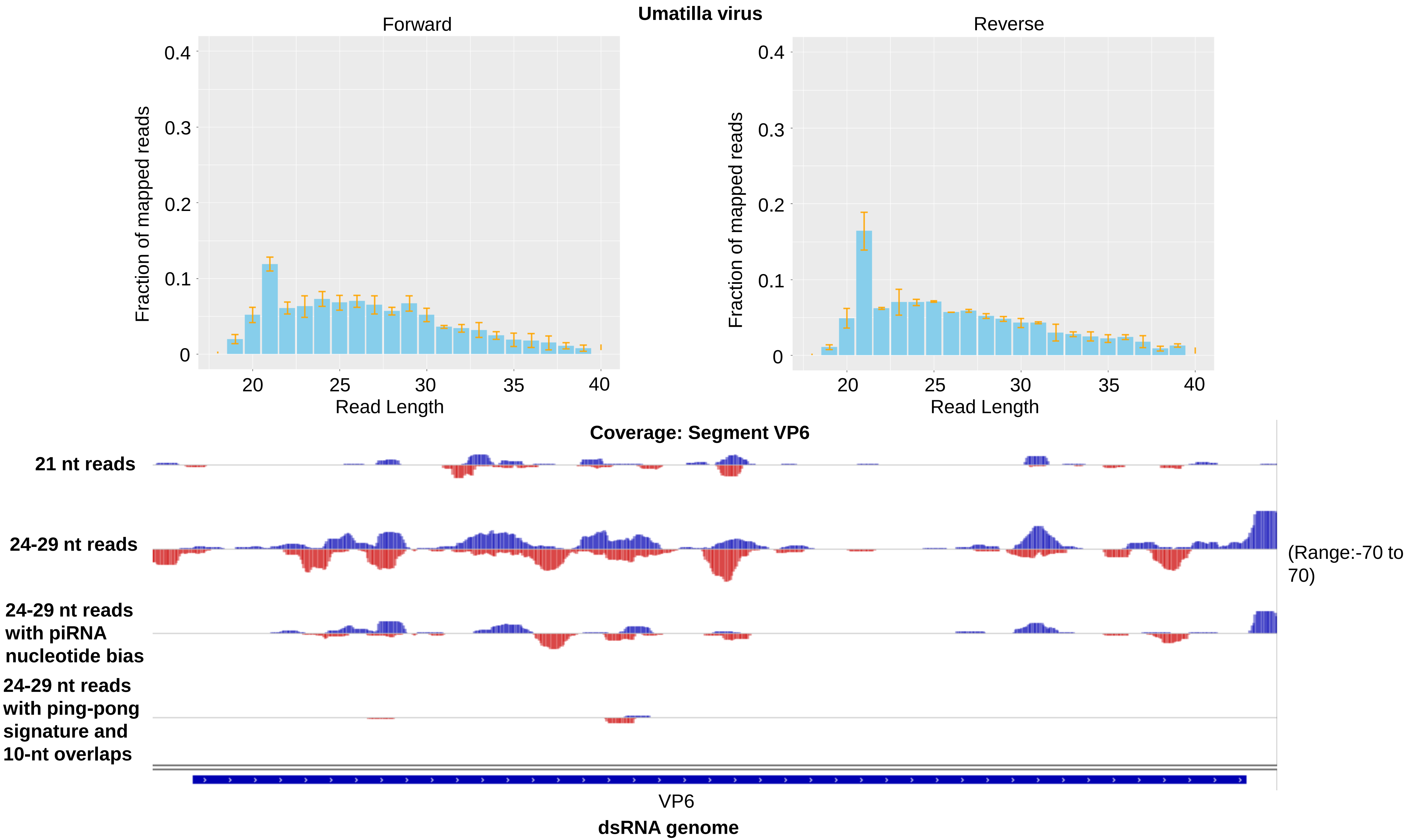
Umatilla virus
Forward
Reverse
Fraction of mapped reads
0.4
0.3
0.2
0.1
0
20
25
30
35
40
Read Length
Coverage: Segment VP6
21 nt reads
24-29 nt reads
24-29 nt reads with piRNA nucleotide bias
24-29 nt reads with ping-pong signature and 10-nt overlaps
(Range:-70 to 70)
VP6
dsRNA genome

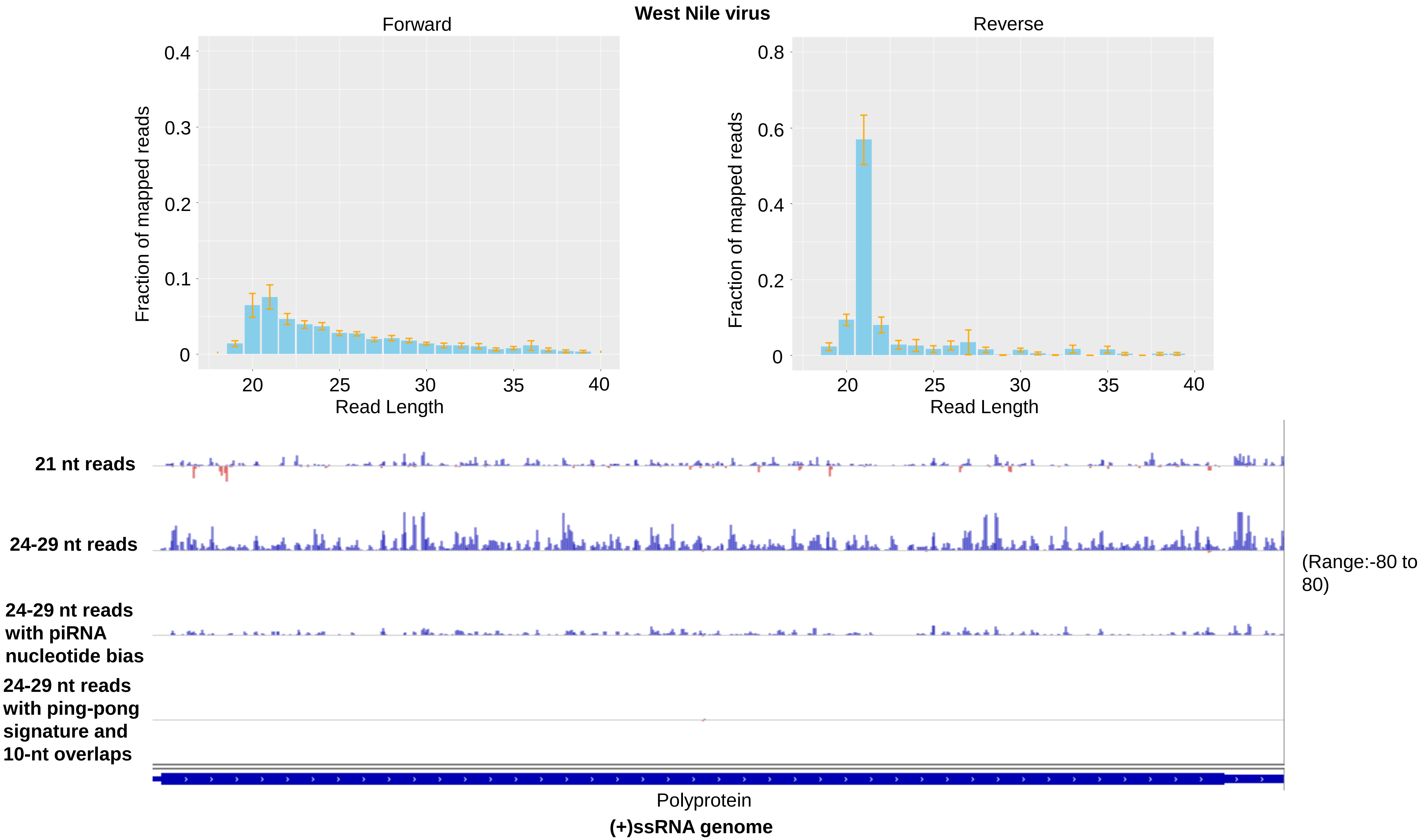
West Nile virus
Forward
Reverse
Fraction of mapped reads
Read Length
0.4
0.3
0.2
0.1
0
0.8
0.6
0.4
0.2
20
25
30
35
40
21 nt reads
24-29 nt reads
24-29 nt reads with piRNA nucleotide bias
24-29 nt reads with ping-pong signature and 10-nt overlaps
(Range:-80 to 80)
Polyprotein
(+)ssRNA genome

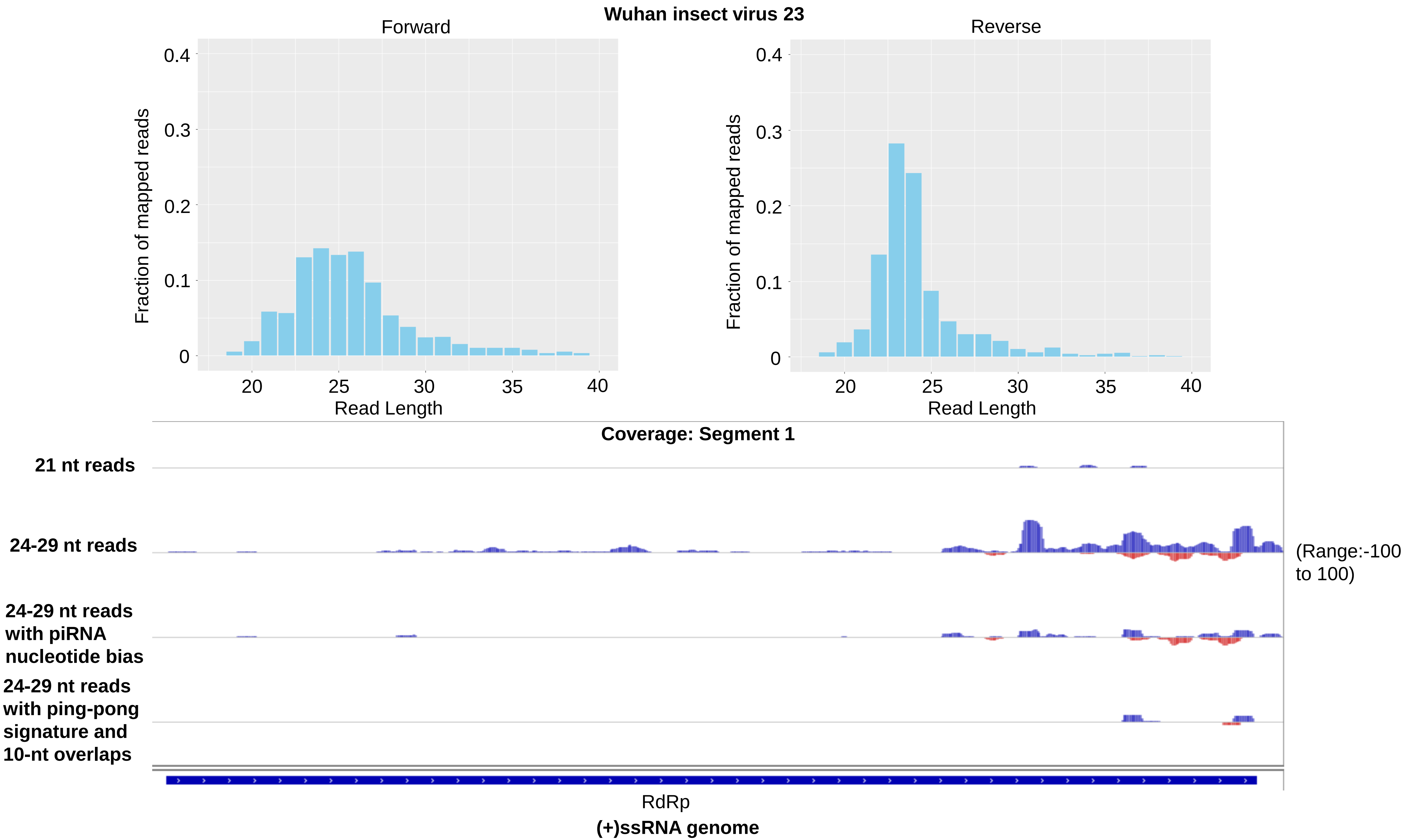

Wuhan insect virus 23
Forward
Reverse
Fraction of mapped reads
0.4
0.3
0.2
0.1
0
20
25
30
35
40
Read Length
Coverage: Segment 1
21 nt reads
24-29 nt reads
24-29 nt reads with piRNA nucleotide bias
24-29 nt reads with ping-pong signature and 10-nt overlaps
(Range:-100 to 100)
RdRp
(+)ssRNA genome

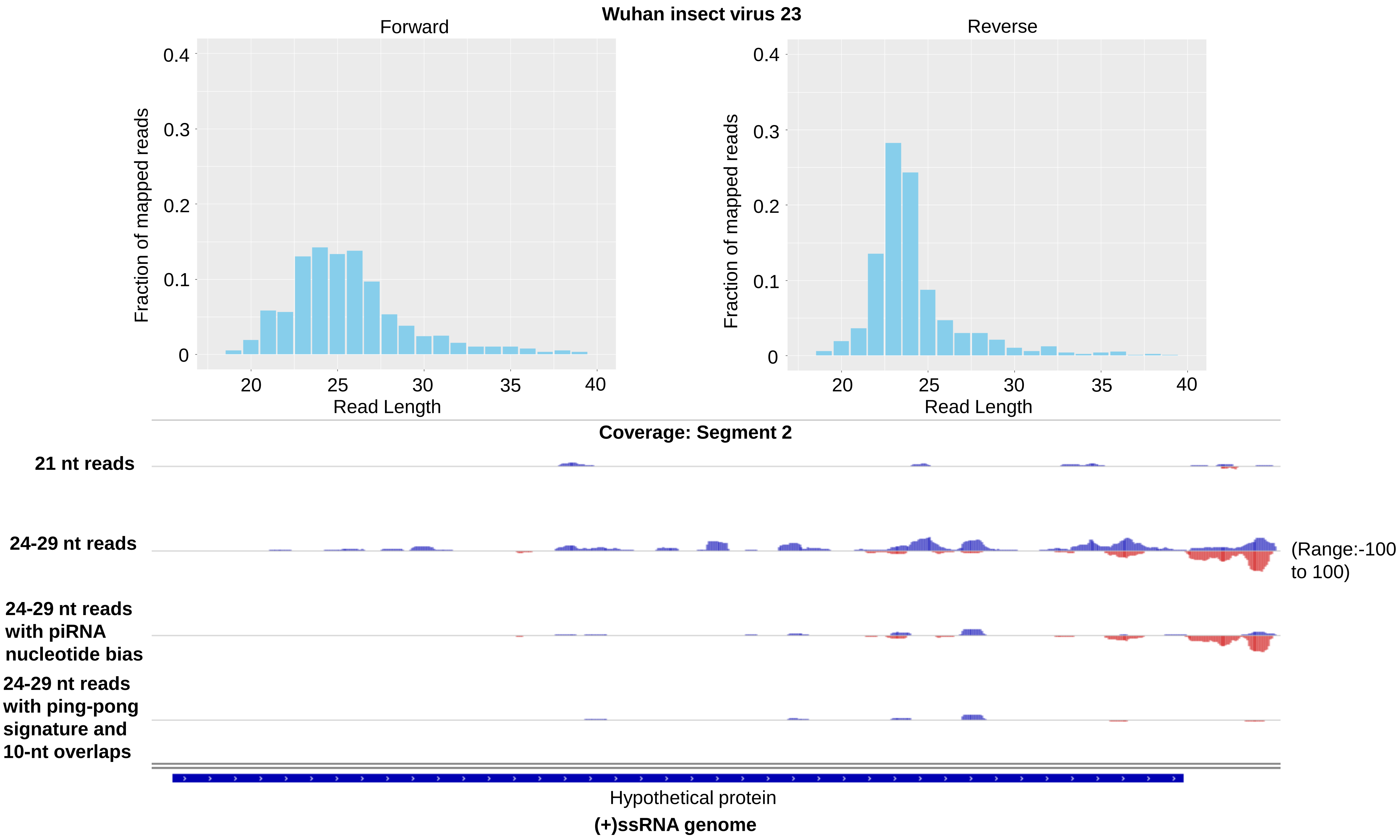

Wuhan insect virus 23
Forward
Reverse
Fraction of mapped reads
0.4
0.3
0.2
0.1
0
20
25
30
35
40
Read Length
Coverage: Segment 2
21 nt reads
24-29 nt reads
(Range:-100 to 100)
24-29 nt reads with piRNA nucleotide bias
24-29 nt reads with ping-pong signature and 10-nt overlaps
Hypothetical protein
(+)ssRNA genome

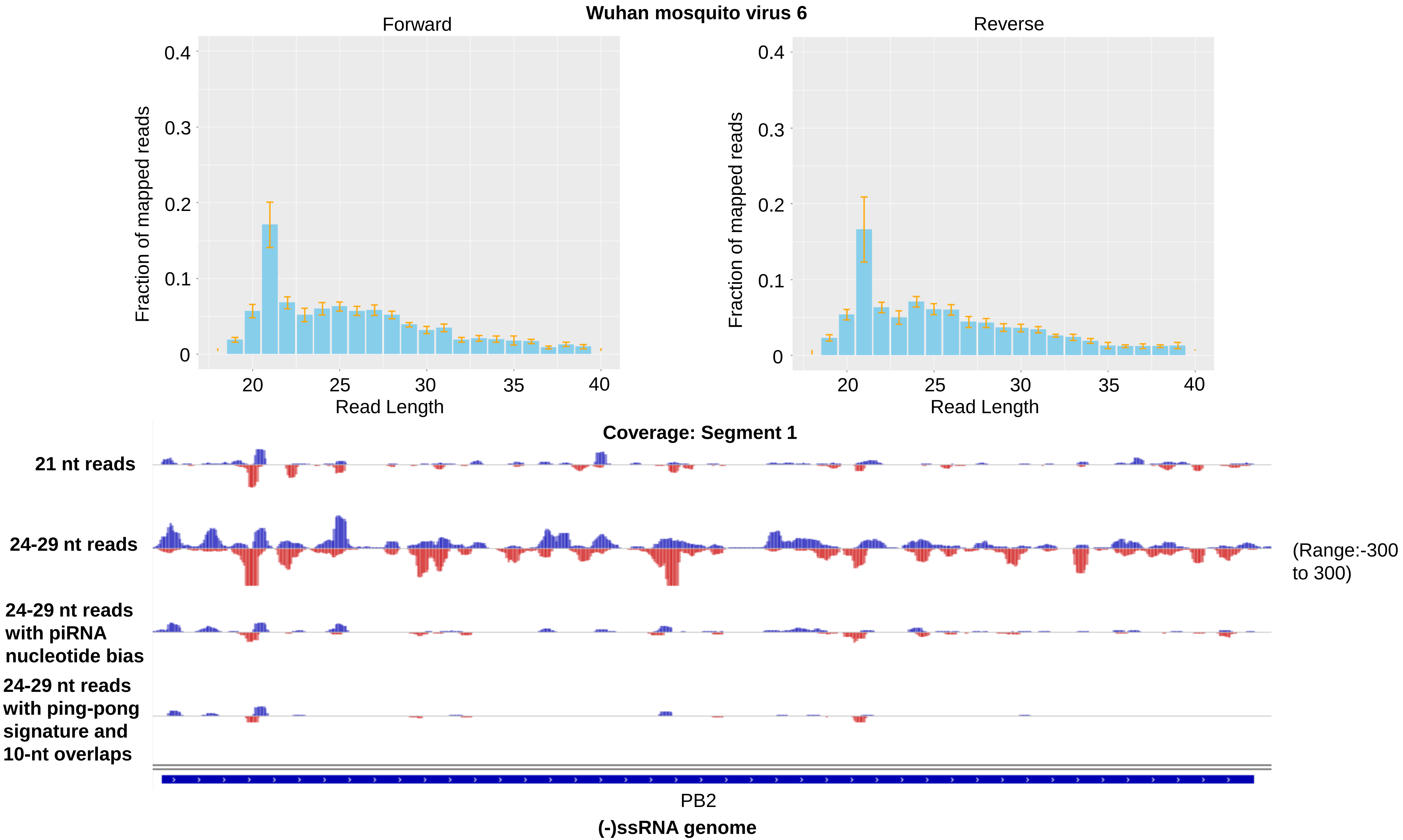

Wuhan mosquito virus 6
Forward
Reverse
Fraction of mapped reads
0.4
0.3
0.2
0.1
0
20
25
30
35
40
Read Length
Coverage: Segment 1
21 nt reads
24-29 nt reads
24-29 nt reads with piRNA nucleotide bias
24-29 nt reads with ping-pong signature and 10-nt overlaps
(Range:-300 to 300)
PB2
(-)ssRNA genome

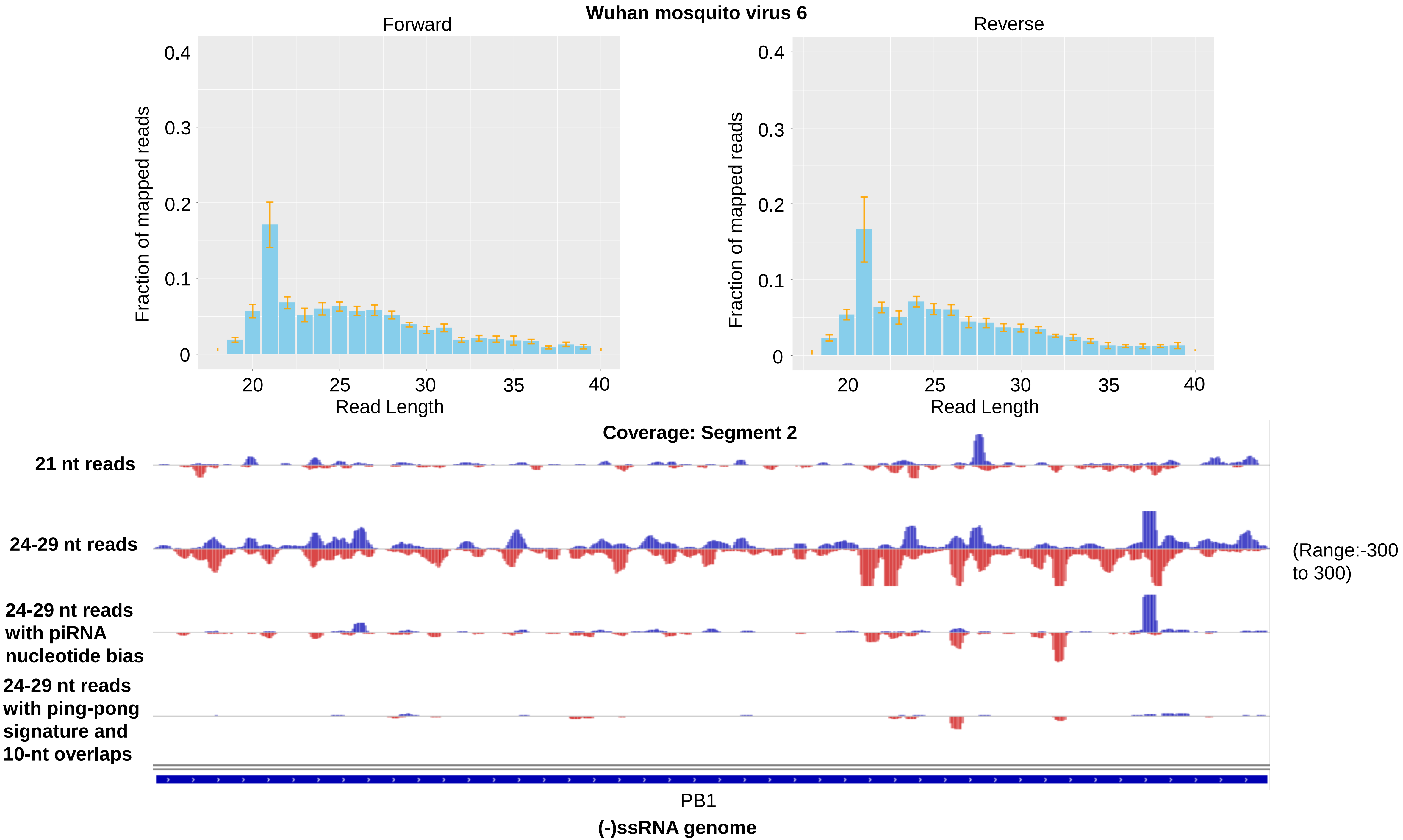

Wuhan mosquito virus 6
Forward
Reverse
Fraction of mapped reads
0.4
0.3
0.2
0.1
0
20
25
30
35
40
Read Length
Coverage: Segment 2
21 nt reads
24-29 nt reads
24-29 nt reads with piRNA nucleotide bias
24-29 nt reads with ping-pong signature and 10-nt overlaps
(Range:-300 to 300)
PB1
(-)ssRNA genome

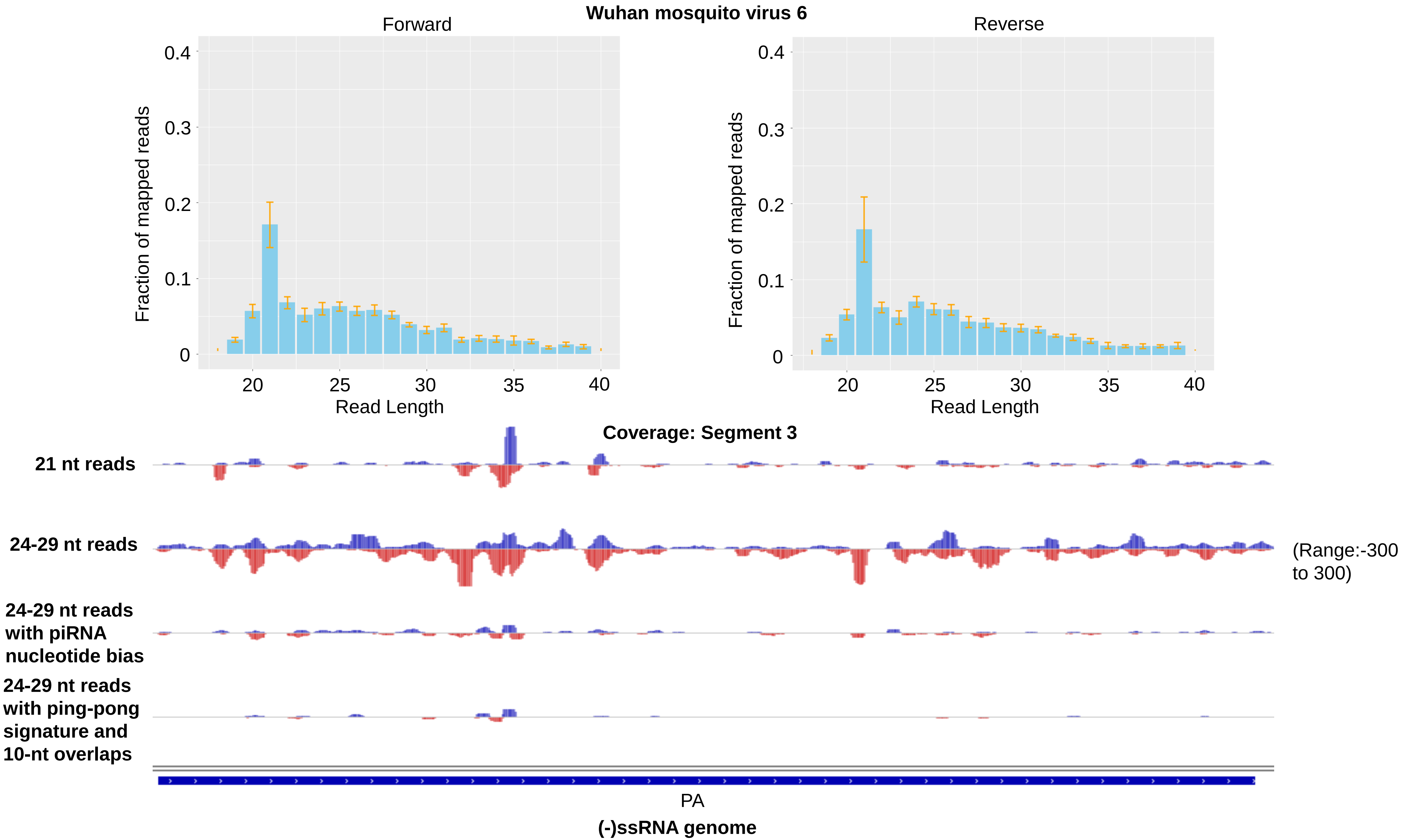
Wuhan mosquito virus 6
Forward
Reverse
Fraction of mapped reads
0.4
0.3
0.2
0.1
0
20
25
30
35
40
Read Length
Coverage: Segment 3
21 nt reads
24-29 nt reads
24-29 nt reads with piRNA nucleotide bias
24-29 nt reads with ping-pong signature and 10-nt overlaps
(Range:-300 to 300)
PA
(-)ssRNA genome

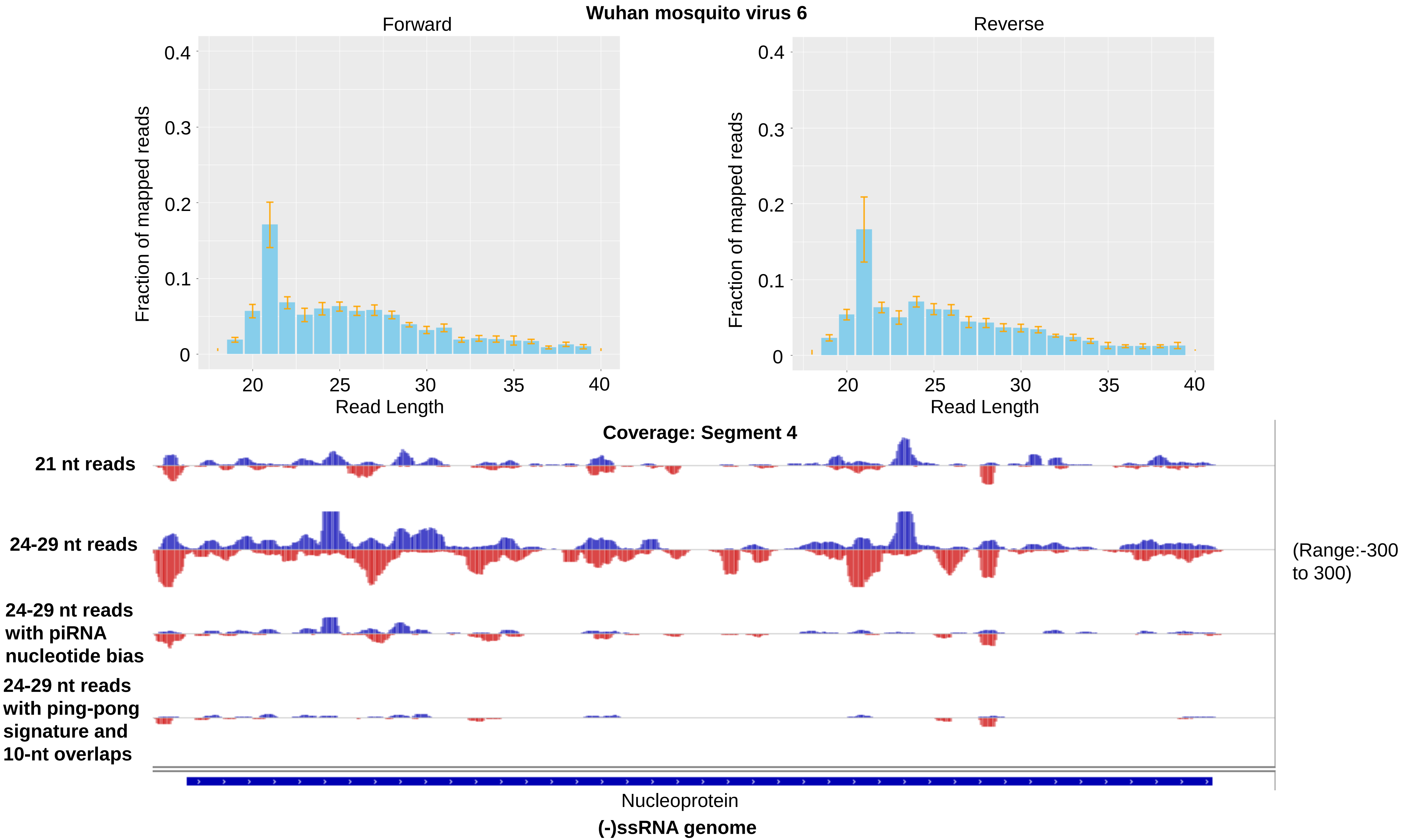

Wuhan mosquito virus 6
Forward
Reverse
Fraction of mapped reads
0.4
0.3
0.2
0.1
0
20
25
30
35
40
Read Length
Coverage: Segment 4
21 nt reads
24-29 nt reads
24-29 nt reads with piRNA nucleotide bias
24-29 nt reads with ping-pong signature and 10-nt overlaps
(Range:-300 to 300)
Nucleoprotein
(-)ssRNA genome

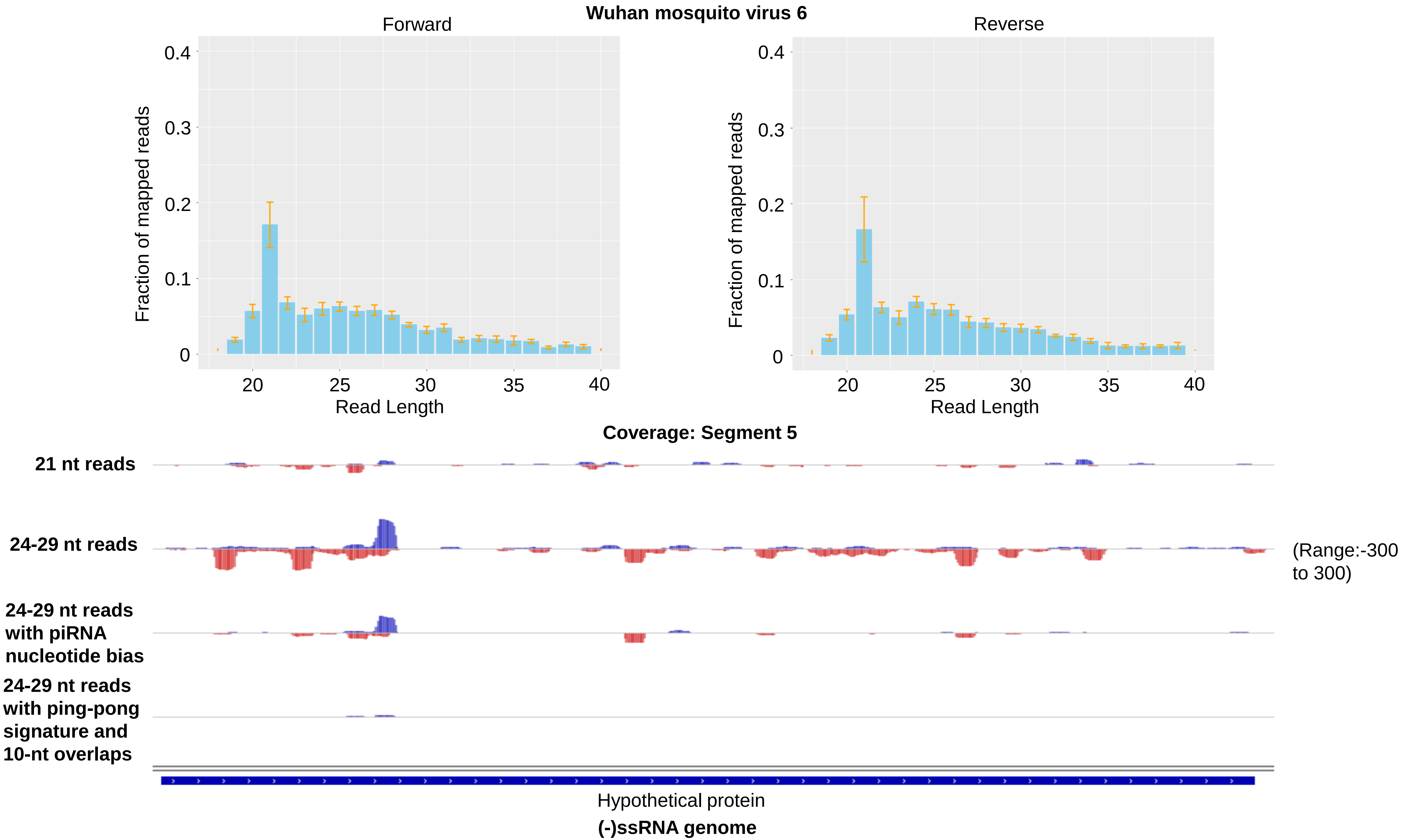
Wuhan mosquito virus 6
Forward
Reverse
Fraction of mapped reads
0.4
0.3
0.2
0.1
0
20
25
30
35
40
Read Length
Coverage: Segment 5
21 nt reads
24-29 nt reads
(Range:-300 to 300)
24-29 nt reads with piRNA nucleotide bias
24-29 nt reads with ping-pong signature and 10-nt overlaps
Hypothetical protein
(-)ssRNA genome

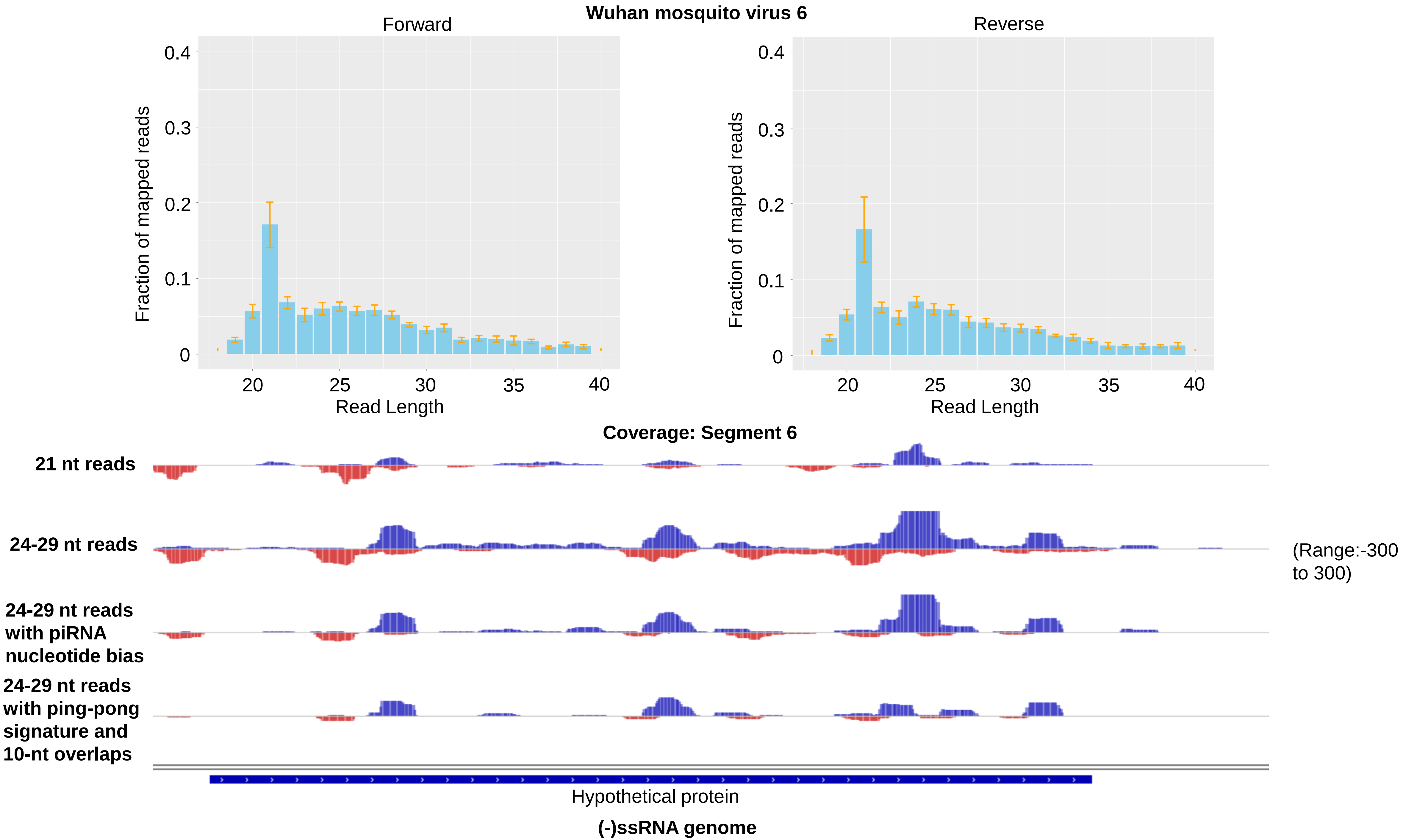

Wuhan mosquito virus 6
Forward
Reverse
Fraction of mapped reads
0.4
0.3
0.2
0.1
0
20
25
30
35
40
Read Length
Coverage: Segment 6
21 nt reads
24-29 nt reads
24-29 nt reads with piRNA nucleotide bias
24-29 nt reads with ping-pong signature and 10-nt overlaps
(Range:-300 to 300)
Hypothetical protein
(-)ssRNA genome

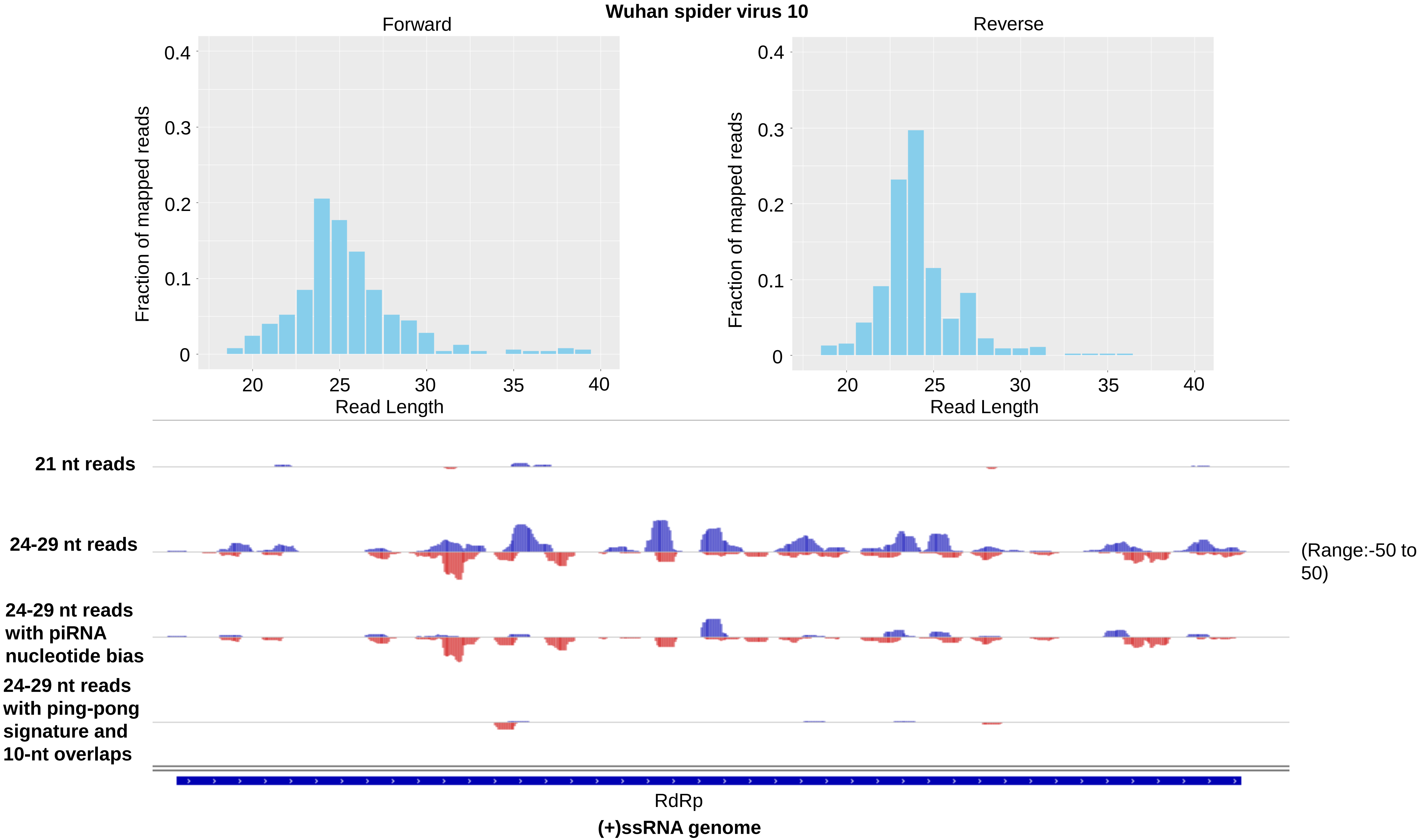

Wuhan spider virus 10
Forward
Reverse
Fraction of mapped reads
0.4
0.3
0.2
0.1
0
20
25
30
35
40
Read Length
21 nt reads
24-29 nt reads
24-29 nt reads with piRNA nucleotide bias
24-29 nt reads with ping-pong signature and 10-nt overlaps
(Range:-50 to 50)
RdRp
(+)ssRNA genome

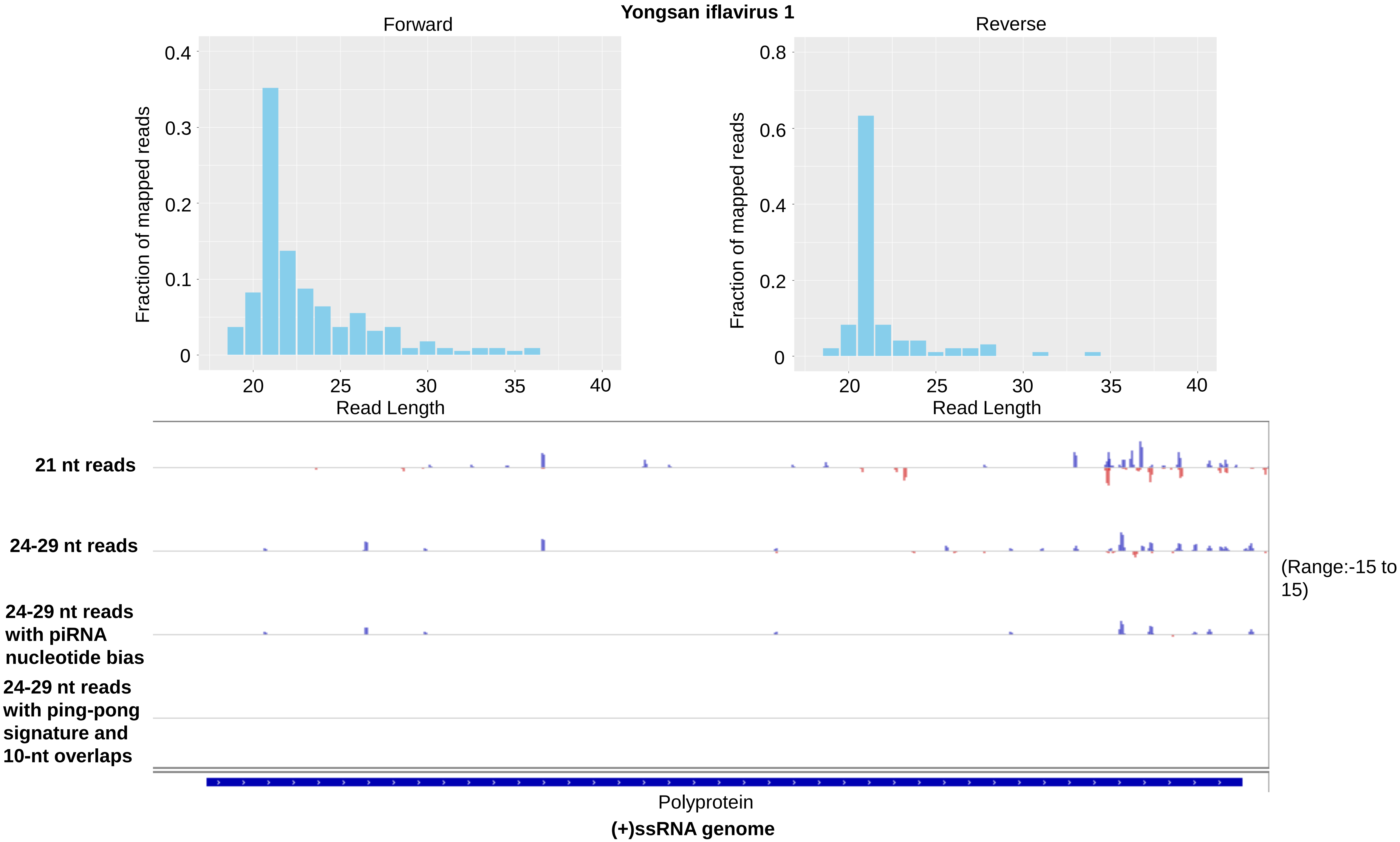
Yongsan iflavirus 1
Forward
Reverse
Fraction of mapped reads
Read Length
0.4
0.3
0.2
0.1
0
0.8
0.6
0.4
0.2
20
25
30
35
40
21 nt reads
24-29 nt reads
24-29 nt reads with piRNA nucleotide bias
24-29 nt reads with ping-pong signature and 10-nt overlaps
(Range:-15 to 15)
Polyprotein
(+)ssRNA genome

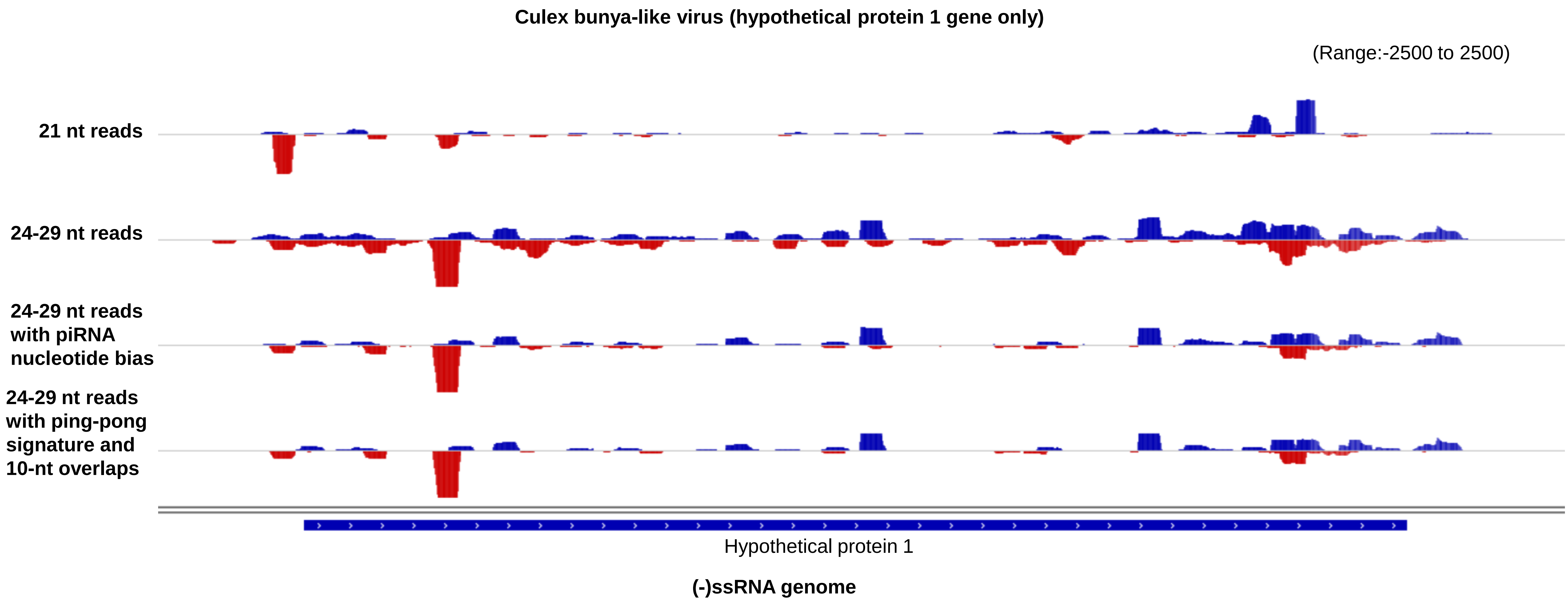

Culex bunya-like virus (hypothetical protein 1 gene only)
(Range:-2500 to 2500)
21 nt reads
24-29 nt reads
24-29 nt reads with piRNA nucleotide bias
24-29 nt reads with ping-pong signature and 10-nt overlaps
Hypothetical protein 1
(-)ssRNA genome

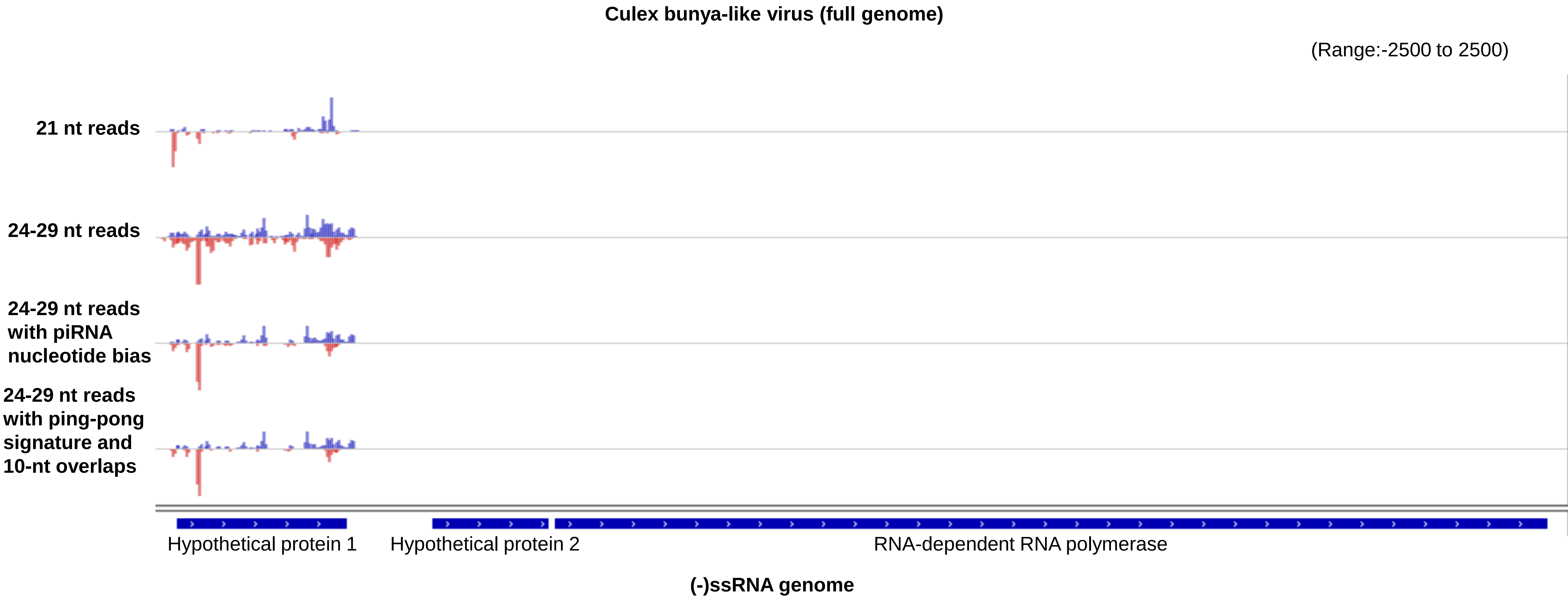
Culex bunya-like virus (full genome)
(Range:-2500 to 2500)
21 nt reads
24-29 nt reads
24-29 nt reads with piRNA nucleotide bias
24-29 nt reads with ping-pong signature and 10-nt overlaps
Hypothetical protein 1
Hypothetical protein 2
RNA-dependent RNA polymerase
(-)ssRNA genome

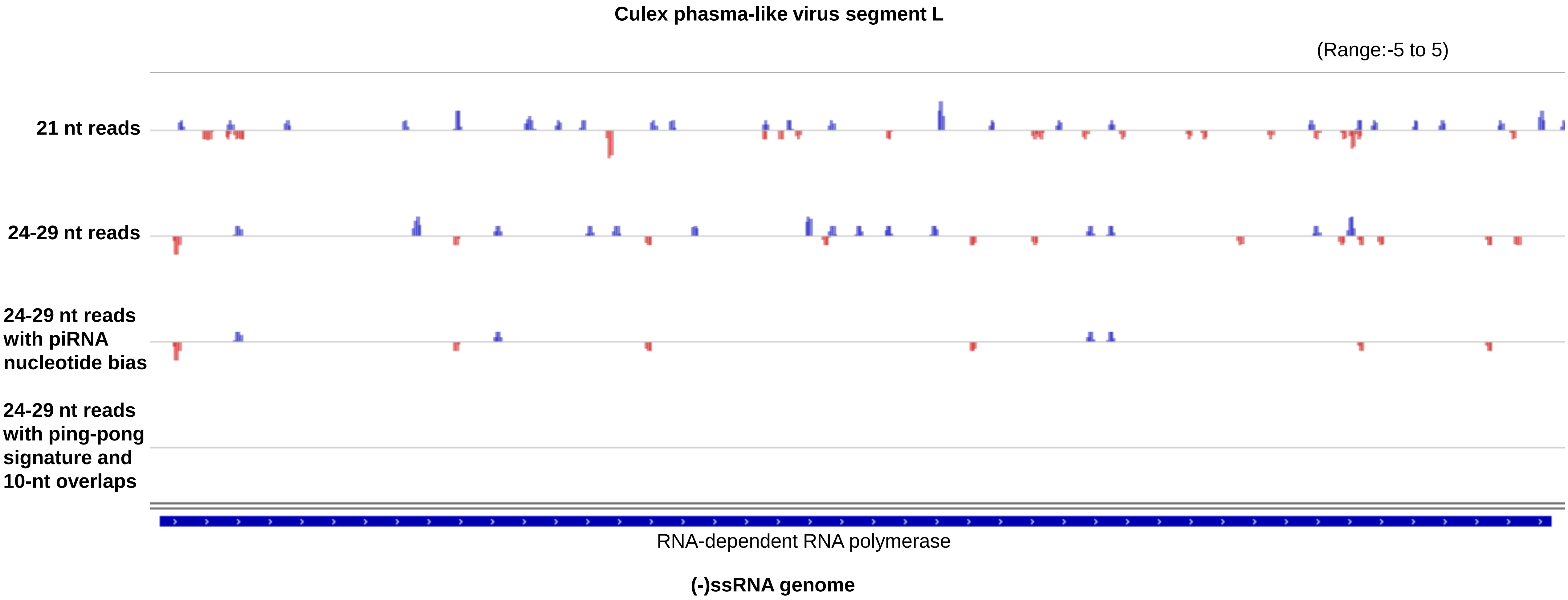
Culex phasma-like virus segment L
(Range:-5 to 5)
21 nt reads
24-29 nt reads
24-29 nt reads with piRNA nucleotide bias
24-29 nt reads with ping-pong signature and 10-nt overlaps
RNA-dependent RNA polymerase
(-)ssRNA genome

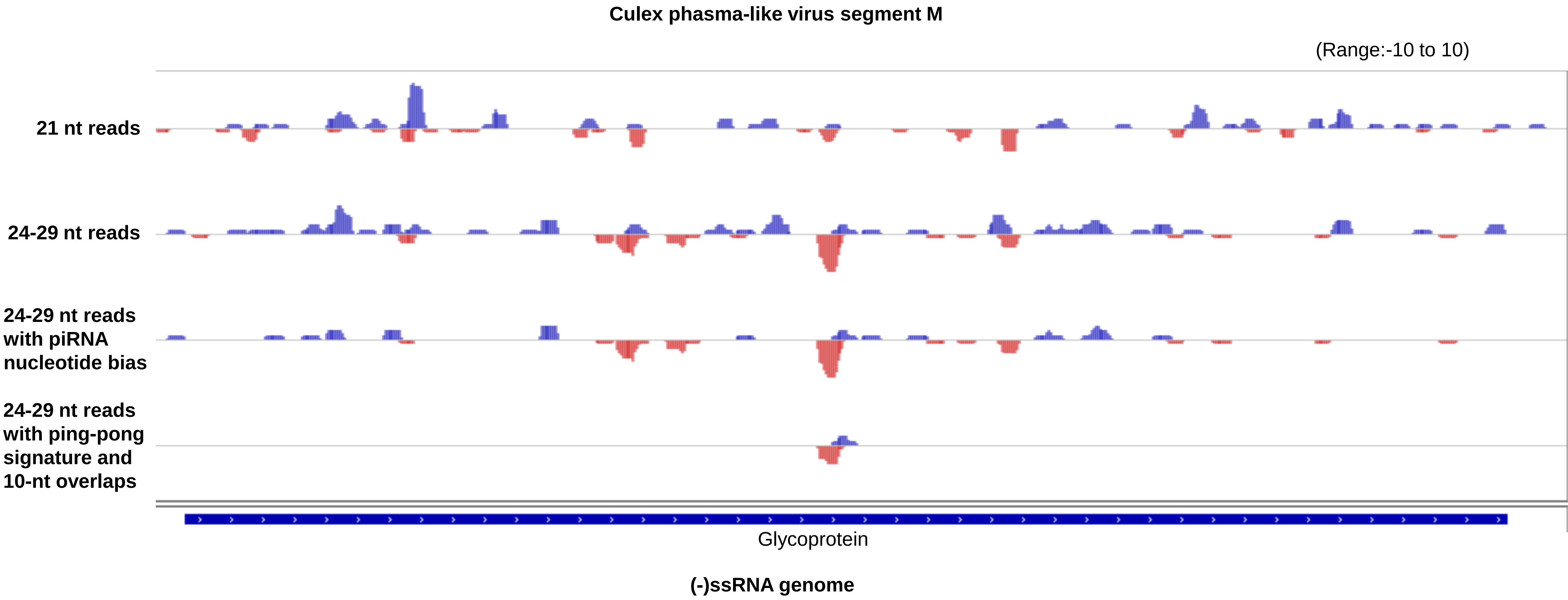
Culex phasma-like virus segment M
(Range:-10 to 10)
21 nt reads
24-29 nt reads
24-29 nt reads with piRNA nucleotide bias
24-29 nt reads with ping-pong signature and 10-nt overlaps
Glycoprotein
(-)ssRNA genome

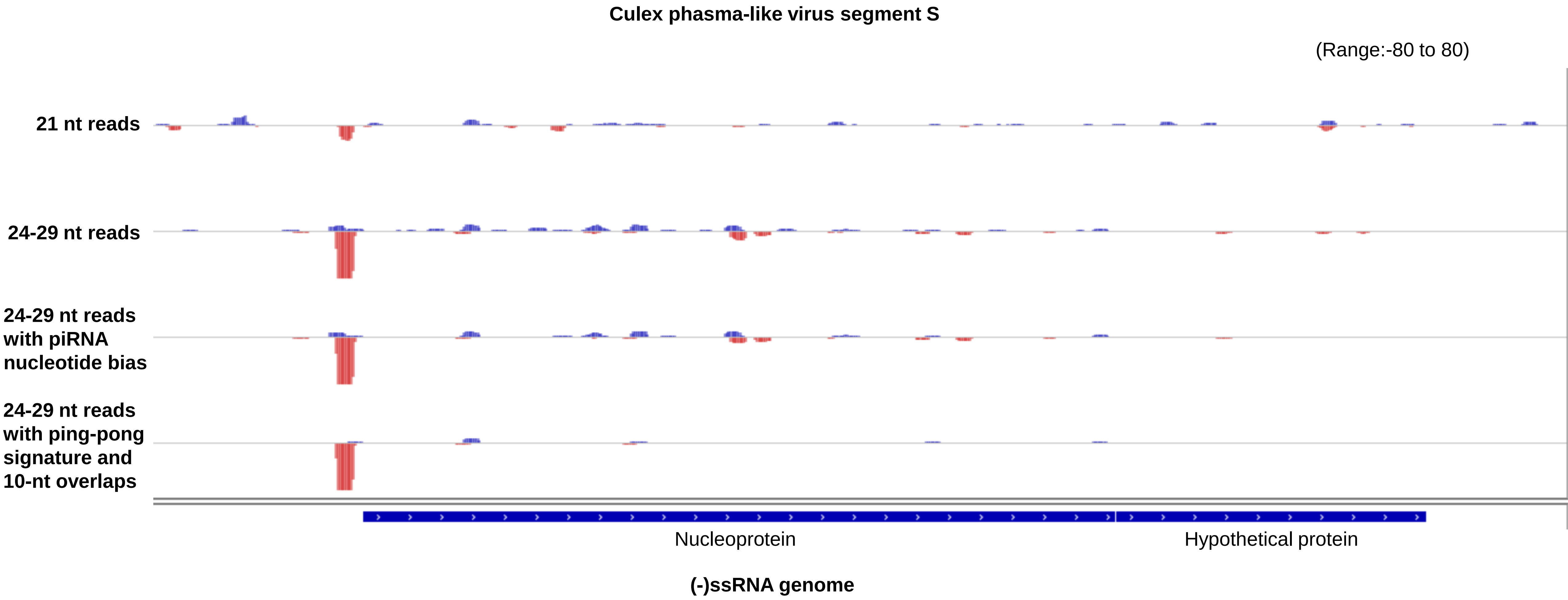
Culex phasma-like virus segment S
(Range:-80 to 80)
21 nt reads
24-29 nt reads
24-29 nt reads with piRNA nucleotide bias
24-29 nt reads with ping-pong signature and 10-nt overlaps
Nucleoprotein
Hypothetical protein
(-)ssRNA genome

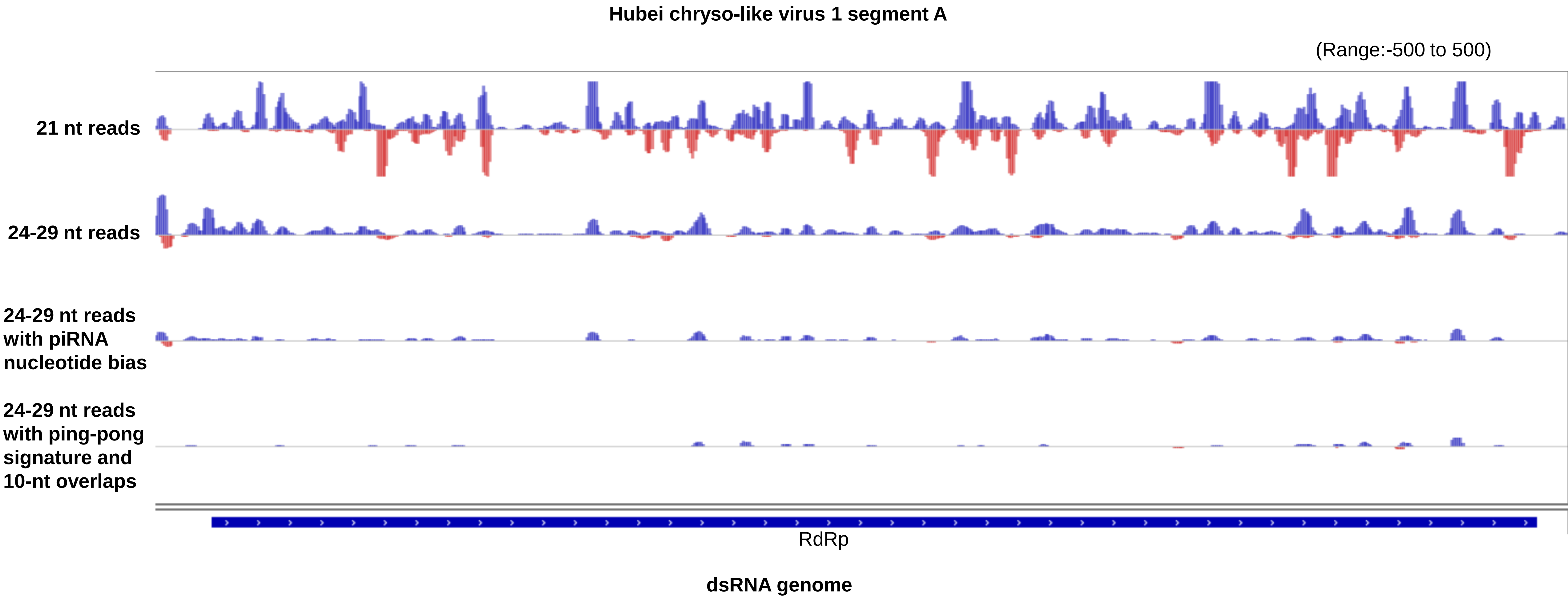
Hubei chryso-like virus 1 segment A
(Range:-500 to 500)
21 nt reads
24-29 nt reads
24-29 nt reads with piRNA nucleotide bias
24-29 nt reads with ping-pong signature and 10-nt overlaps
RdRp
dsRNA genome

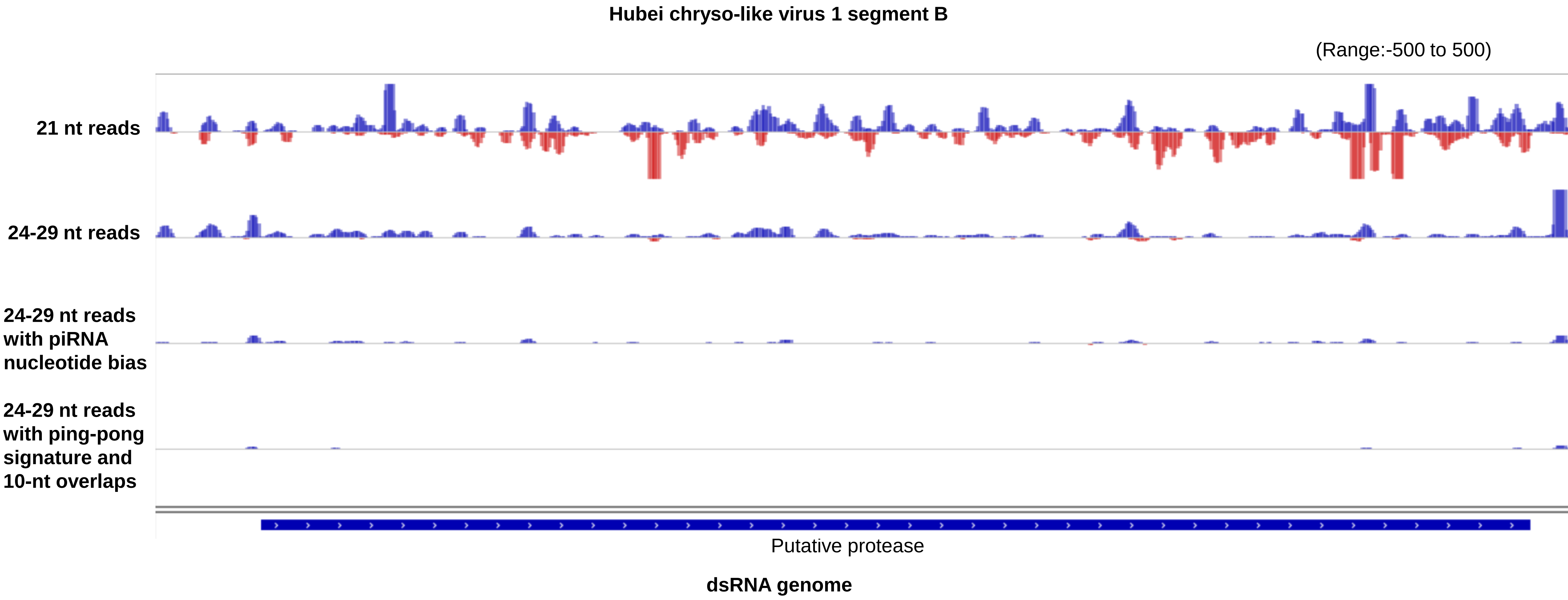
Hubei chryso-like virus 1 segment B
(Range:-500 to 500)
21 nt reads
24-29 nt reads
24-29 nt reads with piRNA nucleotide bias
24-29 nt reads with ping-pong signature and 10-nt overlaps
Putative protease
dsRNA genome

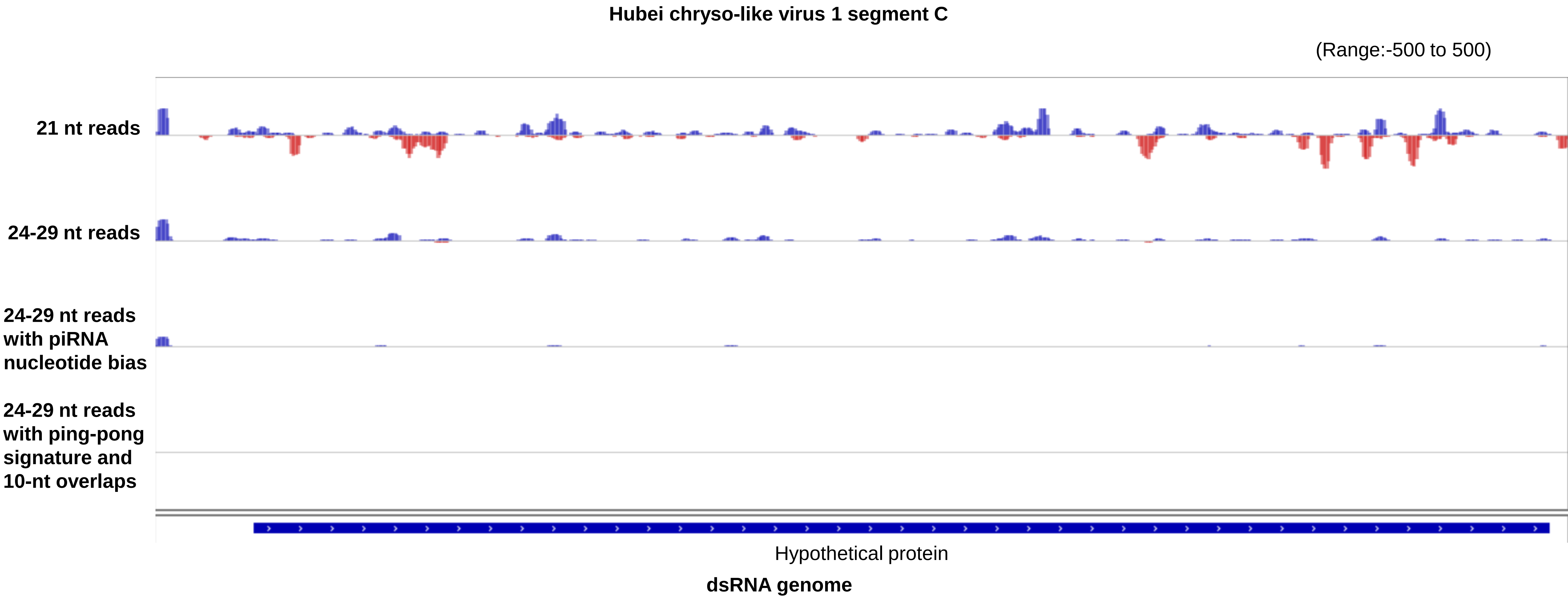
Hubei chryso-like virus 1 segment C
(Range:-500 to 500)
21 nt reads
24-29 nt reads
24-29 nt reads with piRNA nucleotide bias
24-29 nt reads with ping-pong signature and 10-nt overlaps
Hypothetical protein
dsRNA genome

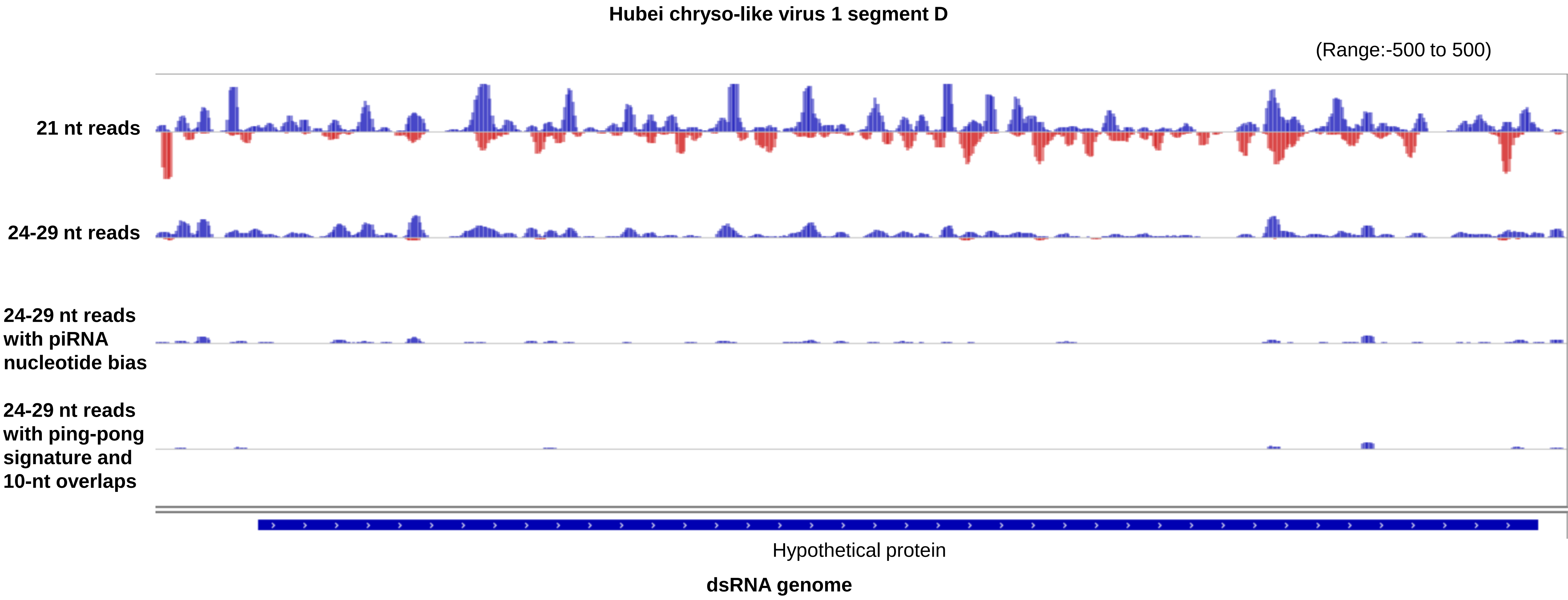
Hubei chryso-like virus 1 segment D
(Range:-500 to 500)
21 nt reads
24-29 nt reads
24-29 nt reads with piRNA nucleotide bias
24-29 nt reads with ping-pong signature and 10-nt overlaps
Hypothetical protein
dsRNA genome

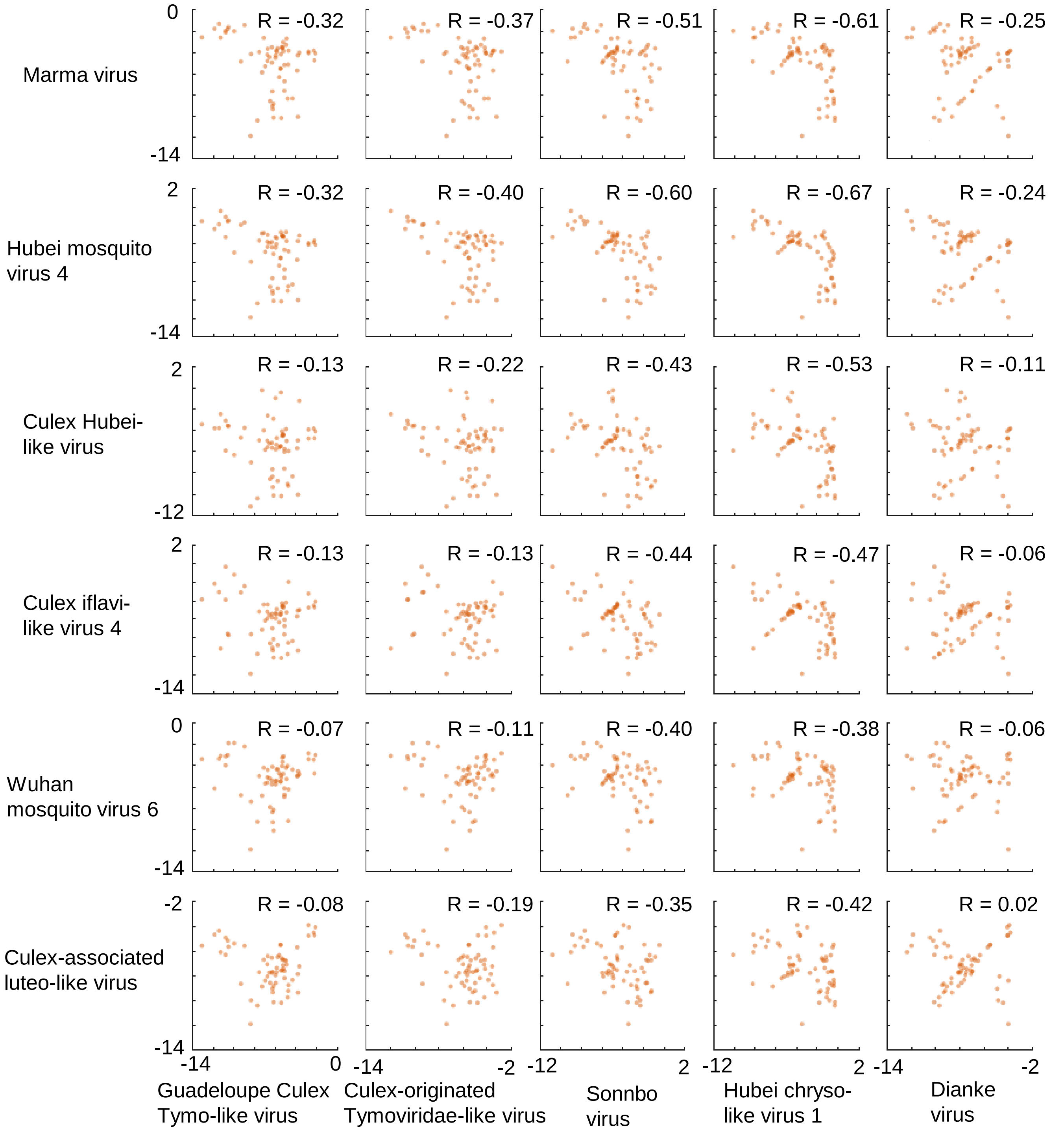

Fig. S4: Virus frequency (abundance) scatterplots for specific pairs of viruses. Each dot represents one pool sample, plotted by abundance of two specific viruses.

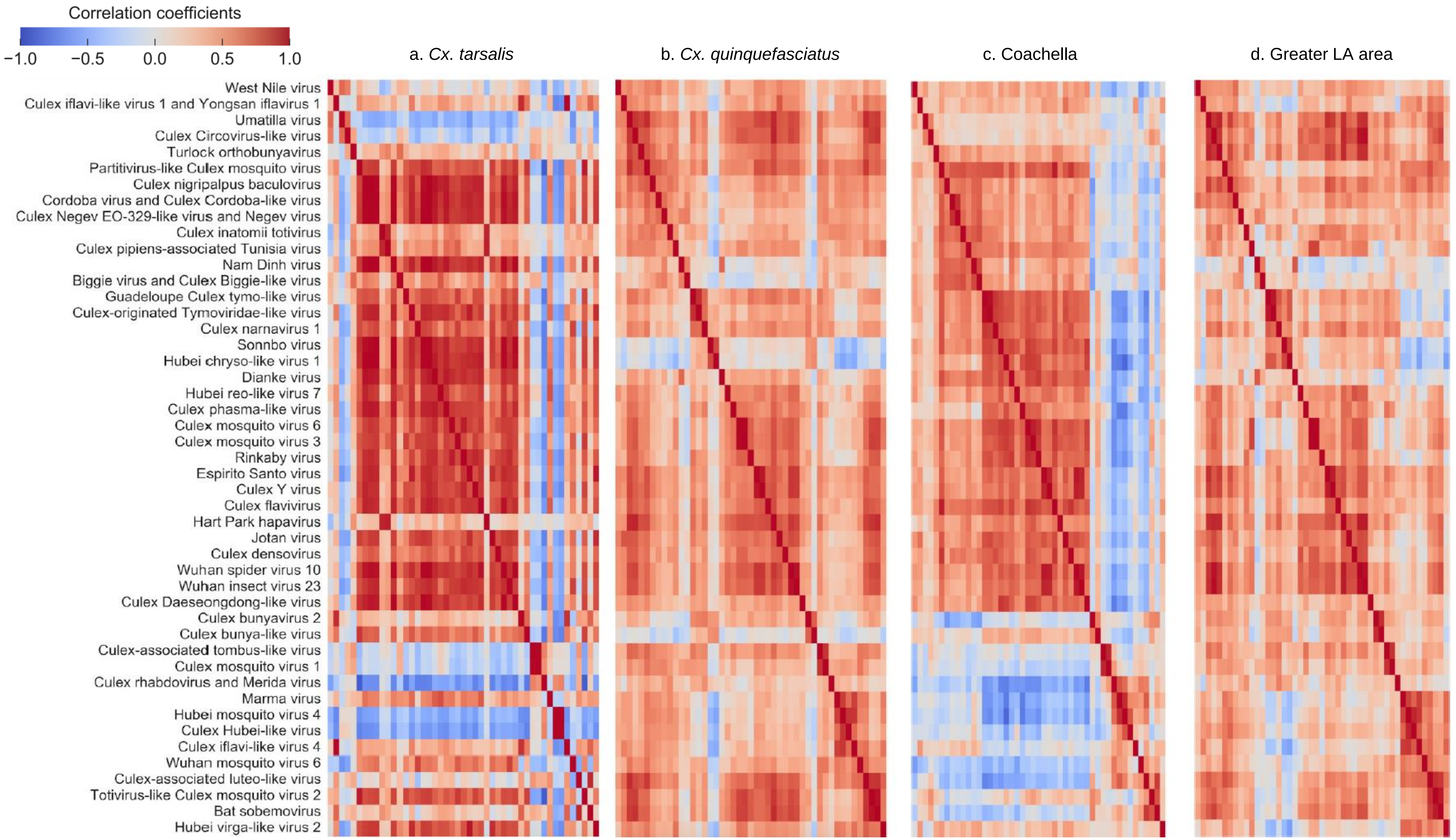

Fig. S5: Pearson correlation matrices with pools separated by location or species. Virus names for all matrices are in the same order as Figure 2. (a) Nine *Cx. tarsalis* field samples. (b) 48 field *Cx. quinquefasciatus* field samples. (c) 24 field samples from Coachella Valley. (d) 34 field samples from Greater LA area (all except 4 collected by West Valley MVCD).

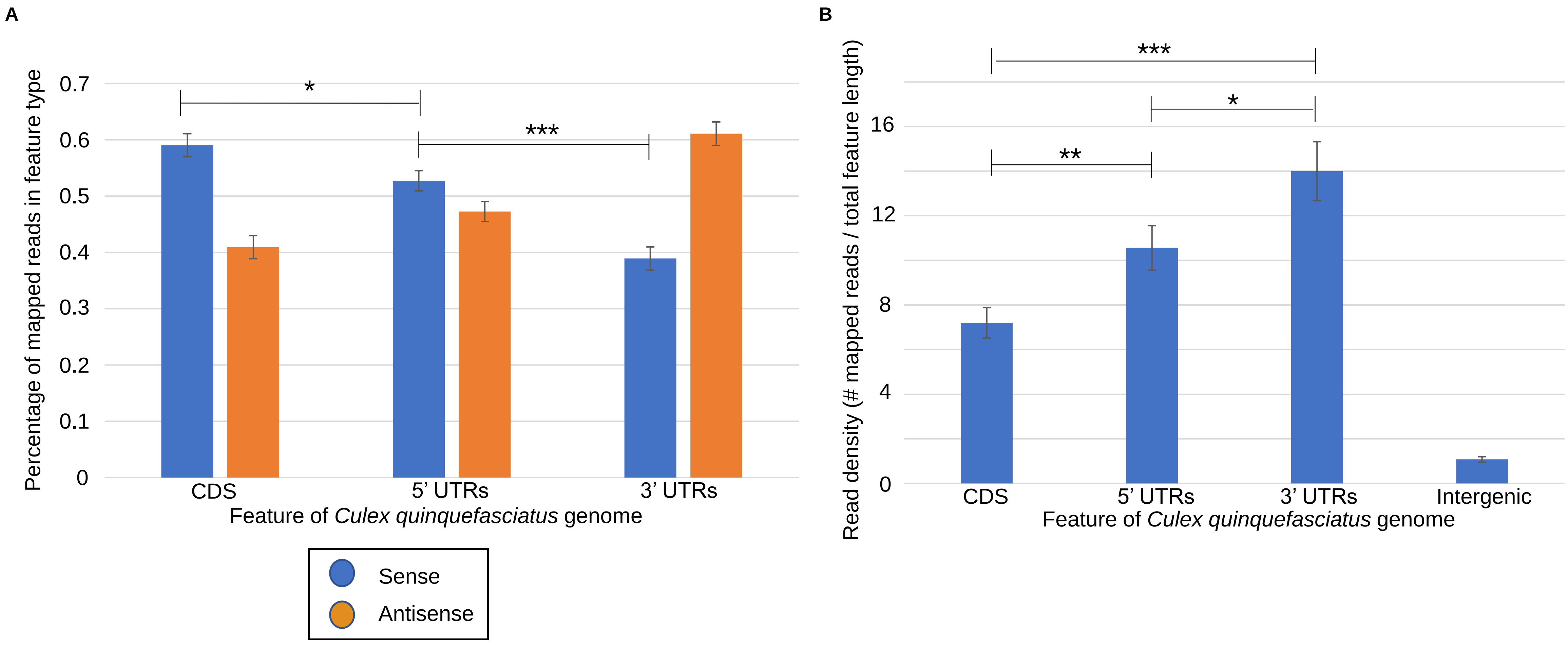

Fig. S6: Mapping of small RNA reads to features of the *Cx. quinquefasciatus* genome. Error bars show standard error across all *Cx. quinquefasciatus* samples. Asterisks show significance of t-tests comparing numbers of sense reads between features (*: 0.01 < P-value < 0.05, **:0.001 < P-value<0.01, ***: P-value < 0.001).

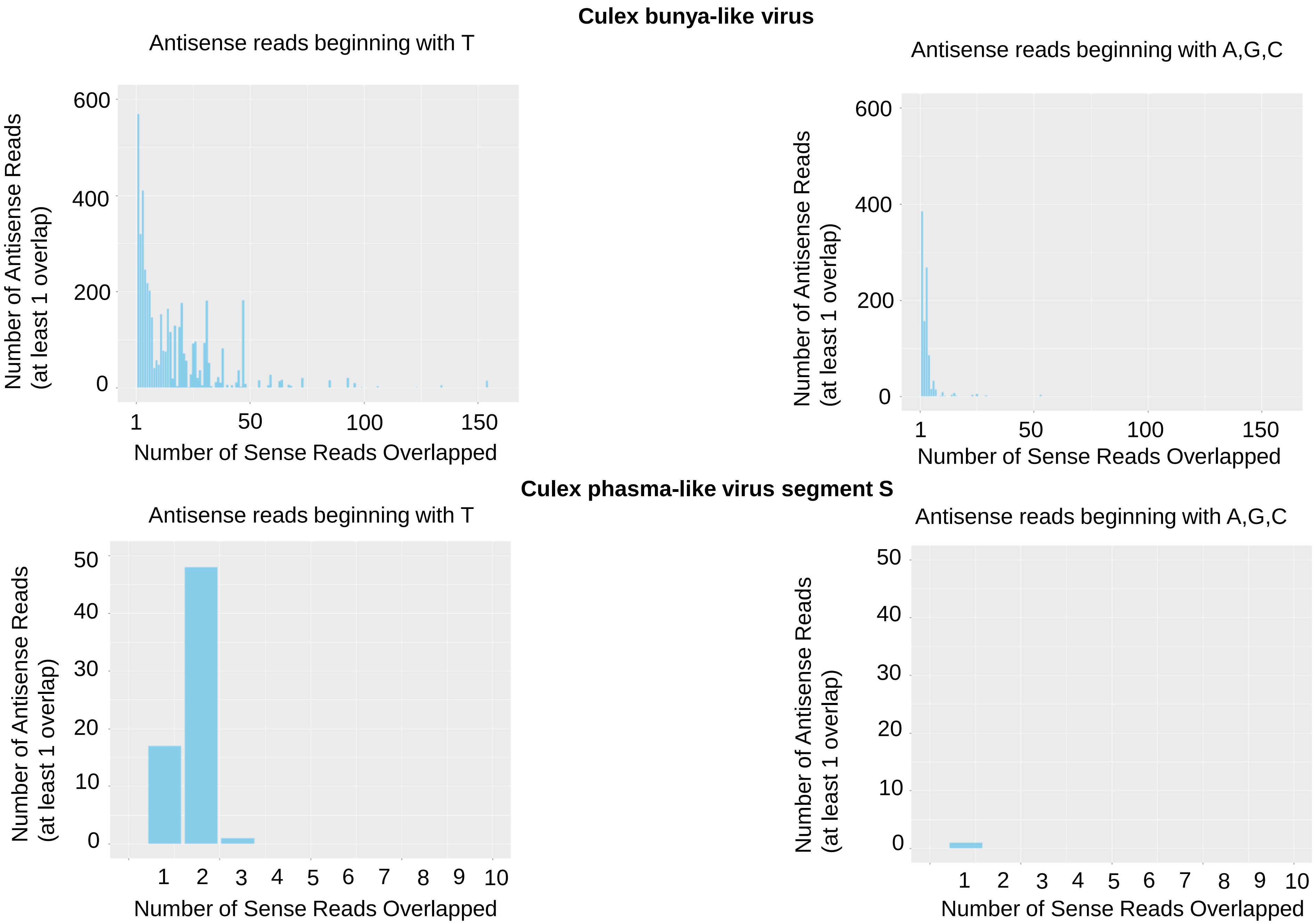

Fig. S7: Number of ten-nucleotide overlaps with sense reads for antisense-mapped 24-29 nt reads.

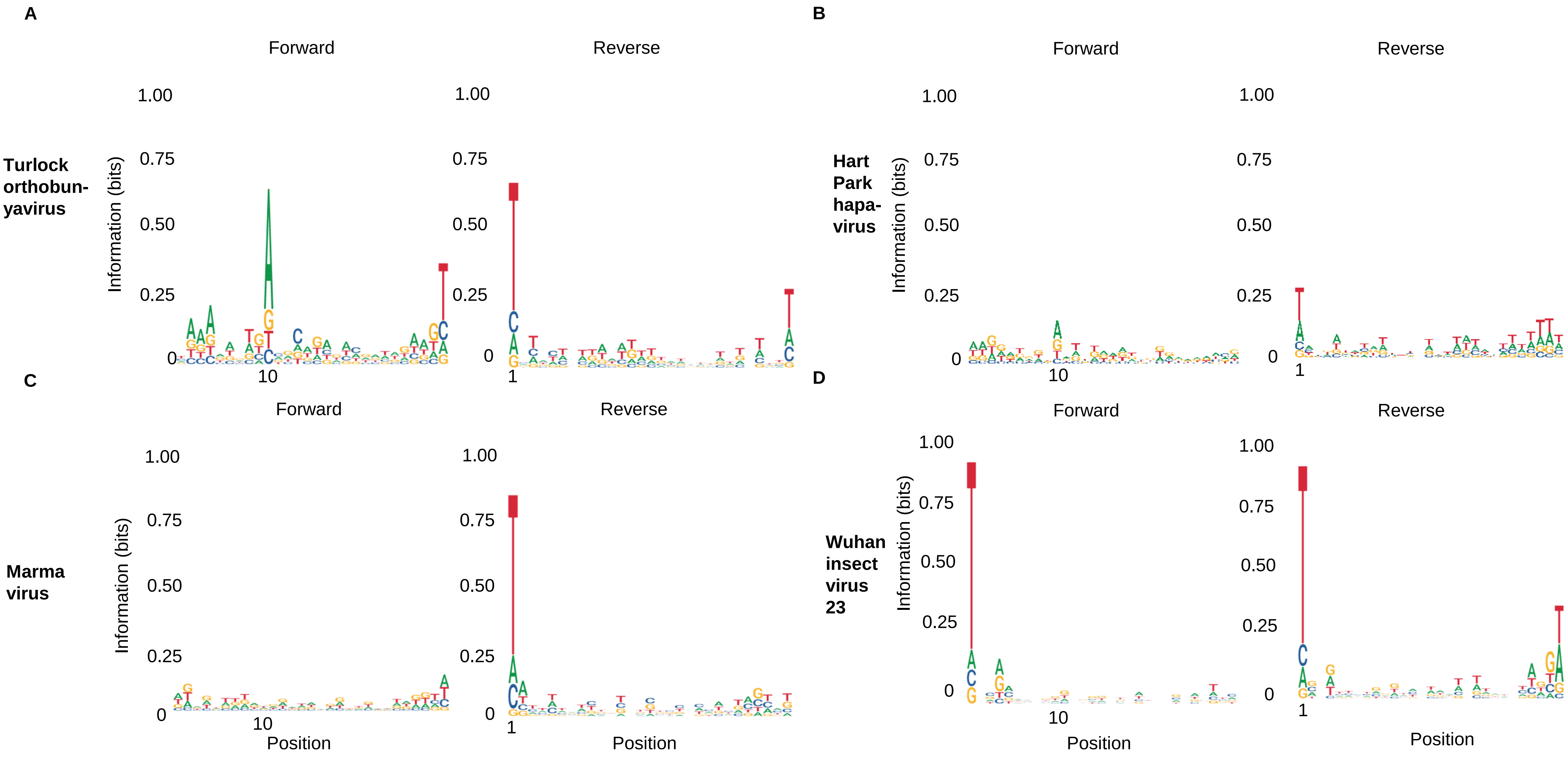

Fig. S8: Nucleotide bias of 24-29 nt reads for Turlock orthobunyavirus. Hart Park hapavirus, Marma virus, and Wuhan insect virus 23.

SUPPLEMENTARY MATERIALS AND METHODS

Mosquito collection, pooling, and nucleic acid extraction

For samples collected in Ontario area (southwestern corner of San Bernardino County, California), mosquitoes were amassed using CO_2 traps and gravid traps for host-seeking and egg-laying females respectively, by the West Valley MVCD during its routine mosquito and arbovirus surveillance operations. Collected mosquitoes were transported with icepacks and moist towels to the laboratory where they were identified, counted, and pooled by species, location, and collection date at 5-50 females per pool for further testing. Mosquito pools were then stored at -75°C deep freezer until further testing. Nucleic acid extraction was performed using the MagMAX Viral RNA Isolation Kit (Product No. AMB18365, Applied Biosystems) with the MagMAX Express 96 extraction system.

For samples collected in Coachella Valley area, mosquitoes were amassed using dry ice-baited CDC-style traps without lights (CO_2 traps) set for one night by the Coachella Valley Mosquito and Vector Control District service as part of the routine arbovirus surveillance [1]. In the residential zones of the Coachella Valley, gravid traps [2, 3] and dry ice-baited BG-Sentinel 2 traps (Biogents, Regensburg, Germany) were also used for one night. Mosquito collections were returned to the laboratory, anesthetized with triethylamine (TEA), and identified to species, sex, and physiological condition (unfed, bloodfed, or gravid). Five to 50 female mosquitoes were pooled by species for each trap site. Mosquito samples were extracted the same day or kept at -80°C to extract nucleic acid the following day or later. Nucleic acid extraction was completed using the

MagMAX Viral RNA Isolation Kit (AMB18365). Elution buffer with the final DNA/RNA product was stored at -80°C prior to shipping on dry ice for further processing.

RNA extraction and validation

The nucleic acid extracts were brought up to 100 µL with nuclease-free water. 4 volumes (400 µL) of TRIzol LS were added for long-term storage, and the samples were stored at -80°C until RNA extraction and size selection.

To begin RNA extraction, 5PRIME Phase Lock Gel Tubes (Quantabio #2302830-Heavy) were used for chloroform extraction. First, the tubes were centrifuged at 12,000 g for 30 seconds to bring down the phase gel. The samples were transferred to the phase lock gel tubes and incubated at room temperature for 5 minutes. Next, 0.27 times the TRIzol volume of chloroform (108 µL) was added to the samples, mixed well by inverting for 15 seconds, and incubated at RT for 2-3 minutes. Samples were then spun at 12,000 g for 15 min at 4°C to separate the phases, and the upper aqueous phase was transferred to a fresh Eppendorf tube. 0.7 times the TRIzol volume of cold isopropanol (280 µL) was added, followed by incubation on ice for 30 minutes. Samples were spun at 16,200 g for 15 minutes at 4°C, the supernatant was discarded, and the pellet was resuspended in 1.3 times the TRIzol volume (520 µL) of 75% ethanol. After spinning again for 5 minutes at 7500 g, 4°C, supernatant was discarded and the pellet was air dried for 10 minutes at RT, before resuspension in 50 µL of nuclease-free water.

Samples were then treated with 2 µL DNase I, with 5.65 µL of 10X DNase I buffer added, at 37°C for 1 hour. To inactivate DNase I, 50 mM EDTA was added to a final concentration of 1 mM and the samples were incubated at RT for 5 minutes.

At this point, as a quality check, 2-µL aliquots of each RNA sample were run on a 1.4% agarose gel. Sufficiently intact RNA samples were carried through the remaining part of the protocol for size selection and library preparation.

In order to purify the RNA, a cleanup with Agencourt RNAclean XP beads (Beckman Coulter #A63987) was performed, following the provided protocol for these beads.

The RNA quantity was then measured using a Nanodrop spectrophotometer before proceeding with small RNA library preparation.

Library preparation and sequencing

Library preparation was performed using the NEBNext Multiplex Small RNA Library Prep Set for Illumina (NEB #E7300S/L). The provided protocol for this kit was followed. First, in separate steps, the 3' SR Adaptor was ligated, the Reverse Transcription Primer was hybridized, and the 5' SR Adaptor was ligated. First strand cDNA synthesis was performed by reverse transcription, followed by PCR amplification (94°C 30 sec, 12-15 cycles of 94°C 15 sec, 62°C 30 sec, 70°C 15 sec; 70°C for 5 min, 4°C hold). The PCR product was purified using the QIAquick PCR purification kit (QIAGEN #28106).

Size selection was performed on a 6% TBE PAGE gel where each cDNA sample was split in half into adjacent wells. From this gel, slices corresponding to the cDNA fragments between approximately 140 bp and 150 bp (representing small RNAs between 20 and 30 nt) were isolated. The cDNA in these slices was eluted and precipitated. The air-dried pellets were resuspended in 12 µL TE buffer. The cDNA

libraries were assessed on an Agilent 2100 Bioanalyzer before being sequenced on an Illumina sequencing platform.

Initial read processing and viral detection

The Illumina sequencing results were downloaded in FASTQ form. For the samples that were sequenced in paired-end fashion, only one of the two theoretically equivalent (for small RNA) sets of reads was utilized. Adapter sequences were removed using Trimmomatic [4], with verification of removal using FastQC [5]. For mapping to viruses to examine patterns based on viral abundances, for analysis of mosquito-mapped reads, and for analysis beyond viral detection, only reads of length 18 bp or higher were retained.

Adapter-trimmed reads from each sample were used for viral detection. These reads were input to VirusDetect, an automated pipeline designed for virus discovery using deep sequencing of small RNAs [6]. This pipeline uses two separate methods for contig assembly. First, reads are aligned to a viral genome database using BWA, and the aligned reads are assembled by reference-guided assembly. Second, reads are aligned to the host (in this case *Cx. quinquefasciatus)* genome, and unaligned reads are assembled *de novo* using Velvet, with k-mer length and coverage cutoff parameters optimized empirically. Assembled contigs from both methods are compared to a curated list of viral sequences by BLAST using both blastn (nucleotide) and blastx (amino acid) alignment for detection. We treated a match as a high-identity match if the average identity of the contigs was 90% nucleotide identity or higher by blastn, and these viruses were used for analysis of viral abundances and mosquito small RNA response.

Clustering and prediction based on viral abundances

We mapped all small RNA reads longer than 18 nt to the *Cx. quinquefasciatus* (CpipJ2) genome using Bowtie1 [7] with default settings. This was done for *Cx. tarsalis* samples as well to remove as many host-derived reads as possible, as no *Cx. tarsalis* genome assembly was available at the time of analysis. Mapped reads were depleted, and the remaining reads were mapped against a combined file containing all virus genomes that had been detected with high confidence by VirusDetect. This mapping was done using Bowtie2 [8] with parameters -N 1 -L 10 -i S,1,0.50 –gbar 2 -D 50 -R 5, in order to allow more mismatches in the alignments. All reads that could have mapped equally well to multiple viruses, as determined by the alignment having a mapping quality of 0 or 1, were removed using SAMtools [9]. This left a set of uniquely mapped reads that mapped with high confidence to only one of the detected viruses. Each mosquito sample's descriptive information and uniquely mapped virus read counts were recorded in Supplementary Table S1.

We then converted read counts to frequencies for every sample, as the number of small RNA reads, and uniquely mapped virus reads vary differently in scale across different samples. For each sample, after adding a pseudocount to every virus read count, the number of reads uniquely mapped to each virus in one sample were divided by the total number of reads mapped to all viruses in the same sample. The frequencies were collected into a 62 × 45 matrix (total 62 samples and 45 known viruses), where each row of the matrix represents one sample's virus distribution. The frequencies were log-transformed for the consistency of correlations between samples.

We used Uniform Manifold Approximation and Projection (UMAP), a dimension reduction technique [10], to generate a lower dimensional visualization plot for the high dimensional virus frequency matrix. When running UMAP, we chose appropriate hyperparameters (Supplementary Table S8), and obtained two-dimensional embedding representations which clustered according to sample features.

For more details about viral infection patterns, we generated and inspected Pearson correlation matrices for samples and viruses. Each entry in the correlation matrix is the Pearson correlation coefficient between two variables (samples or viruses). Variables in the sample matrix are the different virus frequencies in each sample and the variables in the virus matrix are virus frequencies in different samples.

Analysis of mosquito-mapped small RNA reads and miRNA analysis

Adapter-trimmed reads for each sample were filtered to retain only reads of length 18 bp or higher. Due to the lack of an extensively annotated genome assembly for *Cx. tarsalis*, only *Cx. quinquefasciatus* samples were used for this analysis. The *Cx. quinquefasciatus* genome assembly (CpipJ2, Johannesburg strain) and gene set (CpipJ2.4) were downloaded from VectorBase (https://vectorbase.org). Because many of the protein-coding genes do not have annotated 5’ and 3’ UTRs, the remaining UTRs were estimated with lengths 300 bp for 5’ UTRs and 400 bp for 3’ UTRs. These values were chosen to be slightly higher than the median UTR lengths previously observed in fruit flies and mosquitoes [11, 12]. The protein-coding genes were used only for classifying reads by location of origin in the mosquito genome, not for differential expression analysis.

Reads were aligned to the *Cx. quinquefasciatus* genome using Bowtie1 with default settings. Over 93% of total sequenced reads aligned to the *Culex* genome. Bedtools multicov [13] was used to count the number of reads that aligned to genomic regions. Percentages of reads mapping to types of genomic features were determined by summing reads from all 48 field samples and all 5 lab samples.

For miRNA differential expression analysis, we used bedtools multicov to count the number of reads that aligned to each pre-miRNA gene, The analysis was done using DESeq2 [14] with only sense-mapped reads, which constituted the vast majority of reads mapped to pre-miRNA genes.

In order to compare random groups of samples against each other, all 48 *Cx. quinquefasciatus* field samples were evenly assigned randomly into two groups using a random number generator, 5 times. When comparing field against lab samples, all 48 *Cx. quinquefasciatus* field samples were assigned to one group and all 5 *Cx. quinquefasciatus* lab samples to the other. For comparison by viral infection, percentage of length 18+ bp reads that aligned to any of the detected viruses (except Culex narnavirus 1) was used to determine whether a sample was highly or lowly infected by viruses. If a sample had over 0.049% of reads aligned to these viruses, it was assigned to the highly infected group, while otherwise it was assigned to the lowly infected group. Similarly, to compare samples by abundance of CxNV1, the cutoff was 0.171% (the median percentage) of length 18+ bp reads mapped to the CxNV1 genome. To determine whether a sample was highly or lowly infected by *Wolbachia*, we used the percentage of *Culex*-unmapped reads that aligned to the *Wolbachia Culex* endosymbiont genome. If a sample had above the median percentage of reads (6.34%)

aligned to *Wolbachia*, it was assigned to the highly infected group, and otherwise it was assigned to the lowly infected group.

For all of these analyses, a miRNA gene was considered to be significantly differentially expressed if the adjusted P-value from DESeq2 was less than 0.05 and the log2fold change between sample groups was greater than 1 or less than -1. R package EnhancedVolcano [15] was used to produce volcano plots based on DESeq2 results.

Analysis of small RNA response to specific viruses

Adapter-trimmed reads for each sample were filtered to retain only reads of length 18 bp or higher. These reads were aligned to a combined file containing all viral genomes that were detected previously by the assembly method. Alignment was done using Bowtie2 [8], with parameters -N 1 -L 10 -i S,1,0.50 –gbar 2 -D 50 -R 5, in order to allow more mismatches in the alignments. Using SAMtools [9], only reads with mapping quality 2 or higher were retained in order to filter out reads that aligned equally well to multiple viral genomes. Using custom Python scripts, we generated the size profiles of reads that mapped uniquely to each virus, converted these counts for each size into percentages of total mapped reads, and combined this information for all samples in which a virus had been detected by VirusDetect, calculating the average and standard deviation across all samples. For Culex phasma-like virus, one sample (D4) was included although the virus had not been detected by VirusDetect in that sample, because of a high number of mapped reads by Bowtie2. Histograms showing the size profiles were plotted in R. Error bars show the average percentage of reads +/- the

standard error, giving an indication of how well samples agreed at that size for that virus.

The ggseqlogo package [16] in R was used to show the nucleotide bias representing the ping-pong signature of piRNAs. A Python script calculated the numbers of 10-nt overlaps between small RNAs of length 24-29 bp, and identified the small RNAs that had the piRNA nucleotide bias and at least one 10-nt overlap with a small RNA of the opposite orientation (sense vs. antisense). These identified reads were then used, together with all 21 nt and all 24-29 nt reads, to generate coverage plots for each virus that had been detected by VirusDetect, using the Integrative Genomics Viewer (IGV) [17]. These coverage plots combine reads from all samples for which that virus was detected. We used SAMtools [9] to segregate sense and antisense reads into separate tracks which were overlaid on IGV, and for various other tasks involved in processing alignment files.

References for Supplementary Methods

1. Reisen WK, Lothrop HD (1999) Effects of Sampling Design on the Estimation of Adult. J Am Mosq Control Assoc 15:105–114

2. Cummings RF (1992) Design and use of a modified Reiter gravid mosquito trap for mosquito-borne encephalitis surveillance in Los Angeles County, California. Proc Mosq Vector Control Assoc Calif 60:170–176

3. Lothrop HD, Lothrop BB, Gomsi DE, Reisen WK (2008) Intensive early season adulticide applications decrease arbovirus transmission throughout the Coachella Valley, Riverside County, California. Vector-Borne Zoonotic Dis 8:475–489. https://doi.org/10.1089/vbz.2007.0238

4. Bolger AM, Lohse M, Usadel B (2014) Trimmomatic: A flexible trimmer for Illumina sequence data. Bioinformatics 30:2114–2120. https://doi.org/10.1093/bioinformatics/btu170

5. Andrews S (2010) FastQC: A Quality Control Tool for High Throughput Sequence Data

6. Zheng Y, Gao S, Padmanabhan C, et al (2017) VirusDetect: An automated pipeline for efficient virus discovery using deep sequencing of small RNAs. Virology 500:130–138. https://doi.org/10.1016/j.virol.2016.10.017

7. Langmead B, Trapnell C, Pop M, Salzberg SL (2009) Ultrafast and memory-efficient alignment of short DNA sequences to the human genome. Genome Biol 10:. https://doi.org/10.1186/gb-2009-10-3-r25

8. Langmead B, Salzberg SL (2012) Fast gapped-read alignment with Bowtie 2. Nat Methods 9:357–359. https://doi.org/10.1038/nmeth.1923

9. Li H, Handsaker B, Wysoker A, et al (2009) The Sequence Alignment/Map format and SAMtools. Bioinformatics 25:2078–2079. https://doi.org/10.1093/bioinformatics/btp352

10. McInnes L, Healy J, Melville J (2018) UMAP: Uniform Manifold Approximation and Projection for Dimension Reduction

11. Chen CH, Lin HY, Pan CL, Chen FC (2011) The length evolution of 5′ untranslated regions - The stochastic model revisited. Proc - 2011 11th IEEE Int Conf Bioinforma Bioeng BIBE 2011 162–166. https://doi.org/10.1109/BIBE.2011.32

12. Chen CY, Chen ST, Juan HF, Huang HC (2012) Lengthening of 3′UTR increases with morphological complexity in animal evolution. Bioinformatics 28:3178–3181. https://doi.org/10.1093/bioinformatics/bts623

13. Quinlan AR, Hall IM (2010) BEDTools: A flexible suite of utilities for comparing genomic features. Bioinformatics 26:841–842. https://doi.org/10.1093/bioinformatics/btq033

14. Love MI, Huber W, Anders S (2014) Moderated estimation of fold change and dispersion for RNA-seq data with DESeq2. Genome Biol 15:1–21. https://doi.org/10.1186/s13059-014-0550-8

15. Blighe K, Rana S, Lewis M EnhancedVolcano: Publication-ready volcano plots with enhanced colouring and labeling

16. Wagih O (2017) Ggseqlogo: A versatile R package for drawing sequence logos. Bioinformatics 33:3645–3647. https://doi.org/10.1093/bioinformatics/btx469

17. Thorvaldsdóttir H, Robinson JT, Mesirov JP (2013) Integrative Genomics Viewer (IGV): High-performance genomics data visualization and exploration. Brief Bioinform 14:178–192. https://doi.org/10.1093/bib/bbs017
